# Fibroblast Growth Factor 2 (FGF2) Activates Vascular Endothelial Growth Factor (VEGF) Signaling in Gastrointestinal Stromal Tumors (GIST): An Autocrine Mechanism Contributing to Imatinib Mesylate (IM) Resistance

**DOI:** 10.3390/cancers16173103

**Published:** 2024-09-07

**Authors:** Sergei Boichuk, Pavel Dunaev, Aigul Galembikova, Elena Valeeva

**Affiliations:** 1 Department of Pathology, Kazan State Medical University, Kazan 420012, Russia; dunaevpavel@mail.ru (P.D.); ailuk000@mail.ru (A.G.); 2 Department of Radiotherapy and Radiology, Faculty of Surgery, Russian Medical Academy of Continuous Professional Education, Moscow 125993, Russia; 3 ”Biomarker” Research Laboratory, Institute of Fundamental Medicine and Biology, Kazan Federal University, Kazan 420008, Russia; 4 Central Research Laboratory, Kazan State Medical University, Kazan 420012, Russia; vevaleeva@ya.ru

**Keywords:** gastrointestinal stromal tumors (GISTs), imatinib mesylate (IM), sunitinib malate (SU), regorafenib (REGO), resistance, receptor tyrosine kinase inhibitor (RTKi), VEGF and FGF signaling, FGF2, VEGF-A

## Abstract

**Simple Summary:**

The FGF/FGFR-mediated pathway coordinates the activity of VEGF/VEGFR signaling in IM-resistant GISTs. Mechanistically, FGF2 triggers the activation of the FGFR pathway, turns on the activation of VEGFR signaling via the overproduction of VEGF-A, induces the interaction between FGFR1/2 and VEGFR1, and thereby renders cancer cells highly sensitive to the dual inhibition of the aforementioned RTKs. This was observed in IM-resistant GIST T-1R cells acquired IM resistance due to “RTK switch” (KIT loss/FGFR activation) and led to the impressive synergy scores between FGFR and VEGFR inhibitors, thereby providing a rationale to re-evaluate the effectiveness of combined anti-FGFR and VEGFR therapeutic strategies for GIST-acquired IM resistance via KIT-independent mechanisms.

**Abstract:**

We showed previously that the autocrine activation of the FGFR-mediated pathway in GIST lacking secondary *KIT* mutations was a result of the inhibition of KIT signaling. We show here that the FGF2/FGFR pathway regulates VEGF-A/VEGFR signaling in IM-resistant GIST cells. Indeed, recombinant FGF2 increased the production of VEGF-A by IM-naive and resistant GIST cells. VEGF-A production was also increased in KIT-inhibited GIST, whereas the neutralization of FGF2 by anti-FGF2 mAb attenuated VEGFR signaling. Of note, BGJ 398, pan FGFR inhibitor, effectively and time-dependently inhibited VEGFR signaling in IM-resistant GIST T-1R cells, thereby revealing the regulatory role of the FGFR pathway in VEGFR signaling for this particular GIST cell line. This also resulted in significant synergy between BGJ 398 and VEGFR inhibitors (i.e., sunitinib and regorafenib) by enhancing their pro-apoptotic and anti-proliferative activities. The high potency of the combined use of VEGFR and FGFR inhibitors in IM-resistant GISTs was revealed by the impressive synergy scores observed for regorafenib or sunitinib and BGJ 398. Moreover, FGFR1/2 and VEGFR1/2 were co-localized in IM-resistant GIST T-1R cells, and the direct interaction between the aforementioned RTKs was confirmed by co-immunoprecipitation. In contrast, IM-resistant GIST 430 cells expressed lower basal levels of FGF2 and VEGF-A. Despite the increased expression VEGFR1 and FGFR1/2 in GIST 430 cells, these RTKs were not co-localized and co-immunoprecipitated. Moreover, no synergy between FGFR and VEGFR inhibitors was observed for the IM-resistant GIST 430 cell line. Collectively, the dual targeting of FGFR and VEGFR pathways in IM-resistant GISTs is not limited to the synergistic anti-angiogenic treatment effects. The dual inhibition of FGFR and VEGFR pathways in IM-resistant GISTs potentiates the proapoptotic and anti-proliferative activities of the corresponding RTKi. Mechanistically, the FGF2-induced activation of the FGFR pathway turns on VEGFR signaling via the overproduction of VEGF-A, induces the interaction between FGFR1/2 and VEGFR1, and thereby renders cancer cells highly sensitive to the dual inhibition of the aforementioned RTKs. Thus, our data uncovers the novel mechanism of the cross-talk between the aforementioned RTKs in IM-resistant GISTs lacking secondary *KIT* mutations and suggests that the dual blockade of FGFR and VEGFR signaling might be an effective treatment strategy for patients with GIST-acquired IM resistance via KIT-independent mechanisms.

## 1. Introduction

Gastrointestinal stromal tumors (GISTs) are the most common mesenchymal malignancies of the gastrointestinal tract (GI) arising from the specialized interstitial cells of Cajal (ICCs), exhibiting the pacemaker activity to control the GI’s motility. Given that, the auto-activated, mutant *KIT* receptor tyrosine kinase gene or platelet-derived growth factor receptor alpha (*PDGFR-α*) are known as the most frequent driving forces of GIST development and pathogenesis [1,2,3]. Imatinib mesylate (IM) (Gleevec), the non-selective receptor tyrosine kinase inhibitor (RTKi), is currently accepted as the first-line therapy for GIST patients with a reported response rate (RR) of ~50–70% and progression-free survival (PFS) of ~20 months [4,5,6]. Despite the impressive response rates of the IM-based therapy for GISTs, the majority of patients with the advanced and metastatic form of the disease eventually acquire resistance to this targeted drug due to multiple molecular mechanisms, primarily including secondary downstream mutations of the aforementioned RTKs [7,8,9]. Sunitinib (Sutent) and regorafenib (Stivarga), known as the multi-target kinase inhibitors for VEGFR, PDGFR, Kit, etc., were approved for the second and third lines of GIST treatment, respectively. However, the clinical benefits of these therapies were numerally smaller. Indeed, sunitinib demonstrated an RR of 6.8% and a median PFS of 5.6 months, while regorafenib resulted in an RR of 4.5% and a PFS of 4.8 months [10,11]. Additionally, ripretinib (Qinlock) and avapritinib (Ayvakit) were approved in 2020 by the FDA for late-line therapies of GISTs. Ripretinib, a tyrosine switch-control inhibitor stabilizing the switch pocket in an inactive conformation, has been approved in the fourth-line setting and exhibited a broad activity against secondary *KIT* mutations resistant to IM (i.e., exon 13 (V654A), exon 14 (T670I), and exon 17/18 (D816V, A829P) [12,13], whereas avapritinib exhibited activity against *PDGFRA D842V* and was approved for tumors with *PDGFRA* exon 18 mutations in any line of GIST treatment [14,15,16]. Besides the mutational state of *KIT* and *PDGFRA*, the secondary resistance of GIST to IM might be due to the activation of alternative signaling pathways ongoing after the inhibition of KIT/PDGFRA signaling. In particular, this includes the overexpression of focal adhesion kinase (FAK) [17], amplification of the insulin-like growth factor receptor I (IGF-1R) [18], and activation of MET/AXL [19].

It is well known that RTK-based signaling pathways frequently overlap with each other to maintain and amplify their physiological functions. Thus, the dual inhibition of FGF/FGFR and VEGF/VEGFR pathways might be more effective in inhibiting angiogenesis and regulating the tumor’s microenvironment (discussed in detail in Section 3). The activation of FGF/FGFR pathway is well documented for GISTs and implicated in their resistance to IM [20,21]. We also showed that GISTs acquire resistance to IM due to the autocrine activation of FGFR signaling, thereby illustrating the possibilities to overcome IM resistance via the targeting of the FGFR pathway by pan-FGFR inhibitors, including BGJ 398, AZD 4547, and TAS-120 or neutralizing anti-FGF2 monoclonal antibodies [22,23,24,25].

Based on these data, illustrating that the FGF/FGFR pathway might be implicated in GIST resistance to IM and taking into account that multi-kinase inhibitors used as the second and third therapies for IM-refractory GISTs potently inhibit VEGF/VEGFR signaling, we thought to examine the potential cross-talk between FGF/FGFR and VEGF/VEGFR pathways in IM-resistant GISTs and further evaluate their anti-proliferative and cytotoxic activities when used in combination with BGJ 398, a well-known pan-FGFR inhibitor. We found that the activation of VEGF/VEGFR signaling in GISTs is a secondary event resulting from the activation of the FGF/FGFR-mediated pathway. Indeed, FGF-2 induced the autocrine activation of the FGF/FGFR pathway and also stimulated the production of VEGF-A by GIST cells, thereby activating VEGF/VEGFR signaling. Moreover, VEGFR and FGFR1/2 co-localized and co-immunoprecipitated with each other in IM-resistant GIST T-1R cells. We also demonstrated that BGJ 398 effectively potentiated the cytotoxic and anti-proliferative activities of regorafenib and sunitinib in IM-resistant GIST T-1R, but not in GIST 430 cells.

Thus, our data illustrate the functional and structural cross-talk between VEGFR and FGFR signaling pathways in the subset of IM-resistant GISTs lacking secondary *KIT* mutations and provide a rationale to re-evaluate the efficacy of the combination therapies composed of the dual inhibition of VEGFR and FGFR pathways as a late-line therapy for IM-resistant GISTs acquired resistance to the first-line targeted drug via KIT-independent mechanisms.

## 2. Results

### 2.1. FGF and VEGF Signaling Profile in IM-Resistant GISTs

**Figure 1:**
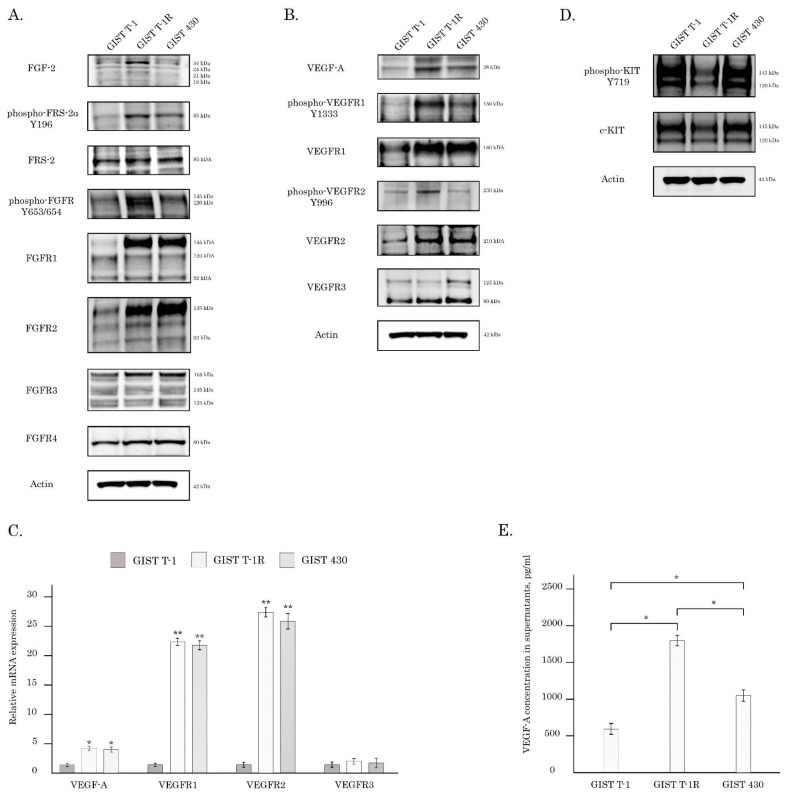
FGFR, VEGFR, and KIT signaling pathways in IM-naive (GIST T-1) and IM-resistant (GIST T-1R, GIST 430) cells. (**A**) Expression of FGF/FGFR signaling proteins in GIST cells; (**B**) expression of the VEGF/FGFR signaling proteins in GIST cells; (**C**) changes in the relative expression level of VEGF-A and VEGFR1, 2, and 3 in GIST T-1 vs. T-1R and GIST 430 cells, as determined by quantitative RT-PCR. For internal control, the amplification of glyceraldehyde-3-phosphate dehydrogenase (GAPDH) was used; data are presented as median ± SD. Significant differences with *p* < 0.05 (*), *p* < 0.01 (**) from *n* ≥ 3 using unpaired Student’s *t*-test. (**D**) Expression of the KIT signaling proteins in GIST cells. Actin stain was used as a loading control for all experiments shown in (**A**,**B**,**D**). (**E**) Concentration of VEGF-A (pg/mL) in supernatants of IM-naive (GIST T-1) and resistant (GIST T-1R and 430) cells measured by ELISA, as described in Section 4. Data are presented as median ± SD. Significant differences with *p* < 0.05 (*), *p* < 0.01 (**) from *n* ≥ 3 using unpaired Student’s *t*-test. The original Western blot figures can be found in Supplementary File S1.

First, we examined the expression of the receptors and ligands of FGFR and VEGFR signaling pathways in IM-naive and resistant GIST cells. Both of the IM-resistant GIST cell lines (i.e., GIST T-1R and 430) exhibited the increased expression of the total and phosphorylated forms of FGFR 1,2 and VEGFR 1,2 (Figure 1A,B). Additionally, GIST-T1R cells have an increase in FGF2 and VEGF-A expression, thereby suggesting the autocrine activation of the aforementioned signaling pathways (Figure 1A,B). This was in concordance with real-time PCR data, illustrating a ~3-fold increase in mRNA VEGF-A in GIST T-1R cells (Figure 1C). Importantly, we also observed a significant (~20-fold) increase in the mRNA levels of VEGFR1 and 2 in GIST T-1R when compared with parental GIST T-1 cells (Figure 1C). Similar to IM-resistant GIST T-1R cells, the increased mRNA levels of VEGF-A and VEGFR1 and 2 were observed in GIST 430 cells (Figure 1C). Of note, a significant decrease in the total and phosphorylated forms of KIT was found in IM-resistant GIST T-1R cells (Figure 1D), thereby revealing the previously shown “RTK switch” due to “KIT loss” associated with the overexpression of FGFR1/2 [22]. Additionally, the VEGF-A concentration was significantly higher in the supernatants of both IM-resistant GIST cells (GIST T-1R and GIST430 cells) when compared with IM-naive GIST T-1 cells (Figure 1E).

Based on these data illustrating the activation of VEGF/VEGFR pathway in IM-resistant GIST cell lines, we further examined their sensitivity to the corresponding RTKi (i.e., sunitinib, and regorafenib). As shown in Table 1, the IC_50_ values for VEGFR inhibitors in the GIST 430 cell line were significantly (~3- and 5-fold) higher for regorafenib and sunitinib, respectively, when compared with GIST T1-R cells, whereas the IC_50_ values of IM for both of the IM-resistant GIST cells were almost the same, thereby illustrating that different molecular pathways are able to maintain similar levels of resistance to this targeted drug.

**Figure 2:**
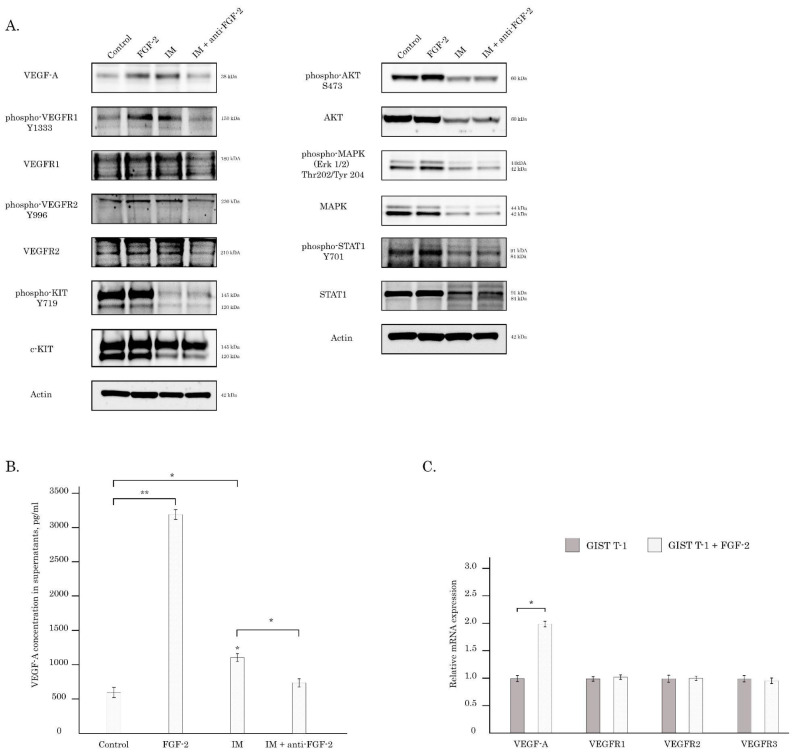
(**A**,**B**) The exogenous FGF2 activates VEGFR signaling in GIST T-1 cells via up-regulation of VEGF-A. (**A**) FGF-2 stimulates, whereas anti-FGF2 neutralizing Abs abrogates VEGFR signaling GIST T-1 cells. Cells were treated with FGF2 (100 ng/mL), IM (0.02 µM) alone or in the presence of anti-FGF2 neutralizing Abs (20 µg/mL) for 72 h. Expression of VEGF-A, total and phosphorylated forms of VEGFR, KIT, MAPK, AKT, and STAT1 was assessed by immunoblot analysis. Actin stain was used as a loading control for each sample. (**B**) Concentration of VEGF-A (pg/mL, measured by ELISA) in supernatants of IM-naive (GIST T-1) cells treated with DMSO (control), FGF-2 (100 ng/mL), IM (0.02 µM) alone or in the presence of anti-FGF-2 Abs (20 µg/mL) for 72 h. Data are presented as median ± SD. Significant differences with *p* < 0.05 (*), *p* < 0.01 (**) from *n* ≥ 3 using unpaired Student’s *t*-test. (**C**) Changes in the relative expression level of mRNA VEGF-A and VEGFR1, 2, and 3 in GIST T-1 after treatment with FGF-2 (100 ng/mL), as determined by quantitative RT-PCR. The amplification of glyceraldehyde-3-phosphate dehydrogenase (GAPDH) was used for internal control. Data are presented as median ± SD. Significant differences with *p* < 0.05 (*) from *n* ≥ 3 using unpaired Student’s *t*-test. The original Western blot figures can be found in Supplementary File S1.

Given that GIST T-1R cells exhibited the signs of autocrine activation of FGF/FGFR and VEGF/VEGFR pathways and taking into account previous reports illustrating the ability of FGF2 to activate the VEGF/VEGFR pathway [26,27], we thought to examine this possibility for IM-resistant GISTs. To address this issue directly, we initially cultured naive GIST T-1 cells with FGF2 for 72 h and further assessed the expression of VEGF-A, the total and phosphorylated forms of VEGFR1 and 2. Indeed, FGF2-treated GIST T-1 cells exhibited the increased expression of VEGF-A when compared with control cells (Figure 2A—left panel). Based on our previous data illustrating the functional connection between KIT inhibition and the activation of the FGF/FGFR pathway in GIST, we also cultured GIST T-1 cells with low concentrations of IM (0.02 µM) and also detected the increased expression of VEGF-A (Figure 2A—left panel). Similarly, the increased expression of VEGF-A was observed in GIST T-1 cells transfected by *siRNA KIT* (Supplementary Figure S1), thereby illustrating the inhibition of KIT signaling in GIST as a driving force for the activation of compensatory mechanism via the overproduction of VEGF-A and the activation of the VEGFR-mediated pathway. To examine whether this was due to the IM-induced production of FGF2 by GIST cells, we introduced the neutralizing anti-FGF2 Abs into the culture of the IM-treated GIST and observed the significant decrease in VEGF-A (Figure 2A—left panel). As expected, the increased expression of phosphorylated forms of VEGFR1 and downstream signaling molecules, including AKT, MAPK, etc., was observed for FGF2-treated GIST T-1 cells (Figure 2A—right panel), thereby illustrating the activation of VEGF signaling in a FGF2-dependent manner. In concordance with these findings, we also observed the increased expression of VEGF-A in FGF2-treated GIST cells (Figure 2B). Similarly, the elevated levels of VEGF-A were detected in the supernatants of IM-treated GIST cells, whereas presence of neutralizing anti-FGF2 Abs significantly reduced the concentration of VEGF-A, thereby revealing the FGF2-mediated mechanism of VEGF-A production (Figure 2B). This was also in concordance with real-time PCR data, illustrating the moderate (~2-fold) increase in mRNA VEGF-A in GISTs after FGF2 exposure (Figure 2C), thereby revealing the FGF2-dependent mechanism of VEGF activation in GIST. Of note, the ability of FGF2 to stimulate the production of VEGF-A was found in all GIST cell lines used in the present study. Indeed, a ~5-fold increase in VEGF-A was observed in FGF2-treated GIST T-1 cells, whereas a 2-fold increase in VEGF-A was detected in GIST T-1R cells after FGF2 exposure. A minor, albeit significant difference in the VEGF-A concentration was observed in the supernatants of FGF2-treated GIST 430 cells (Supplementary Figure S2). Thus, our data illustrate the crosstalk between FGF/FGFR and VEGF/VEGFR signaling pathways in GIST due to the ability of FGF2 to stimulate the production of VEGF-A and further activate the downstream VEGFR signaling cascade.

### 2.2. Cross-Talk between FGFR and VEGFR Signaling in IM-Resistant GISTs

**Figure 3:**
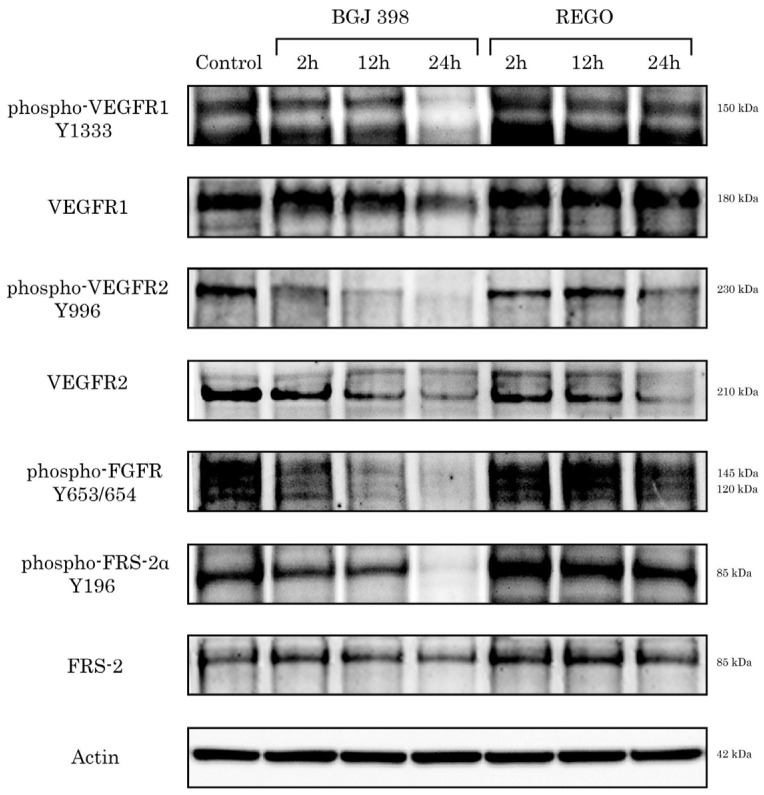
Inhibition of FGFR signaling attenuates activation of FGFR and VEGFR pathways in GIST T-1R cells. GIST T-1R cells were treated with BGJ 398 (2 µM) or regorafenib (REGO) (1 µM) for 24 h and subjected for WB analysis to examine expression of total and phosphorylated forms of VEGFR, FGFR, and FRS-2. Actin staining was used to show the comparable amounts of protein loaded into each sample. The original Western blot figures can be found in Supplementary File S1.

In concordance with these findings, we also found that the inhibition of FGF/FGFR signaling in GIST significantly attenuated the activation of the VEGF/VEGFR pathway. Indeed, besides the inhibition of the FGF/FGFR pathway in BGJ 398-treated GIST T-1R cells, we also observed the decreased expression of the phosphorylated forms of VEGFR1 and 2 (Figure 3). In contrast, regorafenib, the multi-kinase inhibitor of VEGFR signaling, inhibited the VEGFR pathway in a time-dependent manner without interfering with the activity of FGFR pathway. This was evidenced by significant decrease in expression of phosphorylated VEGFR1 and 2, whereas the expression of the phosphorylated forms of FGFR1/2 and the adaptive scaffold protein FRS-2 in regorafenib-treated GIST cells remained unchanged (Figure 3). These data suggest that the activation of the FGFR signaling pathway in IM-resistant GIST cells is a primary event further coordinating the activity of VEGF/VEGFR signaling via the autocrine-dependent mechanism due to the FGF2-induced production of VEGF-A.

**Figure 4:**
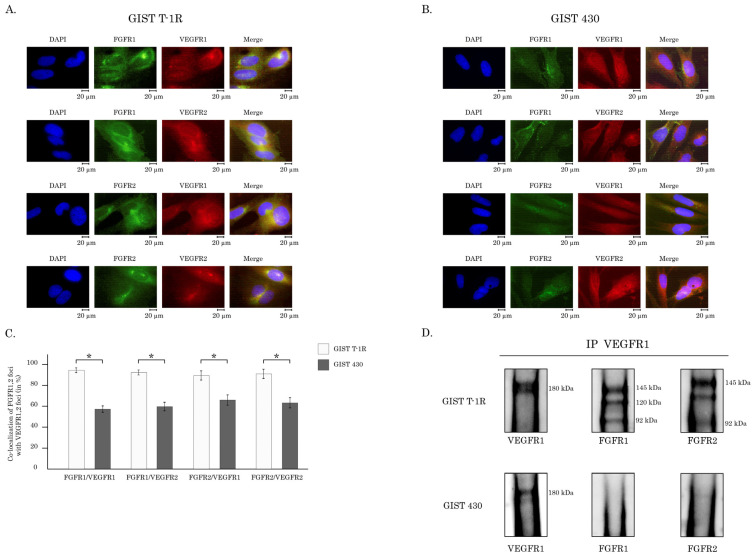
(**A**–**D**). Crosstalk between FGFR1/2 and VEGFR1/2 in IM-resistant GISTs. GIST T-1 R (**A**) and GIST 430 (**B**) were subjected to the double immunofluorescence staining for FGFR1 or 2 and VEGFR1 or 2. To outline the nucleus, the images were also merged with DAPI staining. (**C**) Percentages of cells with co-localized RTKs from three independent experiments. * *p* < 0.05; (**D**) co-immunoprecipitation of FGFR1 or 2 with VEGFR1 in IM-resistant GISTs. FGFR1 or -2 expression in GIST cell lysates immunoprecipitated by anti-VGFR1 Abs to demonstrate endogenous complex formation. The original Western blot figures can be found in Supplementary File S1.

Next, we examined the possibilities of the interactions between VEGFRs and FGFRs in IM-resistant GIST cells. For this purpose, we assessed the co-localization patterns between the aforementioned RTKs by using the immunofluorescence microscopy and also utilized co-immunoprecipitation assay to assess whether these RTKs can interact directly. Indeed, we observed a strong co-localization pattern between the aforementioned RTKs in GIST T1-R cells (Figure 4A). Similarly, FGFR2 was co-localized with VEGFR1 or 2, as shown in Figure 4A. Of note, FGFR1 and 2 were co-immunoprecipitated with VEGFR1 (Figure 4C), thereby indicating the interactions between these RTKs. Importantly, despite the high levels of expression of FGFR1, 2 and VEGFR1 observed in IM-resistant GIST 430 cells, the aforementioned RTKs were not co-localized and co-immunoprecipitated as well (Figure 4B,D, respectively), thereby suggesting that the overproduction of FGF2 and VEGF-A ligands in IM-resistant GISTs plays an important role in the complex formation between FGFRs and VEGFRs.

### 2.3. Inhibition of FGF Pathway Increases Sensitivity of IM-Resistant GIST to Anti-VEGF Therapy

**Figure 5:**
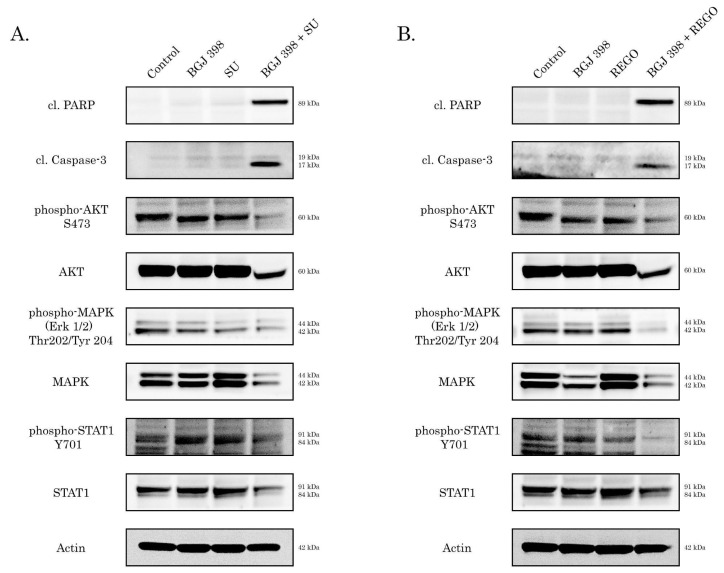
(**A**,**B**) Pro-apoptotic and anti-proliferative activities of sunitinib (SU) (**A**) or regorafenib (REGO) (**B**) used alone or in combination with BGJ 398 in GIST T-1R cells. Cells were treated for 72 h and subjected to WB analysis to examine expression of apoptotic markers—cleaved forms of PARP and caspase-3 and downstream signaling molecules of FGFR and VEGFR pathways: total and phosphorylated forms of AKT, MAPK, and STAT-1. Actin staining was used to show the comparable amounts of protein loaded into each sample. The original Western blot figures can be found in Supplementary File S1.

To examine an outcome of the crosstalk between FGF/FGFR and VEGF/VEGFR signaling pathways in IM-resistant GISTs (i.e., GIST-T1R and GIST 430 cells), we treated them by the inhibitors of VEGFR signaling (i.e., sunitinib or regorafenib) alone or in combination with BGJ 398. As shown in Figure 5A,B, both of the VEGFR inhibitors used at 0.5 µM concentration did not exhibit pro-apoptotic activity against GIST-T1R cells. Similarly, no pro-apoptotic effect was found for BGJ 398 used alone. Strikingly, we observed a substantial increase in the expression of apoptotic markers (e.g., cleaved forms of caspase-3 and PARP) in GIST-T1R treated with combination of BGJ 398 and sunitinib (Figure 5A) or regorafenib (Figure 5B).

**Figure 6:**
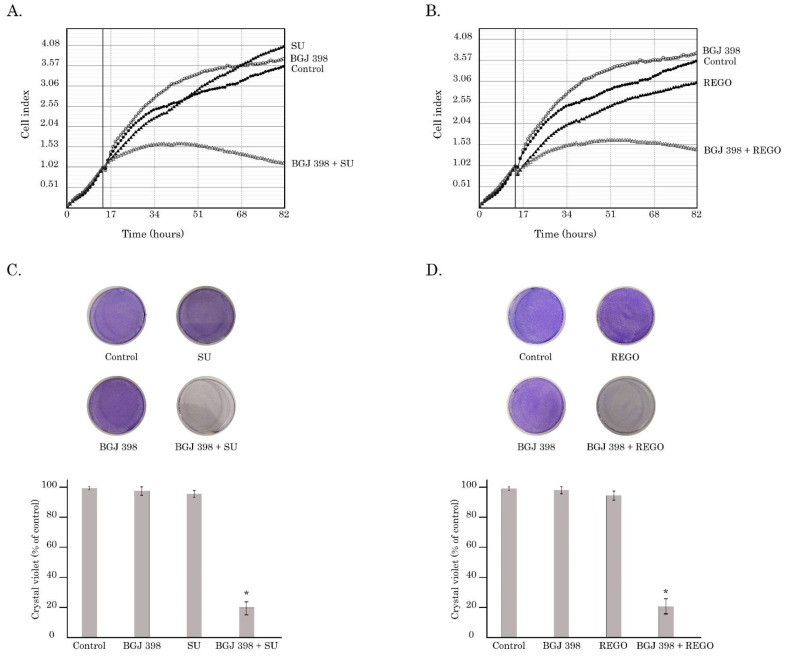
(**A**–**D**) Anti-proliferative activity BGJ 398 used in combination with sunitinib (SU) or regorafenib (REGO) in GIST-T1R cells. Cells were treated with RTKis (BGJ398 1 µM, SU 0.5µM, REGO 0.5µM) for 72 h (**A**) Changes in growth kinetics of GIST-T1R cells treated with DMSO (control), SU or BGJ 398 alone and in combination; (**B**) changes in growth kinetics of GIST-T1R cells treated with DMSO (control), REGO, or BGJ 398 alone and in combination; (**C**) (**Upper**) panel—representative images of crystal violet staining of GIST-T1R cells treated with SU or BGJ 398 alone or in combination. (**Lower**) panel—quantification of crystal violet staining of GIST cells, as shown in the upper panel. *: *p* ≤ 0.001; (**D**) (**Upper**) panel—representative images of crystal violet staining of GIST-T1R cells treated with REGO or BGJ 398 alone or in combination. (**Lower**) panel—quantification of crystal violet staining of GIST cells, as shown in the upper panel. *: *p* ≤ 0.001. The culture dishes for (**C**) and (**D**) were stained with crystal violet and photographed. The cells treated with DMSO were used as a control. Quantification of crystal violet staining of GIST cells is described in Section 4.

Similarly, sunitinib or regorafenib used in combination with BGJ 398 significantly decreased the proliferation of GIST-T1R cells, as shown in Figure 6A and Figure 6B, respectively. As expected, crystal violet staining of GIST T-1R cultures treated with the aforementioned combinations of RTKis revealed a substantial decrease in cellular viability, whereas treatment with a single RTKi have no significant impact on the GIST’s viability (Figure 6C,D).

**Figure 7:**
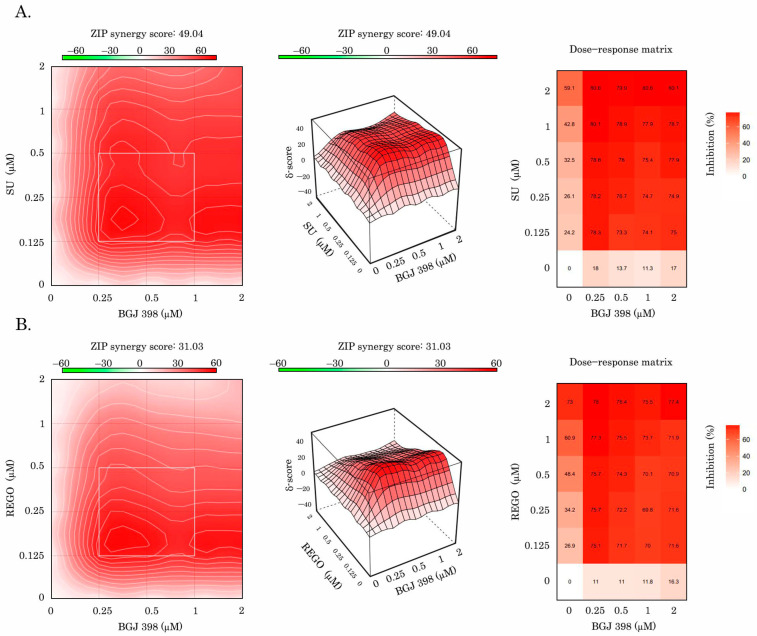
Assessment of the synergy between BGJ 398 and sunitinib (SU) (**A**) or regorafenib (REGO) (**B**) observed for IM-resistant GIST T-1R cells (ZIP model).

Lastly, for GIST T-1R cells, we observed a prominent synergy between BGJ 398 and sunitinib (Figure 7A), which was calculated by using the R-package of computational tools from SynergyFinder (https://bioconductor.org/packages/release/bioc/html/synergyfinder.html accessed on 21 June 2024) Similar results were obtained for the combination of BGJ 398 and regorafenib (Figure 7B). Importantly, a prominent synergy between BGJ 398 and VEGFR inhibitors (sunitinib and regorafenib) was observed in GIST T-1R cells by calculating the synergy scores via four different computational tools (i.e., ZIP, HSA, Bliss, or Loewe) (Table 2).

In contrast to GIST T-1R cells, GIST 430 cells were not sensitive for combination of the FGFR and VEGFR inhibitors, which was evidenced by proliferation activity and crystal violet staining (Supplementary Figure S3A–D) and absence of apoptotic markers (i.e., cleaved forms of PARP and caspase-3, as shown in Supplementary Figure S3E), no synergy or even additive effects between BGJ 398 and sunitinib or regorafenib were found for GIST 430 cells (Supplementary Figure S4 and Table 2).

To examine whether the synergy between FGFR and VEGFR inhibitors found in GIST T-1R cells was solely due to the inhibition of the aforementioned signaling pathways, we also utilized lapatinib, a well-known potent EGFR and ErbB2 inhibitor, and crizotinib (c-MET and ALK inhibitor) and used them in combination with BGJ398 or regorafenib. As shown in Supplementary Table S1, no synergy or even additive effects were found for crizotinib used in combination with FGFR or VEGFR inhibitors. Similarly, lapatininb failed to sensitize GIST T-1R cells for regorafenib, whereas a modest additive cytotoxic effect was observed in GIST T-1R cells treated with lapatinib in combination with BGJ 398. Overall, these data based on using non-targeting kinase inhibitors illustrate that potent synergy scores observed for FGFR and VEGFR inhibitors in GIST T-1R cells were solely due to the inhibition of overactivated FGFR and VEGFR signaling pathways.

Collectively, our data illustrate the high potency of dual inhibition of FGFR and VEGFR signaling in GIST that acquired resistance to IM via KIT-independent mechanisms.

## 3. Discussion

Despite the impressive response rates of IM-based therapy for patients with GIST used in the adjuvant setting and metastatic disease, primary and secondary resistance to this targeted drug remains a major challenge. Thus, second- and third-line multi-kinase inhibitors sunitinib (Sutent) and regorafenib (Strivaga), respectively, were introduced into the clinic for the patients with advanced GIST [10,11]. Sunitinib was initially identified in a drug discovery program aimed to develop small-molecule inhibitors of VEGFR and PDGFR. Due to the in vitro kinase screening assays, this drug effectively inhibited VEGFR1-3 and PDGFR-b signaling pathways via the interaction with the ATP-binding pocket of the aforementioned kinases and acting as a competitive inhibitor with ATP [28]. Sunitinib effectively inhibited the VEGF- or PDGF-mediated phosphorylation of VEGFR-2 and PDGFR-b, respectively, in NIH-3T3 cells (IC_50_ 0.01 µM) [28], stem-cell factor (SCF)-induced phosphorylation of KIT in NCI-H526 human small cell lung cancer (SCLC) cells (IC_50_ 0.001–0.01 µM) [29], FLT ligand-mediated phosphorylation of FLT3 expressed in RS4;11 and OSAML5 acute myeloid leukemia (AML) cells (IC_50_ 0.25 µM) [30], and M-CSF-dependent phosphorylation in NIH-3T3 cells expressing CSF-1R (IC_50_ 0.05–0.1 µM) [31]. Additionally, wild-type RTKs sunitinib also exhibited a potent inhibitory capacity against mutated RTKs with constitutive kinase activities, including mutant forms of *KIT* or *PDGFR-a* playing a key regulatory role in GIST pathogenesis [32,33]. In addition to the inhibitory activity of sunitinib against constitutively active *KIT* exon 9 and exon 11 mutants commonly found in naive GIST cells [34], this RTKi also potently inhibited exons 13 and 14 mutant variants of *KIT* (i.e., V654A and T670I), which are known to be associated with reduced binding affinity and resistance to IM [35,36,37]. Based on these findings, sunitinib was approved for the treatment of patients with IM refractory or intolerant GISTs on January 26, 2006 and is currently used as a second-line therapy for this disease. However, clinical progression on sunitinib occurs after a median time of 6 months or less, thereby highlighting about heterogeneity of IM resistance mechanisms. Indeed, in vitro and clinical studies demonstrated that sunitinib exhibited potent inhibitory activity against *KIT* ATP-binding pocket V654A secondary mutations in IM-resistant GISTs. However, this RTKi was not effective against GIST subclones with *KIT* activation loop mutations [38,39]. Thus, the clinical progression of GISTs after the initiation of sunitinib-based therapy is mainly due to the emergence of cross-resistant KIT-dependent GIST subclones [40,41,42] Likewise, the polyclonal heterogeneity of IM-resistant GIST subclones, including those that acquired IM resistance, due to the non-KIT-driven mechanisms might lead to the benefits observed with regorafenib used as a third-line therapy for GISTs and other TKIs used as a single-agent therapies or in combination.

The activation of FGF/FGFR and VEGF/VEGFR signaling pathways was evidenced for a broad spectrum of human malignancies and playing an important regulatory role in carcinogenesis, tumor development, and progression [43]. We showed previously that GISTs lacking secondary *KIT* mutations might acquire resistance to IM due to the activation of FGFR signaling [22]. We also observed that BGJ 398, a pan-FGFR inhibitor, effectively restored GISTs’ sensitivity to IM, thereby providing a rationale for the combined therapies to inhibit the activities of both KIT and FGFR signaling pathways. This approach seems to be effective for GIST patients who exhibited the activation of FGFR signaling and progressed over the first-line therapy [23]. We also found that IM activated FGF/FGFR signaling in GISTs via the autocrine mechanism due to the overproduction of FGF2. This was shown for IM-treated GIST cell lines, GIST xenografts, and clinical specimens as well [24]. Of note, the presence of neutralizing anti-FGF2 Abs in GIST cultures reversed their sensitivity to IM [24]. Thus, we concluded that KIT inhibition in IM-resistant GISTs activates FGFR signaling and might have an impact on the malignant behavior of IM-resistant tumors and disease progression. Similarly, VEGF expression was previously proposed as a predictive factor for early treatment failure and poor survival of GIST patients on IM-based therapy, independent of *KIT* genotype [44].

We report here about the activation of the VEGFR pathway in IM-resistant GIST T-1 subline previously established in our laboratory. This was evidenced by an increased expression of VEGF-A and the total/phosphorylated forms of VEGFR1, 2 for this particular GIST subline and other IM-resistant GIST cells, including GIST 430 (Figure 1). Importantly, exogenous FGF-2 induced extensive phosphorylation of VEGFR1 in naive GIST T-1 cells (Figure 2A). Of note, a similar pattern was observed for GIST-T1 cells treated with low (0.02 µM) concentrations of IM, whereas presence of anti-FGF2 mAbs abrogated activation of VEGFR signaling in KIT-inhibited GIST (Figure 2). This was in contrast with previous reports illustrating IM’s potency to inhibit both the transcription and translation of VEGF in GIST T-1 and GIST882 cells [45,46]. The opposing effects of IM on VEGF production by GIST cells in vitro might be due to IM used in 1 µM of for these studies [44,45], therefore the down-regulation of VEGF production in IM-treated GIST were due to the decreased viability, which was evidenced by the MTT-based assay [45]. Of note, based on MTS-based cytotoxicity data, as shown in Table 1, we chose the subtoxic concentration of IM (0.02 µM) for IM-naive GIST T-1 cells to examine whether the inhibition of KIT signaling in GIST affects the production of VEGF-A regardless of the cellular viability. Strikingly, IM used at 0.02 µM was still sufficient enough to effectively inhibit KIT signaling in GIST T-1 cells (Figure 2), which was accomplished with the increased production of VEGF-A, thereby illustrating functional connection between the KIT inhibition and activation of VEGFR signaling in IM-naive GIST T-1 cells. Importantly, FGF-2 used for IM-resistant GISTs (i.e., GIST T-1R and GIST 430) also significantly increased the production of VEGF-A (Supplementary Figure S1), thereby illustrating that FGF2-mediated signaling in GIST might activate the compensatory mechanism of VEGFR activation to maintain cancer cell survival and angiogenesis in GISTs, thereby illustrating that the inhibition of KIT signaling in GISTs might the activate compensatory mechanism of VEGFR activation to maintain cancer cell survival and angiogenesis in GISTs. This is consistent with in vivo studies illustrating that VEGF expression in GISTs might be a predictive factor for early treatment failure and poor survival of GIST patients on IM-based therapy, regardless of *KIT* genotype [44,46]. Of note, IM might variably affect VEGF expression in GISTs and patients that continued expressing VEGF after neoadjuvant IM have experienced a progression of the disease [44]. Thus, patients whose GIST produces VEGF may benefit from the anti-VEGFR therapy.

Thus, our data illustrate the functional connection between FGFR and VEGFR pathways in GISTs and highlight the regulatory role of FGF2 in VEGFR-mediated signaling. This is consistent with the reports illustrating a crosstalk between FGFR- and VEGFR-mediated pathways in human malignancies and the regulatory role of the FGFR pathway in VEGFR signaling. In particular, Golfmann K., with co-authors, demonstrated that FGF-FGFR1 signaling regulates VEGF secretion via breast cancer cells and activates the VEGF-VEGFR1 pathway [47]. This is in agreement with reports illustrating the bidirectional crosstalk between FGF2 and VEGF. Indeed, FGF2 was shown to promote tumor angiogenesis via the up-regulation of VEGFR and VEGF-A, whereas VEGF, in turn, up-regulates FGF expression. FGF can also induce the expression of VEGFR2 a in ERK1/2-dependent manner and VEGFR2 expression declines without this interaction [26,27,48]. Thus, it is not surprising that FGFR and VEGFR signaling pathways are providing the synergistic angiogenic effects. Indeed, by using the quantitative fibrin-based 3-dimensional angiogenesis system in vitro, Xue L., with co-authors, observed that the combination of FGF-1 and VEGF exhibited a synergistic angiogenic effect, whereas neutralizing anti-VEGF mAbs reduced FGF-driven angiogenesis [49]. Similarly, the up-regulation of FGF on the pericytes was recently shown as a potent mechanism underlying in resistance to anti-VEGF/VEGFR therapy [50]. On the other side, VEGF-B might prevent excessive angiogenesis by inhibiting the FGF2/FGFR1-mediated pathway. Mechanistically, VEGF-B was able to bind with FGFR1 and further induce the formation of FGFR1/VEGFR1 complex. This, in turn, effectively inhibited FGF2-induced Erk activation and FGF2-driven angiogenesis [51]. Besides of the effective controlling of tumors angiogenesis, both of VEGF-VEGFR and FGF-FGFR signaling pathways are currently known as potent regulators of tumor immunity via the modulation of the tumor microenvironment (TME) by inducing macrophage polarization [52] and enhancing the infiltration and activity of cytotoxic T lymphocytes, mature dendritic cells, and NK cells [53,54,55]. The dual inhibition of VEG/VEGFR and FGF/FGFR signaling can also trigger strong anti-tumor immunity by interfering with immune checkpoints, including PD-1 and PDL-1 [56,57].

We show here that besides the well-known anti-angiogenic effects resulting from dual FGFR and VEGFR inhibition, the simultaneous inhibition of the aforementioned RTKs in IM-resistant GISTs potentiates their cytotoxic and anti-proliferative activities. Indeed, increased expression of apoptosis markers (i.e., cleaved forms of PARP and caspase-3) was observed in IM-resistant GIST T-1R cells treated with a combination of BGJ 398 (pan-FGFR inhibitor) and VEGFR inhibitors (sunitinib and regorafenib) used at subtoxic (0.5 µM) concentrations (Figure 5). Importantly, FGFR or VEGFR inhibitors used alone were not effective (Figure 5). Similarly, a combination of FGFR and VEGFR inhibitors potently decreased proliferation of IM-resistant GIST T-1 cells, as shown in Figure 6.

Besides the functional connection between FGFR and VEGFR in IM-resistant GISTs, we observed a strong co-localization pattern and direct interaction between the aforementioned RTKs (Figure 4A,C,D). The close proximity of the aforementioned RTKs or their physical interaction might be responsible for the decreased activation of VEGFR signaling in FGFR-inhibited cells and explain the impressive synergy score rates observed between FGFR and VEGFR inhibitors in IM-resistant GIST T1-R cells (Figure 7, Table 2). Our data is consistent with multiple reports illustrating the possibilities for interaction between different members of RTK family in cancer cells. Indeed, while RTKs and their downstream cascades have been often studied separately, it is becoming clear now that different members of the RTK family can interact with each other. For example, FGFR and EGFR are not thought to oligomerize on the plasma membrane, and they bind to and become activated by different ligands—FGF and EGF, respectively [58,59]. However, this point of view was recently re-evaluated. Indeed, by using high-resolution and high-throughput fluorescence imaging, Alfonzo-Méndez M, with co-authors, found that stimulation with EGF induces a capture and concentration of EGFR, FGFR, and low-density lipoprotein receptors (LDLR) at clathrin-coated structures across the plasma membrane of human squamous HSC3 cells. Of note, several regulatory proteins, including ubiquitin ligase Cbl and the scaffold Grb2, were also recruited. Importantly, the disruption of FGFR or EGFR by the corresponding inhibitors (PD-166866 and gefitinib, respectively) prevented the recruitment of both EGFR and FGFR to the clathrin-coated structures of the plasma membrane [60]. Thus, it is not surprising that EGFR inhibitors improved clinical outcomes in cancers driven by the activation of FGFR signaling [61]. Lee C., with co-authors, discovered the anti-angiogenic nature of VEGF-B, which binds to FGFR1 and induces the formation of the FGFR1/VEGFR1 complex. As an outcome of this interaction, VEGF-B effectively inhibited FGF2-driven angiogenesis and tumor growth [51]. Moreover, signaling molecules and adaptor and effector proteins of VEGFR and FGFR pathways acting downstream of RTKs (i.e., FRS2α, Grb2, AKT, MAPK, STAT-1, -3, S6, etc.) might also be responsible for the synergy between FGFR and VEGFR inhibitors. For example, FRS2α, a well-known scaffold protein of FGFR1, was shown to be a regulator of the VEGF-A- and VEGF-C-mediated activation of the extracellular signal-regulated receptor kinase signaling and blood and lymphatic endothelial cell migration and proliferation. Moreover, the deletion of *FRS2α* profoundly reduced VEGF signaling in HUVECs in vitro, which resulted in their decreased proliferation, migration, and matrigel cord formation. Importantly, the deletion of *FRS2α* impaired postnatal vascular development. Similarly, the deletion of *FRS2α* resulted in the impairment of adult angiogenesis and lymphangiogenesis, thereby illustrating the regulatory role of this FGFR adaptor protein in the VEGFR signaling pathway [62]. Mechanistically, FRS-2, the scaffold protein of FGF signaling and the interaction with the growth factor receptor-binding protein 2 (GRB2) might interfere with its functional activity, thereby affecting the activity of the downstream cascades of both VEGFR and FGFR pathways.

In concordance with these data, we observed the impairment of VEGFR signaling in FGFR-inhibited GIST cells. As shown in Figure 3, BGJ 398, a pan-FGFR inhibitor, effectively down-regulated VEGFR signaling in GIST T-1R cells. This was in agreement with our data illustrating that, in the presence of anti-FGF2 monoclonal Abs, KIT-inhibited GIST cells failed to produce VEGF-A and activate the VEGFR signaling cascade (Figure 1), thereby highlighting the pivotal regulatory role of the FGFR pathway in VEGFR signaling in GIST T-1R cells. In contrast, a similar RTKi used alone or in combination was not effective in IM-resistant GIST 430 cells (Supplementary Figures S2 and S3 and Table 2). This might be due to the differences in the RTK profile and *KIT* mutational status between these IM-resistant GIST cell lines. As shown in Figure 1, GIST T-1R cells exhibited the signs of excessive activation in FGFR (i.e., FGF-2, pFRS-2, pFGFR1/2) and VEGFR (VEGF-A, pVEGFR1 and 2) pathways, thereby rendering them sensitive to dual FGFR and VEGFR inhibition. Moreover, the expression of the phosphorylated and total KIT was significantly lower in GIST T-1R cells when compared with GIST 430 cells (Figure 1). These data illustrate the impaired role of KIT signaling in the maintenance of GIST T-1R’s survival and proliferation, which was previously named as the “RTK switch” [22]. Moreover, the activation of VEGF/VEGFR and FGF/FGFR pathways in GIST T-1R cells (associated with significant decrease in KIT expression and the absence of secondary *KIT* mutations) renders them highly sensitive to the inhibition of the aforementioned signaling cascades by the corresponding RTKi. In contrast, GIST 430 cells exhibiting primary *KIT* exon 11 deletion (V560_L576del) and secondary *KIT* exon 13 mutation (V654A) maintain survival and IM resistance mainly via KIT-dependent mechanisms, thereby explaining the lack of synergy between FGFR and VEGFR inhibitors for this particular IM-resistant GIST cell line.

**Figure 8:**
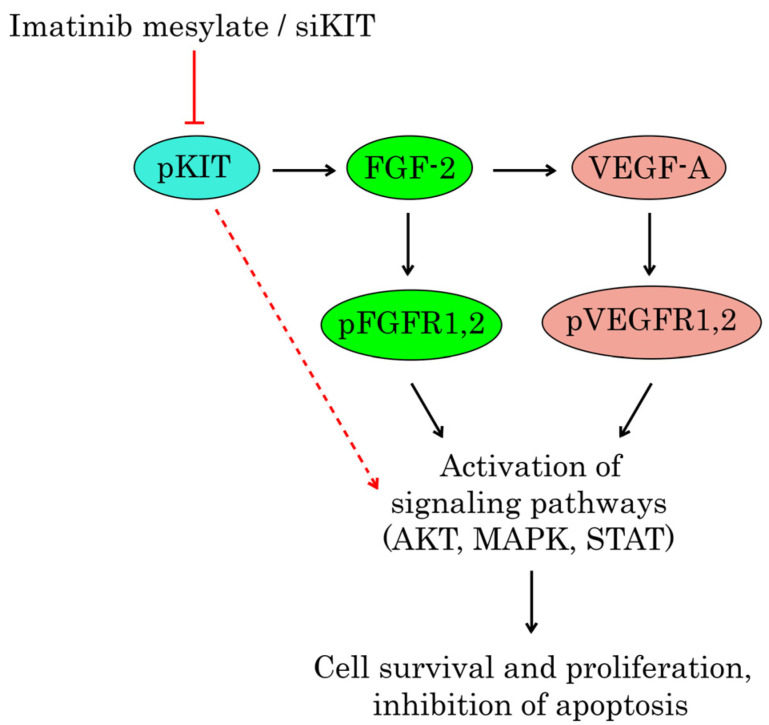
Inhibition of KIT signaling activates FGFR and VEGFR signaling via overproduction of FGF-2 and VEGF-A in IM-resistant GIST-T1 cells.

Collectively, the inhibition of KIT signaling in GIST leads to the “paradoxal” activation of the VEGFR pathway via the autocrine production of FGF2, as shown in Figure 8. Given that both FGF/FGFR and VEGF/VEGFR pathways are known as potent regulators of angiogenesis, the dual targeting of the aforementioned pathways might be a promising therapeutic approach for IM-resistant GISTs, resulting in synergistic anti-angiogenic treatment effects. Besides the anti-angiogenic effect, we also demonstrated here that the dual inhibition of FGFR and VEGFR signaling potentiated proapoptotic and antiproliferative activities in GIST cells (Figure 5 and Figure 6, respectively) and resulted in the impressive synergy scores between BGJ 398 and sunitinib or regorafenib (Figure 7). Thus, this therapeutic approach might be effective for GISTs with acquired resistance to IM due to the “RTK switch” resulting from the down-regulation of KIT and the compensatory activation of FGFR and VEGFR signaling pathways. In contrast, IM-resistant GIST430 cells that acquired resistance to IM via KIT-driven mechanisms exhibited low sensitivity to the aforementioned FGFR and VEGFR inhibitors used alone or even in combination (Supplementary Figures S2 and S3), thereby indicating that combined anti-FGFR and VEGFR therapies might be highly effective for IM-resistant GIST-acquired resistance to IM via KIT-independent mechanisms.

The attractiveness of the inhibition of VEGFR signaling for GIST therapy is evidenced by the multiple completed and ongoing clinical trials (reviewed in detail by Catalano F. with co-authors) [63]. Besides sunitinib and regorafenib being accepted as the second- and third-line therapies for advanced GIST, cabozatinib and the multiple RTKi, including VEGFR2, can also be considered as a further treatment line for GIST. The results of the CaboGIST EORTC trial demonstrated an encouraging disease control rate of 82% and a median progression-free survival of 5.5 months. Moreover, >50% of patients enrolled in this study were progression-free after 12 weeks [64]. The effectiveness of Lenvatinib, a potent VEGFR inhibitor, is currently studied for unresectable advanced GISTs after the failure of standard treatments (imatinib, sunitinib) in phase 2 of the LENVAGIST clinical trial [65]. Apatinib, a potent VEGFR2 inhibitor, is currently being examined in a randomized single-center clinical trial [66] as a standard second-line therapy TKI for advanced GISTs, whereas the effectiveness of regorafenib for metastatic and/or unresectable previously untreated wild-type GISTs is currently being assessed in the REGISTRI phase 2 clinical trial [67]. The rationale of a combination therapy targeting FGF and VEGF signaling pathways was shown as a viable therapeutic option for several malignancies outside of GISTs. In particular, anlotinib (AL3818) is a multi-TKI targeting VEGFR1-3, FGFR1-4, PDGFRα/β, and c-Kit and Ret was approved as a third-line or beyond therapy for stage IV NSCLC. Importantly, the tolerability profile of anlotinib was similar to that of other tyrosine kinase inhibitors targeting VEGFR, whereas the incidence of grade 3 or higher side effects was much lower than that of other TKIs [68]. Besides NSCLC, anlotinib also demonstrated promising efficacy in patients with metastatic renal cell carcinoma (RCC), refractory metastatic soft-tissue sarcomas (STS) progressed after anthracycline-based chemotherapy, and advanced or metastatic medullary thyroid carcinoma [69,70,71].

## 4. Materials and Methods

### 4.1. Chemical Compounds

Imatinib mesylate (IM), infigratinib (BGJ 398), sunitinib (SU), and regorafenib (REGO) were obtained from SelleckChem (Houston, TX, USA).

### 4.2. Antibodies

Primary antibodies used for immunoblotting and immunofluorescence were as follows: phospho-MAPK (Erk1/2) Thr202/Tyr204 (#4370S), MAPK (Erk1/2) (#4696S), phospho-KIT Y719 (#3391S), phospho-AKT S473 (#4060P), AKT (#4691p), phospho-FRS2α Y196 (#3864S) and Y436 (#3861S), phospho-FGFR Y653/654 (#3476S), FGFR1 (#9740S), FGFR2 (#23328S), cleaved form of caspase-3 (#9662S), VEGFR1 (#64094S), phospho-VEGFR2 (#2474S) (Cell Signaling, Danvers, MA, USA), KIT (#A4502, Dako, Carpinteria, CA, USA), FGF-2 (sc-365106), VEGFR1 (sc-271789) (Santa Cruz Biotechnology, Santa Cruz, CA, USA), beta-actin (A00730-200, GenScript, Piscataway, NJ, USA), VEGFR2 (ab39378), VEGFR2 (ab134191), VEGFR3 (ab243232) (Abcam, Cambridge, UK), phospho-VEGFR1 (SAB4504006), and VEGF-A (ZRB1 320) (Sigma-Aldrich, St-Louis, MO, USA). HRP-conjugated secondary antibodies for Western blotting were purchased from Santa Cruz. Neutralizing antibody against bFGF (Anti-FGF2/basic FGF #05-117) and human recombinant FGF-2/basic (FGF2 #01-106) were from Merck KGaA (Darmstadt, Germany).

### 4.3. Cell Lines and Culture Conditions

GIST T-1 cell line was derived from a metastatic pleural tumor from stomach GIST and contains heterozygous 57-base pair deletion (V570-Y578) in *KIT* exon 11 [72]. IM-resistant GIST T-1R subline was generated from GIST T-1 parental cells, as was shown elsewhere [22]. IM-resistant GIST 430 cell line was established from a GIST that developed clinical resistance to IM. This cell line contained a heterozygous primary *KIT* exon 11 deletion (V560_L576del) and a secondary *KIT* exon 13 point mutation (V654A) [73]. Aforementioned GIST cell lines were grown in complete RPMI-1640 or DMEM/F-12 culture medium supplemented with antibiotics (Paneko, Moscow, Russia). Cells were maintained in humidified atmosphere of 5% CO_2_ at 37 °C (LamSystems, Myass, Russia).

### 4.4. Cellular Survival MTS-Based Assay

To examine the cytotoxic activities of the targeted drugs (i.e., IM, SU, and REGO), GIST cells (0.5 × 10^5^/mL) were seeded into flat-bottomed 96-well plates (Corning Inc., Corning, New York, NY, USA) and were allowed to attach and grow for the next 24 h. Next, the cells were cultured for 72 h in the presence of various concentrations of targeted drugs (from 400 µM to 0.000001 µM) or DMSO (control). Subsequently, the culture medium was removed and replaced by the medium containing MTS (Promega, Madison, WI, USA) and Phenazine methosulfate (PMS) (Sigma-Aldrich, St-Louis, MO, USA) at a ratio of 20:1. Cells were incubated with MTS/PFS for 1 h. Cellular viability was analyzed at 492 nm on a MultiScan FC plate reader (Thermo Fisher Scientific, Waltham, MA, USA). Half-dose inhibitory drug values (IC50) were defined as the concentration of a compound required to inhibit cell growth by 50% in 72 h. Data were normalized to the control cells treated with DMSO. IC_50_ values were determined by using the IC_50_ Tool Kit (http://ic50.tk/, accessed on 23 July 2024).

The potential additive, the antagonistic and synergistic effect of BGJ 398 on sunitinib, regorafinib, or their combinations, were calculated by using the R-package of the computational tool SynergyFinder (https://bioconductor.org/packages/release/bioc/html/synergyfinder.html- accessed on 21 June 2024). For this purpose, 4 types of models were used: Highest single agent (HSA) model [74], Loewe additivity model [75], Bliss independence model [76] and zero interaction potency (ZIP) model [73]. A value of synergy score (SC) of BGJ 398 and RTKi (sunitinib/regorafinib) combinations < −10 was considered an antagonistic effect, whereas SC between −10 and 10 and SC > 10 was considered an additive and synergistic effect, respectively [77].

### 4.5. Real-Time Monitoring of Cell Proliferation

GIST cells (0.5 × 10^5^/mL) were seeded into the wells of an E-Plate L8 PET cassette (ACEA Biosciences, San Diego, CA, USA). The cassettes were installed in the iCELLigence cell growth kinetics system (ACEA Biosciences, San Diego, CA, USA). Cells were allowed to attach and grow for the following 24 h. Subsequently, BGJ 398 1 μM, SU 0.5–1 μM, REGO 0.5–1 μM, alone or in combination, were introduced in the cell culture. DMSO-treated cells were used as a negative control. Cell proliferation index values were recorded every hour throughout the experiment. RTCA Software version 1.0 (ACEA Biosciences, Inc., San Diego, CA, USA) was used to analyze the data.

### 4.6. Crystal Violet Staining

For staining, a crystal violet fixing solution of the following composition was used (per 50 mL dH_2_O): 25 mg crystal violet; 3.1 mL of 16% formaldehyde solution; 5 mL of PBS (10×); and 0.5 mL of 100% methanol. The medium was removed from the culture dishes and crystal violet fixative solution was added (3 mL solution for dish p60, 6 mL for dish p100). Incubate for 20 min at room temperature in the dark. Next, the dishes were washed from the fixing solution in a container with cool water for 2–3 min and left to dry on filter paper for 2–3 h at room temperature. Afterwards the dishes were photographed. To quantify crystal violet staining, 1% SDS solution was added to the dishes (1.5 mL solution for dish p60, 3 mL for dish p100) and incubated for 1 h at room temperature on a shaker. Next, 100 μL of the solution from the dishes was added to the wells of a 96-well plate and the optical density was measured at 540 nm on a MultiScan FC plate reader (Thermo Fisher Scientific, Waltham, MA, USA).

### 4.7. Western Blotting and Co-Immunoprecipitation (Co-IP)

For Western blotting, GIST lysates were prepared on ice (20 min) using RIPA buffer (25 mM Tris-HCl, pH 7.6, 150 mM NaCl, 5 mM EDTA, 1% NP-40, 1% sodium deoxycholate, 0 <1% SDS) supplemented with a protease inhibitor cocktail solution and PMSF (Sigma-Aldrich, St-Louis, MO, USA). To obtain the supernatants of whole cell lysates (WCL) the samples were centrifuged for 30 min at 13,000 rpm at +4 °C. A mixture of LDS sample buffer (Invitrogen, Carlsbad, CA, USA) and 2-mercaptoethanol (Sigma-Aldrich, St-Louis, MO, USA) was added to samples containing 20–30 μg of protein. After heating (+95 °C for 5 min) and subsequent cooling to +4 °C, samples were loaded into wells of a NuPAGE gel containing 4 to 12% bis-Tris or 3 to 8% Tris-acetate (Invitrogen, Carlsbad, CA, USA) and further subjected to vertical electrophoresis at 80 V. Subsequently, a protein transfer procedure was performed onto a nitrocellulose membrane (Bio-Rad, Hercules, CA, USA). Next, the nitrocellulose membrane was blocked in a solution containing non-fat dry milk or BSA. After this, the nitrocellulose membrane was incubated with the appropriate primary Abs overnight at +4 °C, washed out with PBS and incubated with secondary Abs (Bio-Rad, Hercules, CA, USA) for 1 h at room temperature. Detection of the nitrocellulose membrane was carried out using chemiluminescent solutions Clarity Western ECL substrate or Clarity Max Western ECL Substrate (Bio-Rad, Hercules, CA, USA). Protein expression was assessed in chemiluminescence mode in gel documentation using the Fusion Solo S system (Vilber Lourmat, France). Densitometry of Western blot images was performed using NIH Image J software (version 1.49)(Bethesda, MD, USA).

For Co-IP, whole cell lysates (WCL) were prepared on ice (10 min) using TEB buffer (50 mM Tris-HCl pH 7.5, 150 mM NaCl, 1% NP-40, 10% glycerol) supplemented with a protease inhibitor cocktail solution and PMSF (Sigma-Aldrich, St-Louis, MO, USA). The samples were then centrifuged for 10 min at 13,000 rpm at +4 °C to obtain the supernatants (whole cell lysates). VEGFR1 precipitating antibodies (#64094S, Cell Signaling, Danvers, MA, USA), at a concentration of 2 μg, were added to WCL and incubated overnight at +4 °C on a shake and further loaded by 30 μL of protein A and G Sepharose beads (Santa Cruz Biotechnology, Santa Cruz, CA, USA) and incubated on a shaker for 1 h at +4 °C. After washing with TEB buffer, a mixture of LDS sample buffer (Invitrogen, Carlsbad, CA, USA) and 2-mercaptoethanol (Sigma-Aldrich, St-Louis, MO, USA) was added to the samples. The samples were further subjected to vertical electrophoresis and blotting, as shown before.

### 4.8. Immunofluorescence Staining

GIST cells were seeded into the 6-well flat-bottom plates (Corning Inc., Corning, NY, USA) containing Poly-L-lysine-coated coverslips (Sigma-Aldrich, St. Louis, MO, USA). Over the next 72 h, the cells were allowed to attach and grow. The cells were further fixed with a 4% paraformaldehyde solution (in PBS) for 15 min at room temperature. For FGFR2 staining, cells were permeabilized with an ice-cold solution of 100% methanol (10 min at a temperature of −20 °C). After washing with PBS, the glass coverslips were then incubated in a blocking solution (1× PBS, 5% goat serum, 0.3% Triton X-100) for 1 h at room temperature. After this, the cells were incubated with primary antibodies dissolved in antibody dilution buffer (1× PBS, 1% BSA, 0.3% Triton X-100) overnight at 4 °C. Next day the cells were washed with PBS (three times for 5 min each in the dark), incubated with Alexa Fluor 488, or TexRed-conjugated secondary antibodies (Invitrogen, Carlsbad, CA, USA) dissolved in antibody dilution buffer (1× PBS, 1% BSA, 0.3% Triton X-100) for 1 h at room temperature in the dark. Next, the cells were washed with PBS (three times for 5 min each in the dark). Next, the cells were stained with DAPI solution (Sigma-Aldrich, St. Louis, MO, USA) for 30 s. After washing with PBS the coverslips were placed on the glass slides and cells were visualized using an Olympus BX63 fluorescence microscope (Tokyo, Japan). Images were acquired using the Spot Advanced Imaging System.

### 4.9. KIT Gene Silencing Using siRNA

For KIT gene knockout, On-Target Plus Smartpool siRNA human KIT (# L-003150-00-005, Dharmacon RNA Technologies, Lafayette, CO, USA) and Lipofectamine RNAimax Reagent (# 1928036, Invitrogen, Carlsbad, CA, USA) were used. These reagents were dissolved in Opti-MEM culture medium (Thermo Fisher Scientific, Waltham, MA, USA). The final siRNA concentration was 2.5 nM oligonucleotides. Cells transfected with non-target control siRNA were used as a negative control. Transfected GIST T-1 cells were cultured in RPMI-1640 culture medium (Paneko, Moscow, Russia) for 48 h. Next, WCL were prepared as shown above and analyzed by immunoblotting.

### 4.10. RNA Extraction and Real-Time Quantitative PCR

Total RNA was extracted from GIST cells according to the protocol shown previously [22]. Next, 1 µL template cDNA was used in a real-time qPCR reaction with 5х qPCRmix-HS SYBR (PB025, Evrogen, Moscow, Russia), and 10 mM of forward and reverse primers, as shown in Supplementary Table S2, was introduced in the mixture. Real-time qPCR was carried out using the CFX96 Real-Time detection system (Bio-Rad, Hercules, CA, USA), according to the manufacturer’s protocol. GAPDH was used for internal normalization control. Generation of quantitative data was based on the number of cycles required for the amplification-generated fluorescence to reach a specific threshold of detection (the Ct value).

### 4.11. Enzyme-Linked Immunosorbent Assay

The levels of VEGF-A in supernatants of GIST cells were measured using human VEGF-A ELISA Kit (RAB0507, Sigma-Aldrich, St-Louis, MO, USA), according to the manufacturer’s instructions. The plates were read at 450 nm on MultiScan FC plate reader (Thermo Fisher Scientific, Waltham, MA, USA).

### 4.12. Statistics

All the experiments were repeated a minimum of 3 times. The results are presented as the mean ± standard deviation (SD) for each group. Differences were considered significant at *p* < 0.05.

## 5. Conclusions

Activation of VEGF/VEGFR signaling in IM-resistant GIST cells might be a result of FGF2 production by KIT-inhibited cancer cells, which in turn leads to the production and secretion of VEGF-A, and thereby activates VEGFR-mediated cascade. This in turn renders IM-resistant GIST highly sensitive to dual targeting of FGFR and VEGFR pathways and potentiates the pro-apoptotic and anti-proliferative activities of the corresponding RTKi. Thus, our data uncovers the novel mechanism of the cross-talk between FGFR- and VEGFR- mediated cascades in IM-resistant GISTs lacking secondary KIT mutations and suggests that the dual blockade of the aforementioned signaling pathways might be an effective treatment strategy for patients with GIST that acquired resistance to IM via KIT-independent mechanisms.

## Figures and Tables

**Table 1:** Comparative IC_50_ values (µM) for IM, sunitinib (SU), and regorafenib (REGO) in GIST-T1, T1R and 430 cell lines.

Cell Line	GIST T-1	GIST T-1R	GIST 430
IM	0.04 ± 0.0003	47.1 ± 1.3	54.3 ± 7.9
Fold increase relative to GIST T-1		1177.5	1357.5
Fold increase relative to GIST T-1R			1.15
SU	0.016 ± 0.0001	5.2 ± 0.5	15.3 ± 2.4
Fold increase relative to GIST T-1		325.0	956.2
Fold increase relative to GIST T-1R			2.94
REGO	0.029 ± 0.003	2.2 ± 0.3	14.8 ± 1.9
Fold increase relative to GIST T-1		76.0	510.4
Fold increase relative to GIST T-1R			6.7

Fibroblast growth factor-2 (FGF-2) induces vascular endothelial growth factor (VEGF) expression in IM-resistant GISTs.

**Table 2:** Synergy scores between BGJ 398 and sunitinib (SU) or regorafenib (REGO) observed for IM-resistant GIST T-1R and GIST 430 cells.

Cell Line	BGJ 398 + RTKi	ZIP	Bliss	Loewe	HSA
GIST T-1R	SU	49.04	49.05	34.45	49.69
	REGO	31.03	31.00	10.98	31.07
GIST 430	SU	−38.21	−39.24	−6.11	−34.82
	REGO	−29.77	−30.59	−8.64	−21.14

## Data Availability

The original contributions presented in the study are included in the article/Supplementary Materials; further inquiries can be directed to the corresponding author.

## Supplementary Materials

The following supporting information can be downloaded at https://www.mdpi.com/article/10.3390/cancers16173103/s1, File S1: The original Western blot figures; Figure S1: Activation of VEGFR signaling in GIST T-1 cells upon knockout of the *KIT* gene; Figure S2: Exogenous FGF2 increases VEGF production by IM-sensitive (GIST T-1) and resistant (GIST T-1R and GIST 430) cells; Figure S3: Pro-apoptotic and anti-proliferative activities of BGJ 398 used in combination with sunitinib (SU) or regorefenib (REGO) in GIST 430 cells; Figure S4: Assessment of the synergy between BGJ 398 and sunitinib (SU) (A) or regorafenib (REGO) (B) observed for IM-resistant GIST 430 cells; Table S1: Synergy scores between BGJ 398 or regorafenib (REGO) and crioitinib or lapatinib in GIST T-1R cells; Table S2: Primers used for real time qPCR. References [78,79] are cited in the Supplementary Materials.
