# Supplementary material for: Fibroblast Growth Factor 2 (FGF2) Activates Vascular Endothelial Growth Factor (VEGF) Signaling in Gastrointestinal Stromal Tumors (GIST): An Autocrine Mechanism Contributing to Imatinib Mesylate (IM) Resistance
Source: Cancers (Basel). 2024 Sep 7;16(17):3103. doi: 10.3390/cancers16173103 (PMC11394061; doi:10.3390/cancers16173103)
Supplement: Supplementary file 1 [file cancers-16-03103-s001.zip › cancers-3146265-supplementary.pdf]

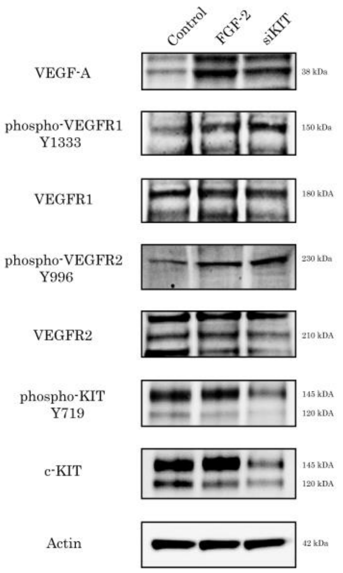

Supplementary Figure S1. Activation of VEGFR signaling in GIST T-1 cells upon knockout of the *KIT* gene. GIST T-1 cells were transfected with scrambled siRNA (control) or siRNA targeting *KIT* for 48 h. Alternatively, the cells was cultured in the presence of FGF-2 (100ng/ml) for 48 hours. Expression of VEGF-A, total and phosphorylated forms of VEGFR1, 2 and KIT was assessed by immunoblot analysis. Actin stain was used as a loading control for each sample.

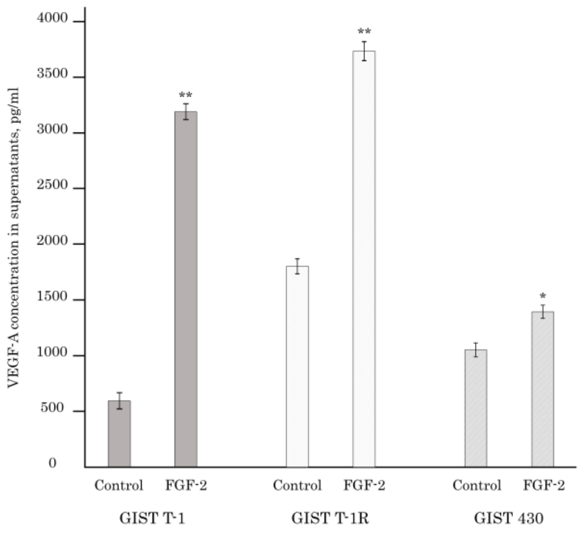

Supplementary Figure S2. Exogenous FGF2 increases VEGF production by IM-sensitive (GIST T-1) and resistant (GIST T-1R and GIST 430) cells. Concentration of VEGF-A (pg/ml, measured by ELISA) in supernatants of IM-sensitive (GIST T-1) and resistant (GIST T-1R and GIST 430) cells treated with DMSO (control) and FGF-2 (100 ng/mL) for 72 h. Data are presented as median ± SD. Significant differences with $p < 0.05$ (*), $p < 0.01$ (**) from $n \geq 3$ using unpaired Student's t-test.

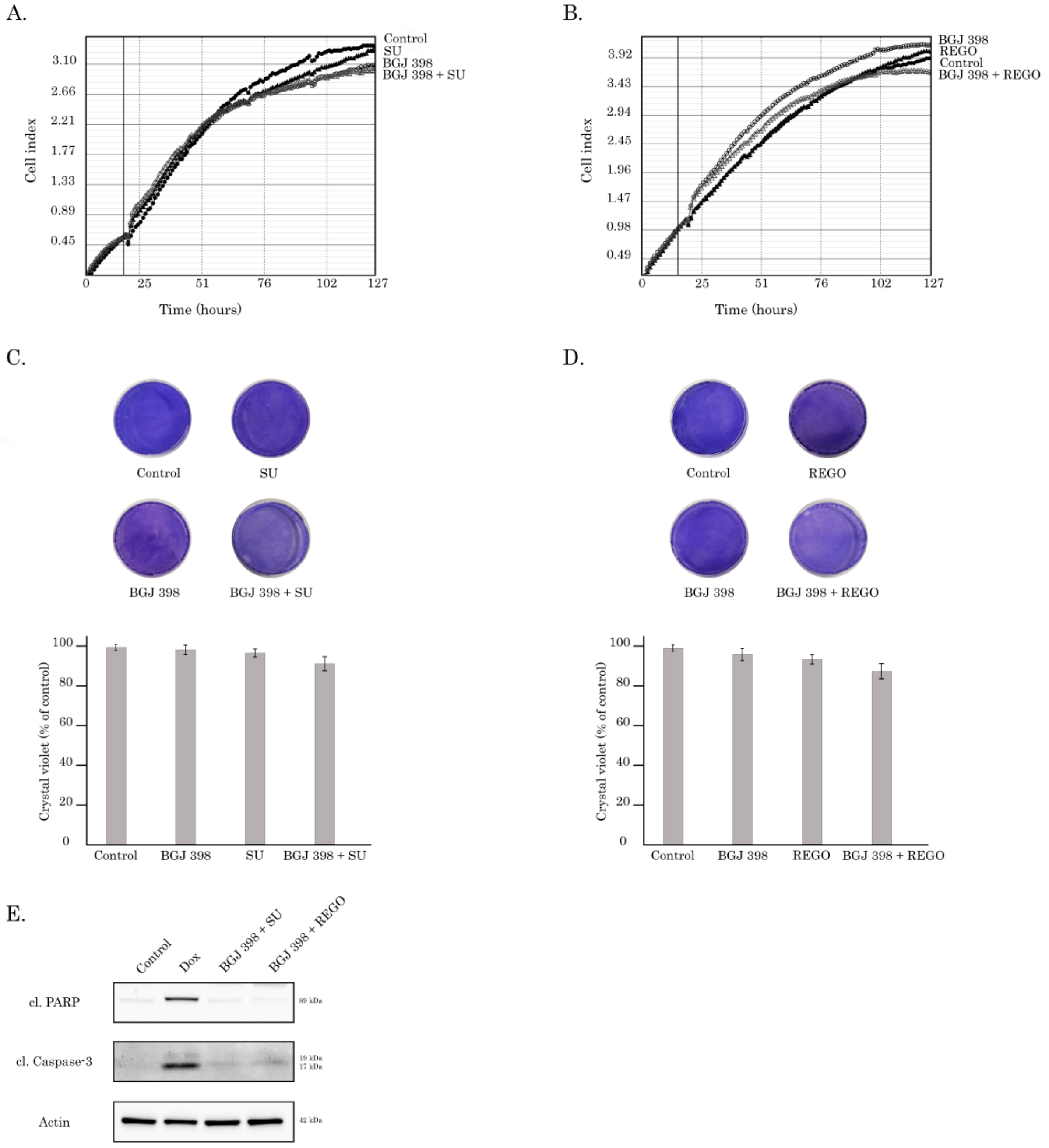

Supplementary Figure S3. Pro-apoptotic and anti-proliferative activities of BGJ 398 used in combination with sunitinib (SU) or regorefenib (REGO) in GIST 430 cells. All RTKis were used at 1 μM concentration. Cells were treated with RTKis for 72 h. **(A)** Changes in growth kinetics of GIST 430 cells treated with DMSO (control), SU or BGJ 398 alone and in combination; **(B)** Changes in growth kinetics of GIST 430 cells treated with DMSO (control), REGO or BGJ 398 alone and in combination; **(C)** *Upper panel* **-** Representative images of crystal violet staining of GIST 430 cells treated with SU or BGJ 398 alone or in combination. *Lower panel* — quantification of crystal violet staining of GIST 430 cells, as shown in the upper panel. **(D)** *Upper panel* **-** Representative images of crystal violet staining of GIST 430 cells treated with REGO or BGJ 398 alone or in combination. *Lower panel* **-** quantification of crystal violet staining of GIST cells, as shown in the upper panel. The culture dishes for (C) and (D) were stained with crystal violet and photographed.The cells treated with DMSO were used as a control. Quantification of

crystal violet staining of GIST cells is described in *Materials and Methods.* **(E)** WB data illustrating the expression of apoptotic markers - cleaved forms of PARP and caspase-3 in GIST 430 cells treated with combination of BGJ 398 with SU or REGO. Doxorubicin (Dox) (2 μM) - treated cells were used as a positive control. Actin staining was used to show the comparable amounts of protein loaded into each sample.

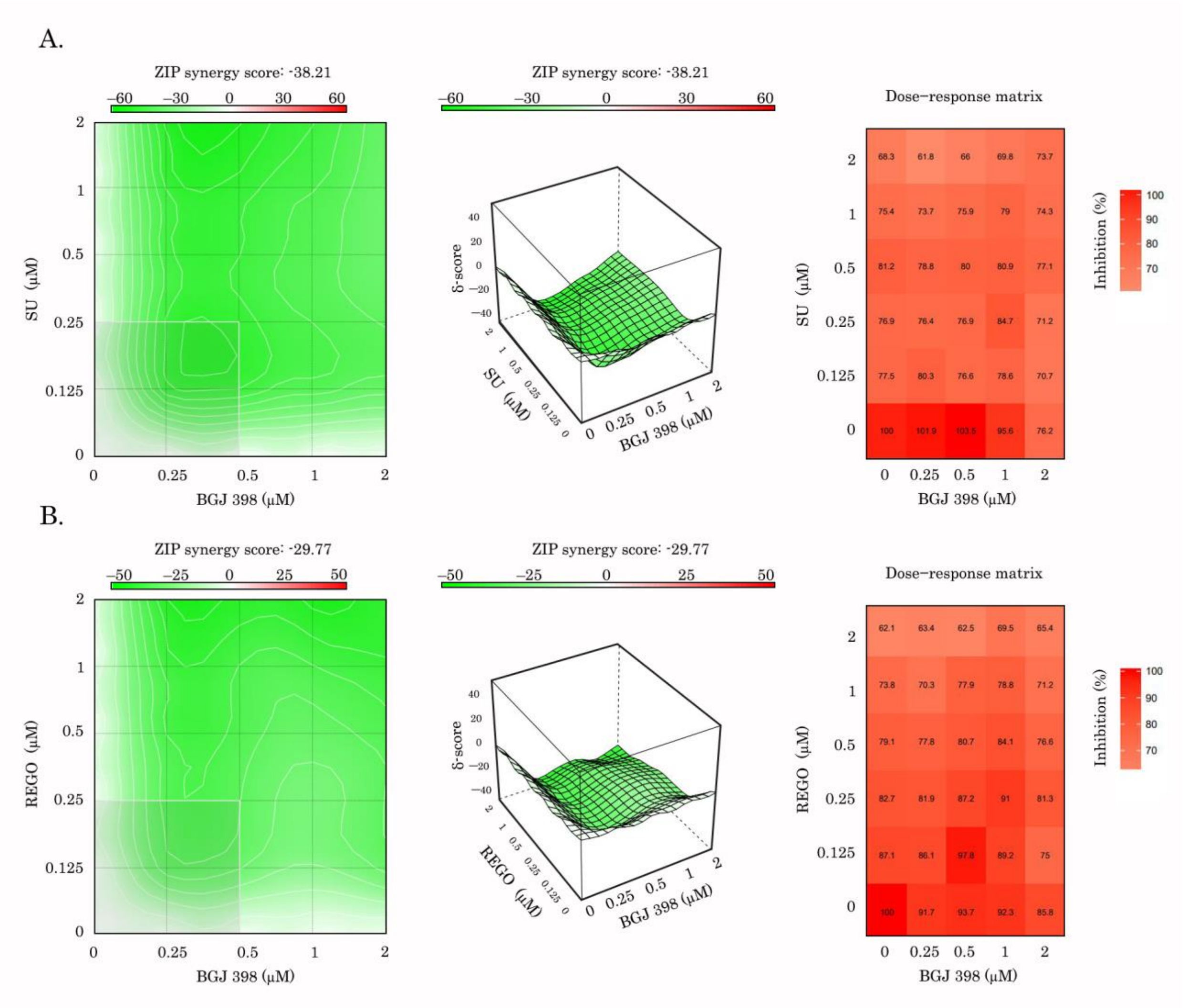

Supplementary Figure S4. Assessment of the synergy between BGJ 398 and sunitinib (SU) **(A)** or regorafenib (REGO) **(B)** observed for IM-resistant GIST 430 cells (ZIP model).

Supplementary Table S1. Synergy scores between BGJ 398 or regorafenib (REGO) and crisoitinib or lapatinib in GIST T-1R cells

RTKi	RTKi	ZIP	Bliss	Loewe	HSA
BGJ398	Crisotinib	0.04	-0.75	-0.30	2.57
	Lapatinib	13.89	13.67	-6.25	13.67
REGO	Crisotinib	-3.96	-8.09	-8.23	-0.60
	Lapatinib	-13.16	-13.19	-6.53	0.30

Supplementary Table S2. Primers used for real time qPCR

Gene	Forward sequence	Reverse sequence	Source
VEGFA	CGAGTACATCTTCAAGCCATCC	TGGTGAGGTTTGATCCGC	[78]
VEGFR1	GGCTCTGTGGAAAGTTCAGC	GCTCACACTGCTCATCCAAA	[79]
VEGFR2	TGCTTCACAGAAGACCATGC	GTGACCAACATGGAGTCGTG	
VEGFR3	GAGACAAGGACAGCGAGGAC	TCACGAACACGTAGGAGCTG	
