# Supplementary material for: Fibroblast Growth Factor 2 (FGF2) Activates Vascular Endothelial Growth Factor (VEGF) Signaling in Gastrointestinal Stromal Tumors (GIST): An Autocrine Mechanism Contributing to Imatinib Mesylate (IM) Resistance
Source: Cancers (Basel). 2024 Sep 7;16(17):3103. doi: 10.3390/cancers16173103 (PMC11394061; doi:10.3390/cancers16173103)
Supplement: Supplementary file 1 [file cancers-16-03103-s001.zip › File S1. original western blot figures.pdf]

Uncropped file (Original Images for blots)

Figure 1 A.

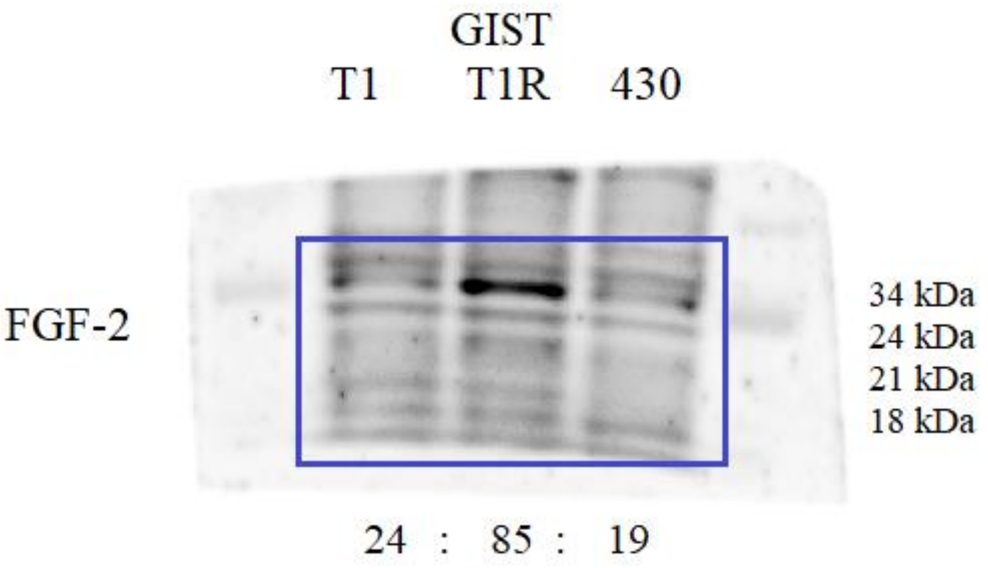

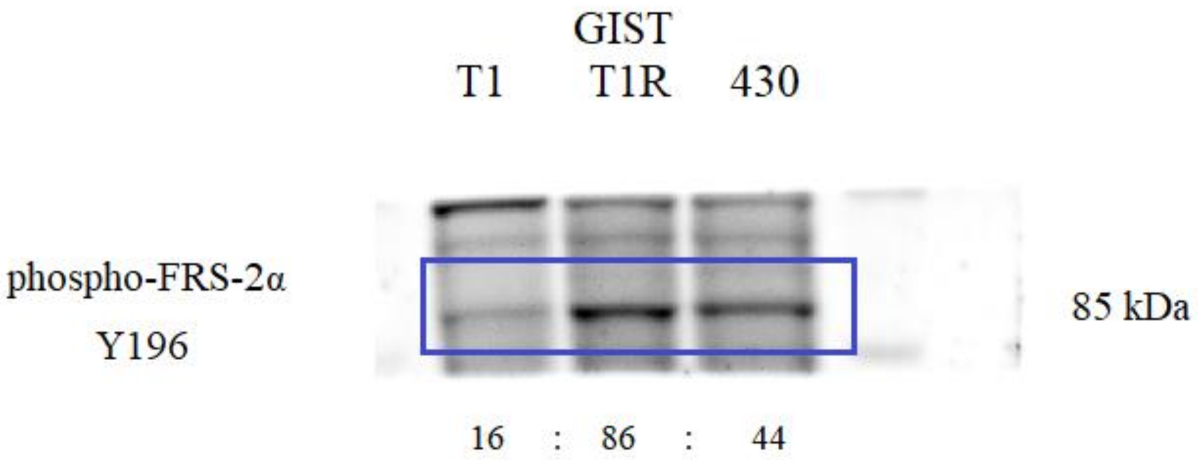

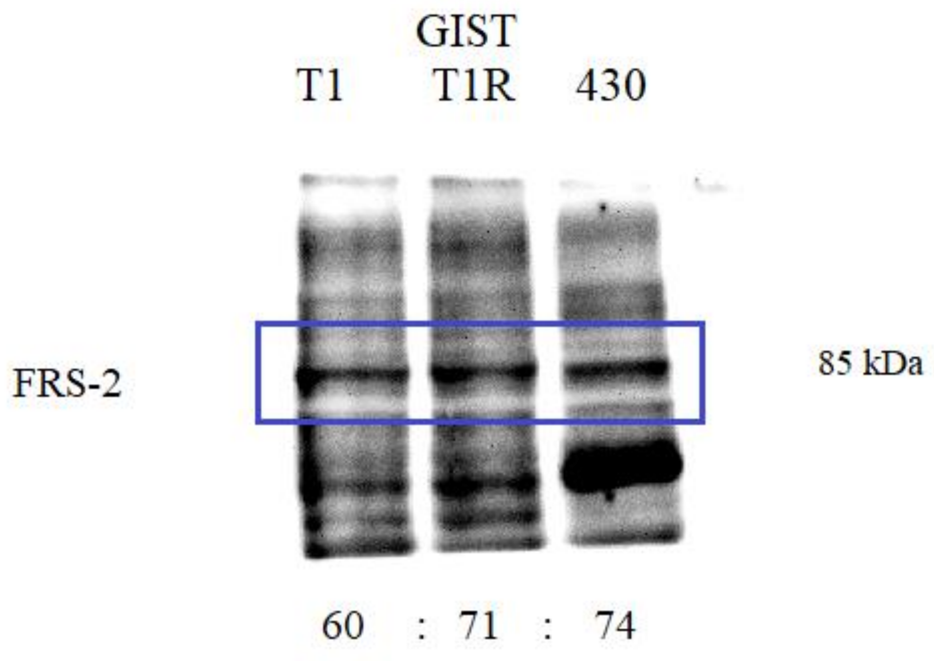
GIST
T1
T1R
430
FRS-2
85 kDa
60 : 71 : 74

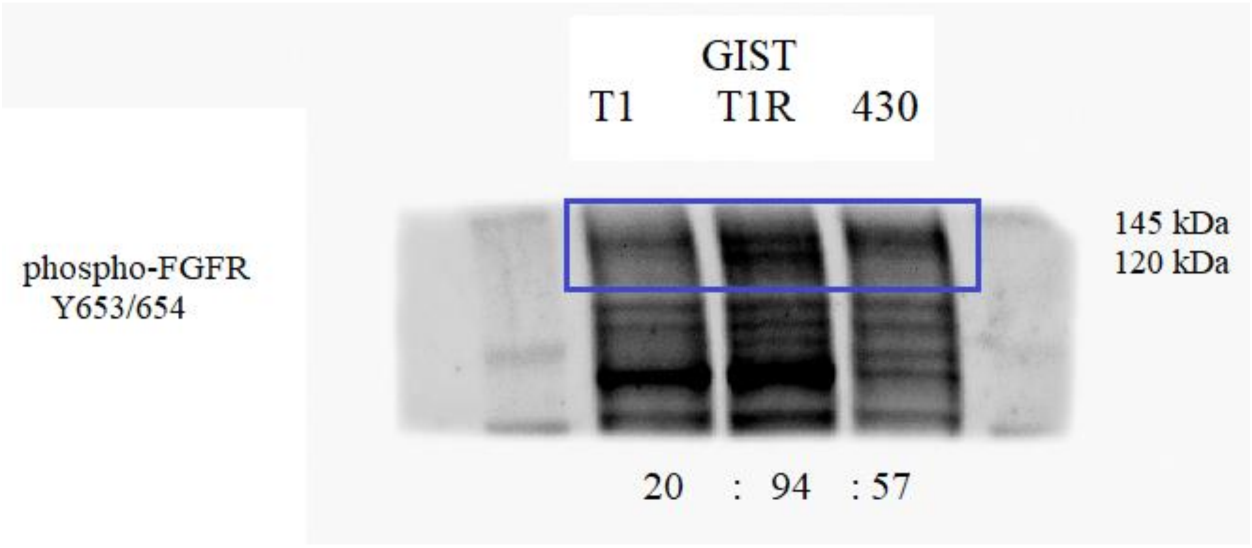
GIST
T1
T1R
430
phospho-FGFR
Y653/654
145 kDa
120 kDa
20 : 94 : 57

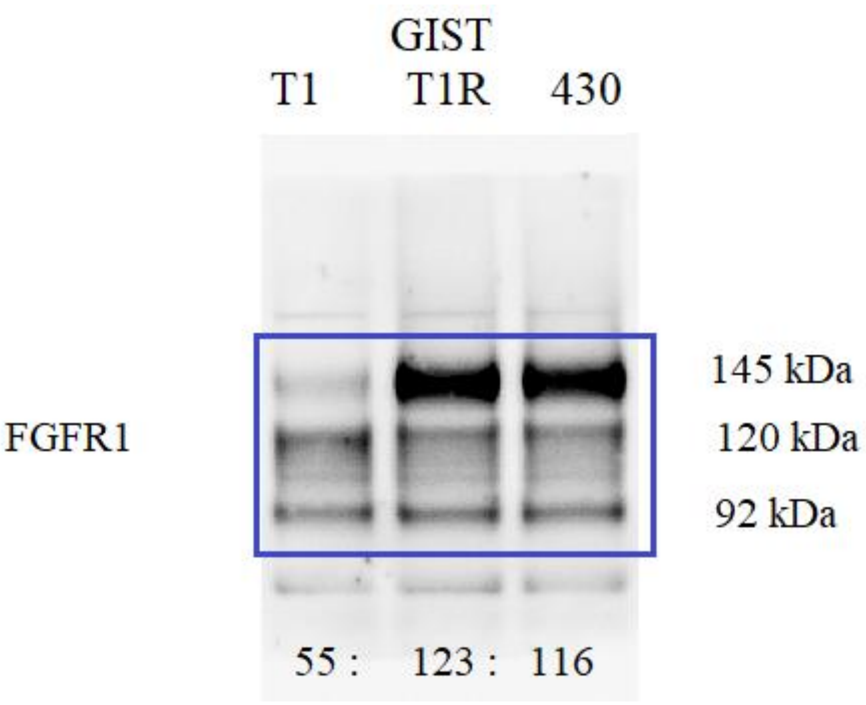
GIST
T1
T1R
430
FGFR1
145 kDa
120 kDa
92 kDa
55 : 123 : 116

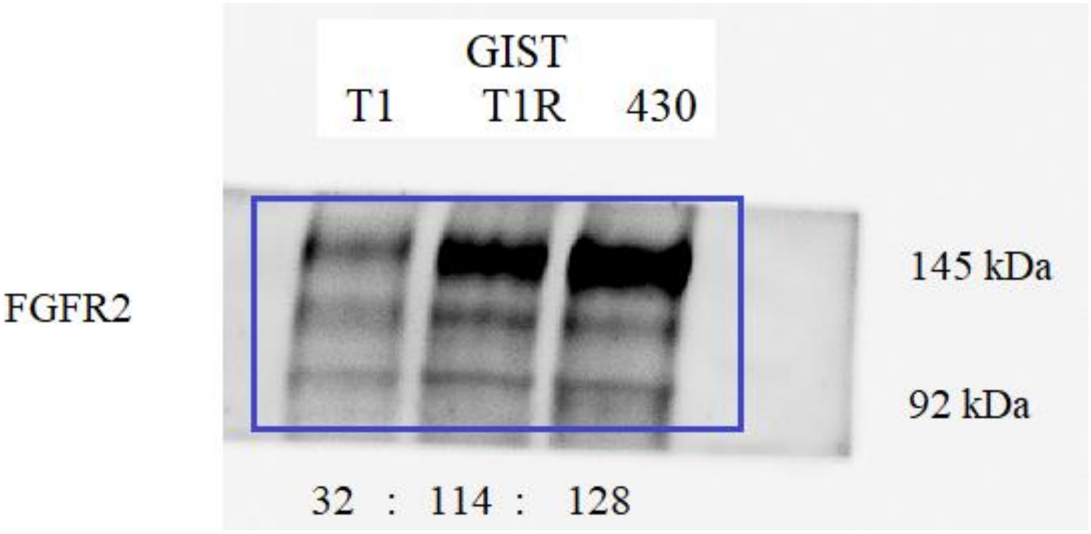
GIST
T1
T1R
430
FGFR2
145 kDa
92 kDa
32 : 114 : 128

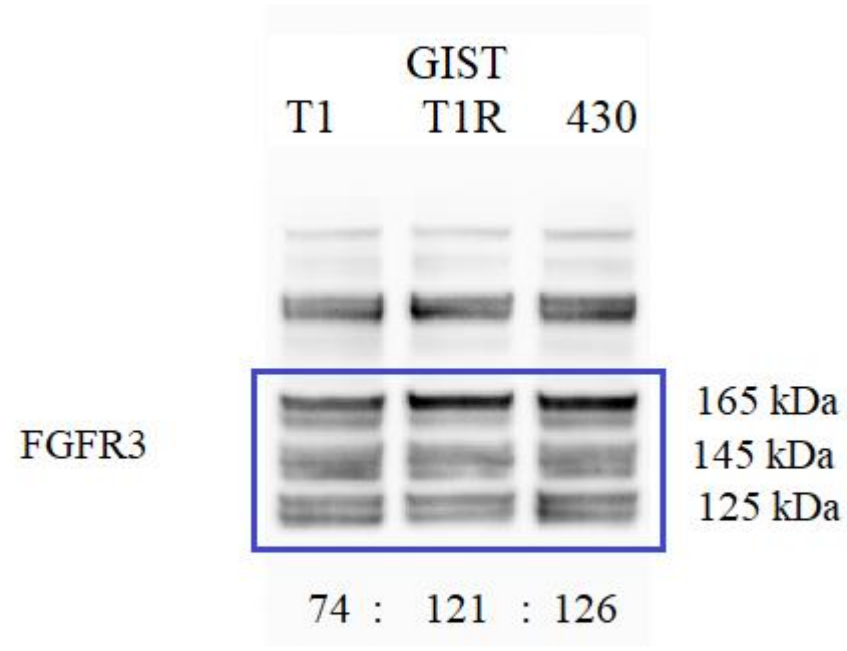
GIST
T1 T1R 430
FGFR3
165 kDa
145 kDa
125 kDa
74 : 121 : 126

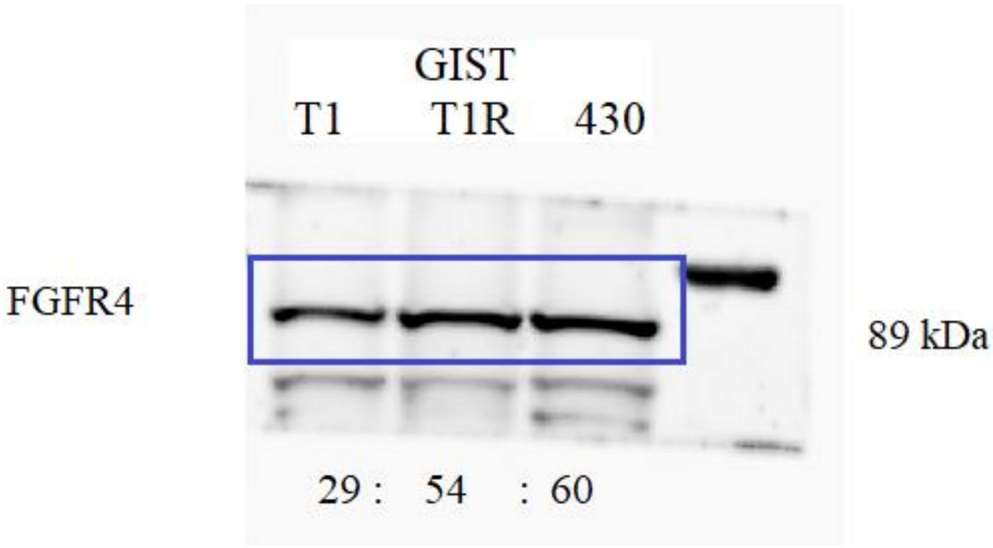
GIST
T1 T1R 430
FGFR4
89 kDa
29 : 54 : 60

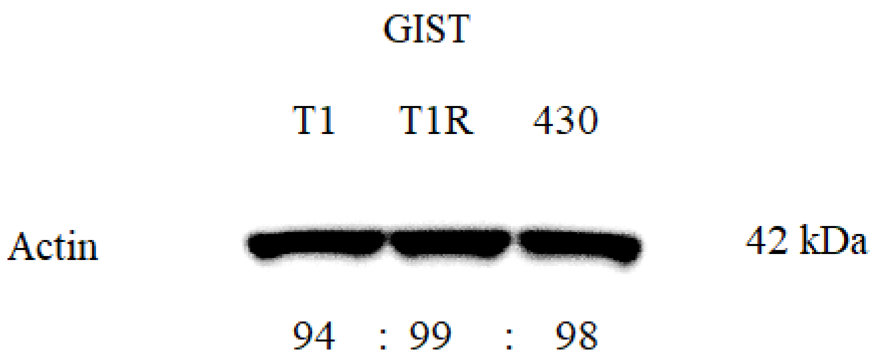
GIST
T1 T1R 430
Actin
42 kDa
94 : 99 : 98

Figure 1 B.

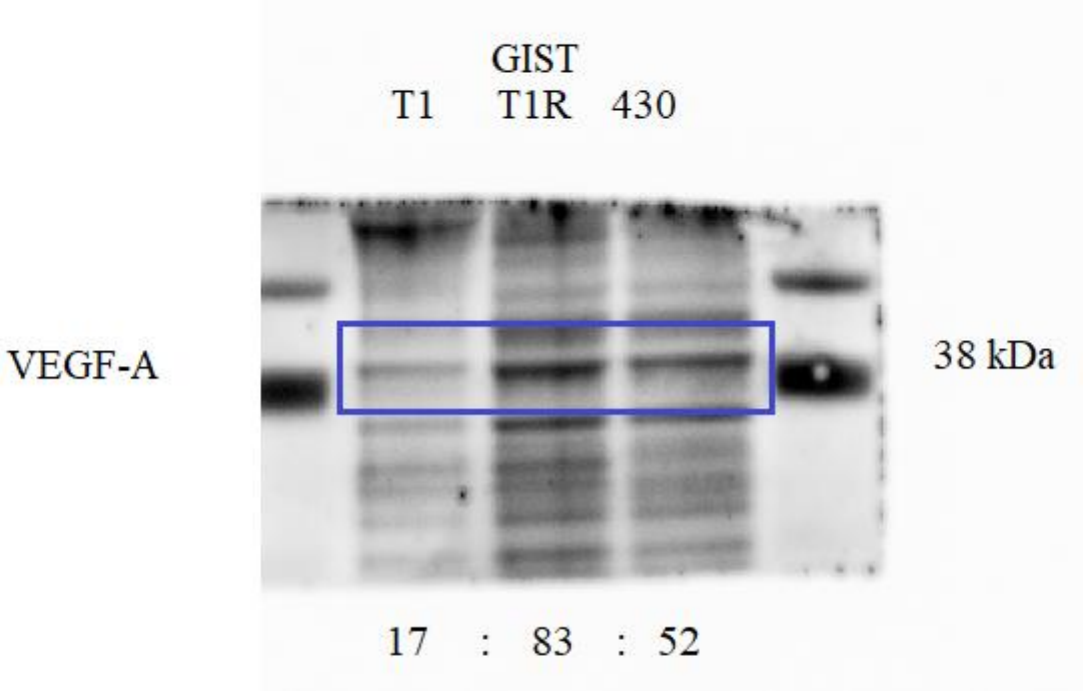

GIST
T1 T1R 430
VEGF-A
38 kDa
17 : 83 : 52

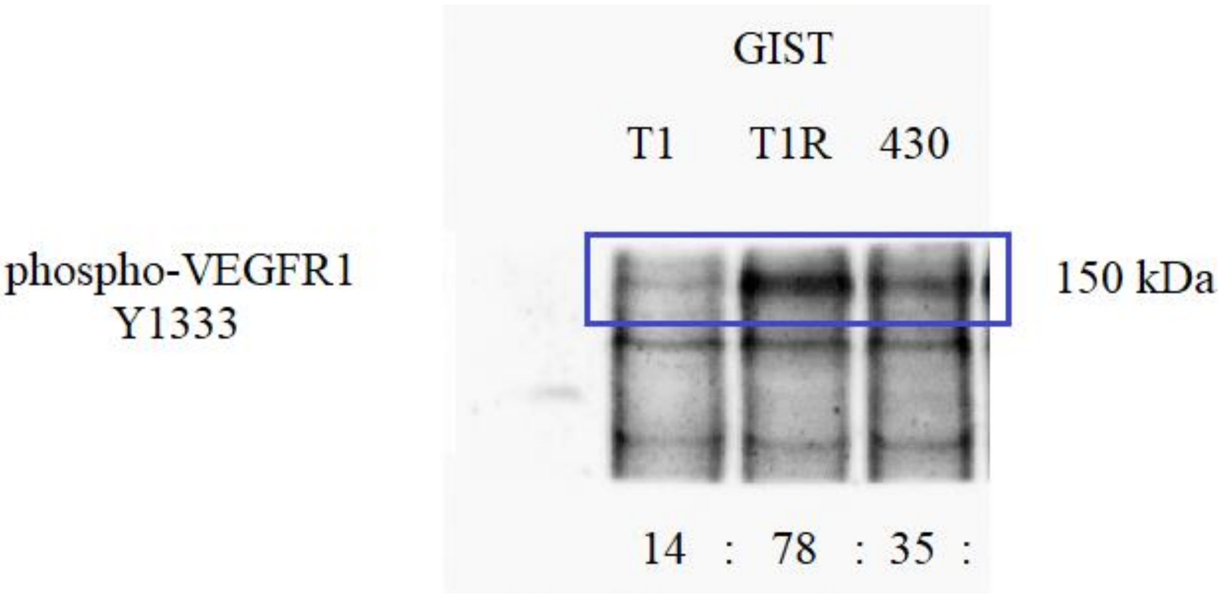

GIST
T1 T1R 430
phospho-VEGFR1
Y1333
150 kDa
14 : 78 : 35 :

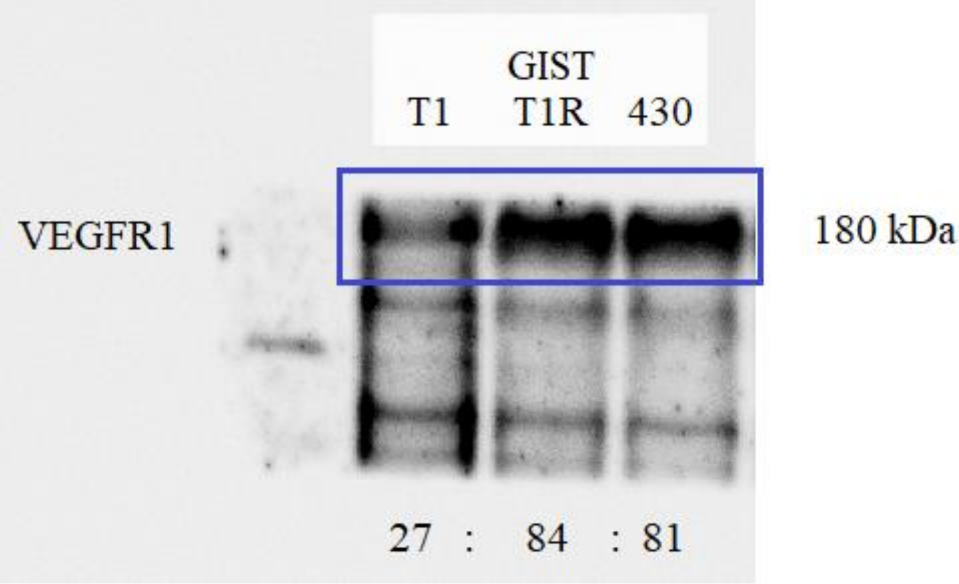
GIST
T1 T1R 430
VEGFR1
180 kDa
27 : 84 : 81

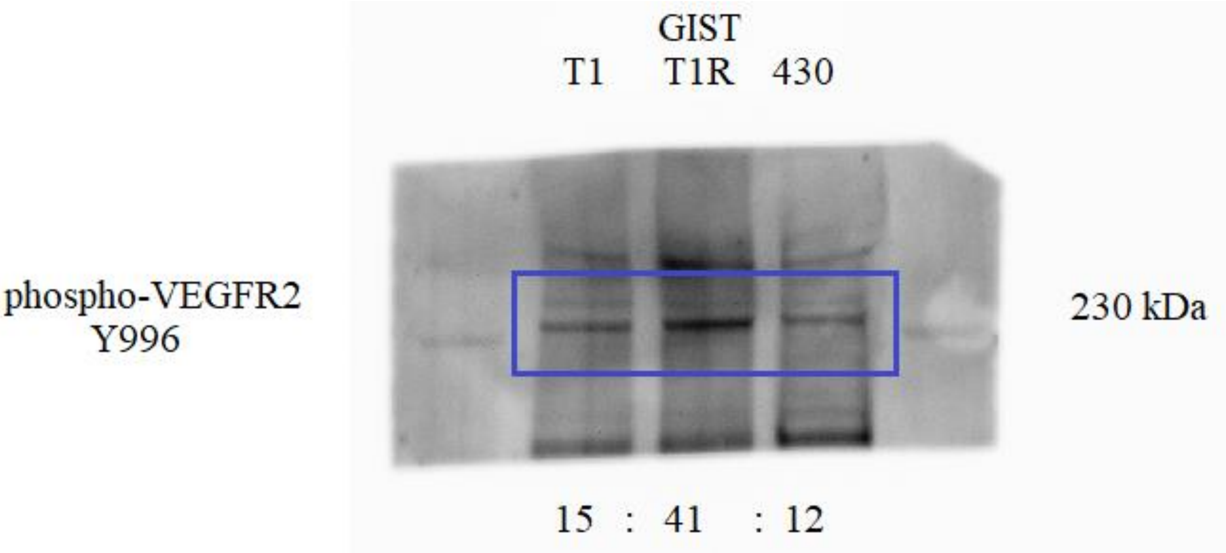
GIST
T1 T1R 430
phospho-VEGFR2
Y996
230 kDa
15 : 41 : 12

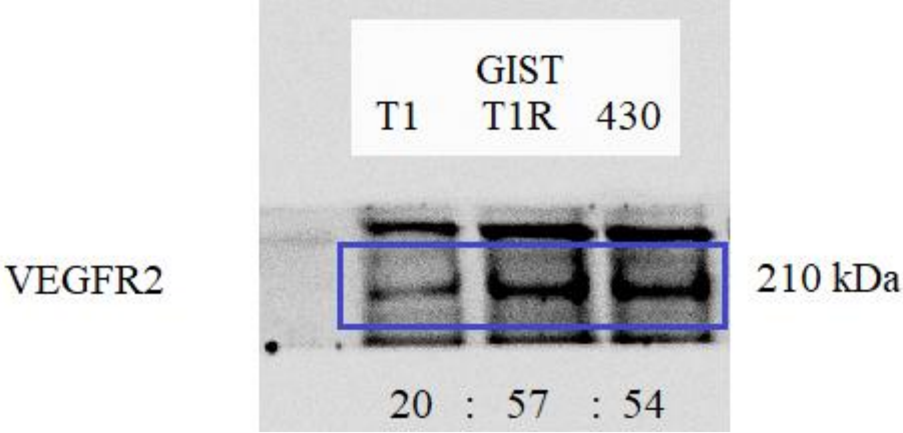
GIST
T1 T1R 430
VEGFR2
210 kDa
20 : 57 : 54

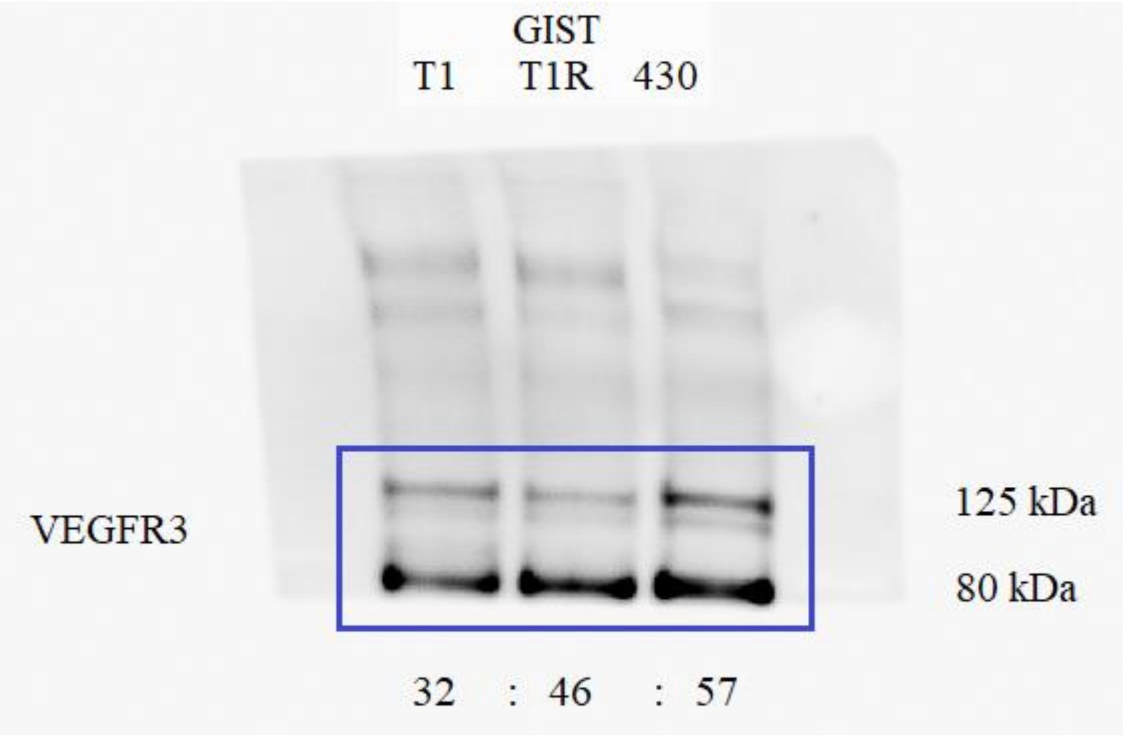
GIST
T1
T1R
430
VEGFR3
125 kDa
80 kDa
32 : 46 : 57

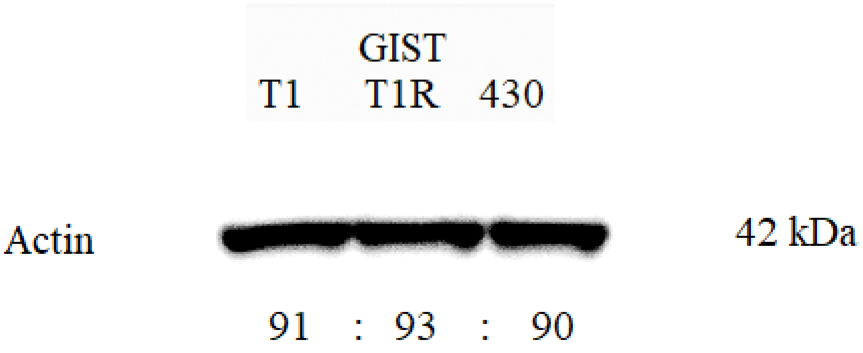
GIST
T1
T1R
430
Actin
42 kDa
91 : 93 : 90

Figure 1 D.

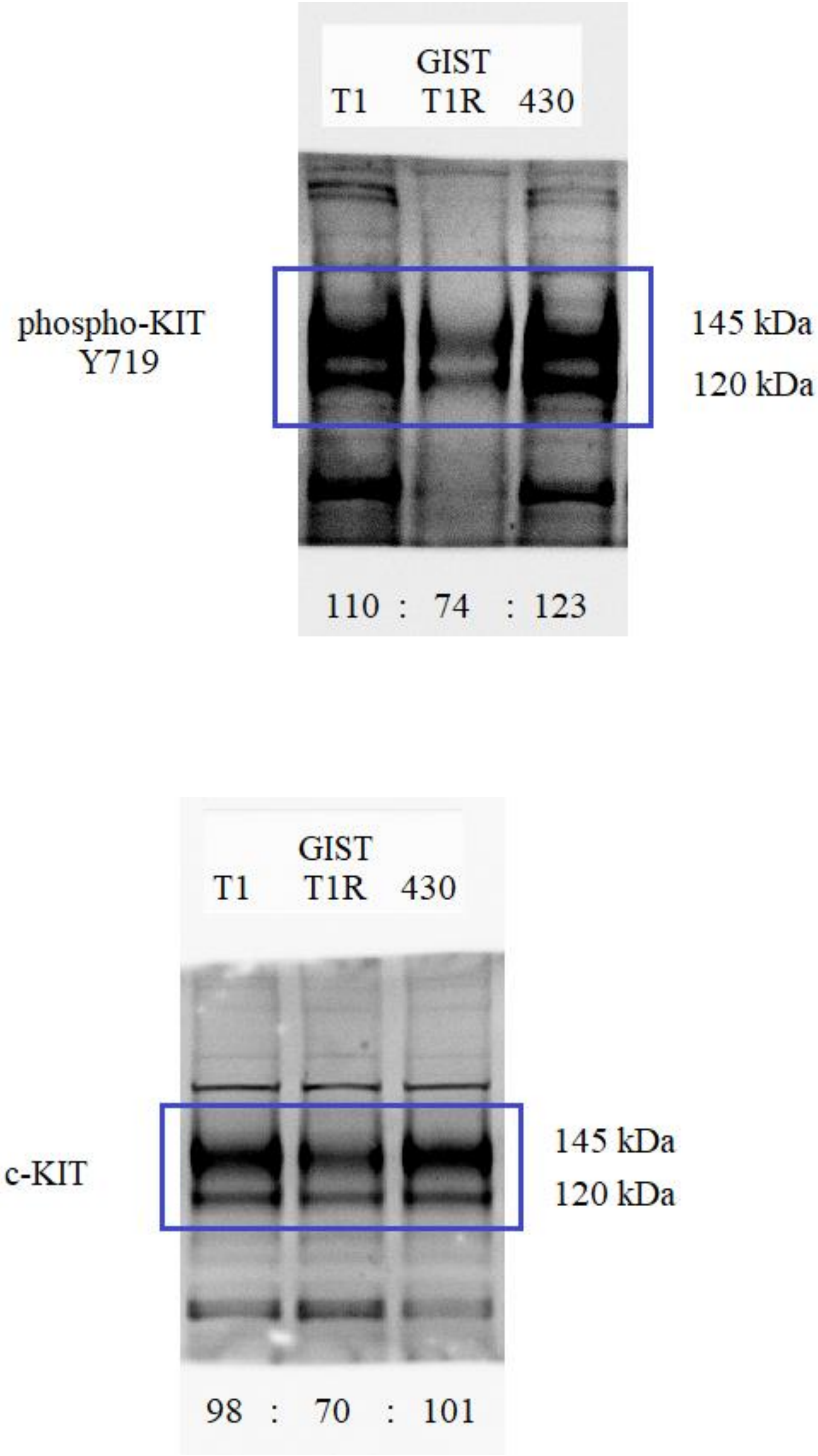
GIST
T1
T1R
430
phospho-KIT
Y719
145 kDa
120 kDa
110 : 74 : 123
GIST
T1
T1R
430
c-KIT
145 kDa
120 kDa
98 : 70 : 101

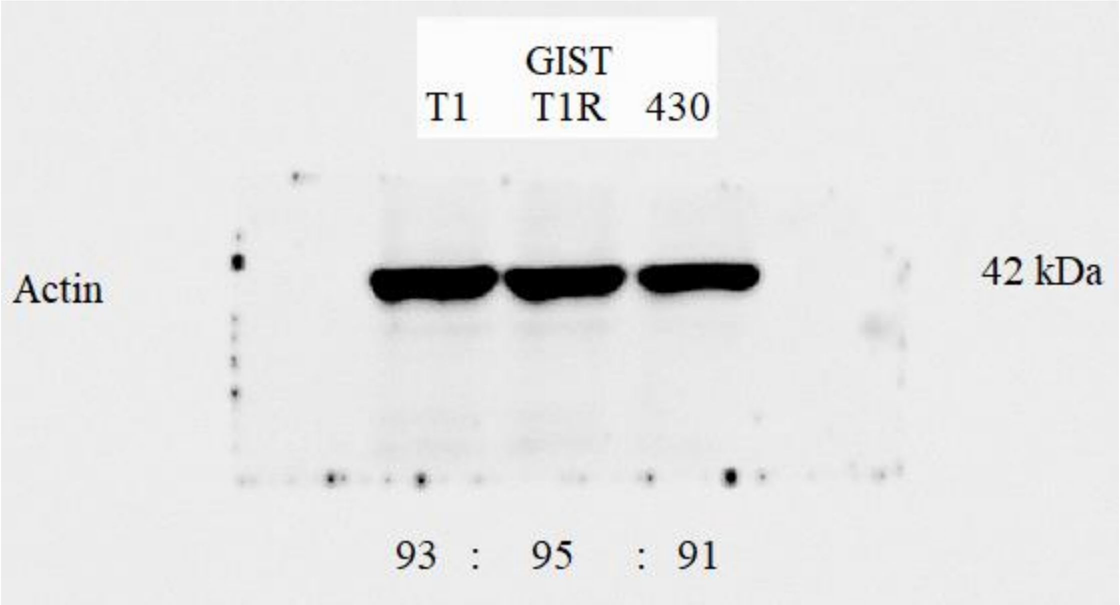

Figure 2 A. – Left

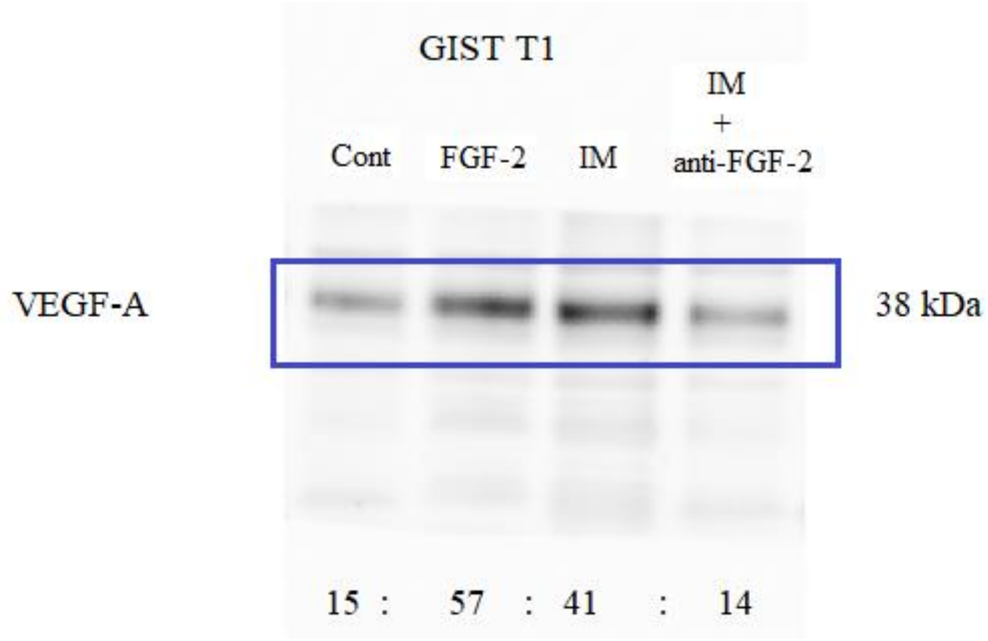

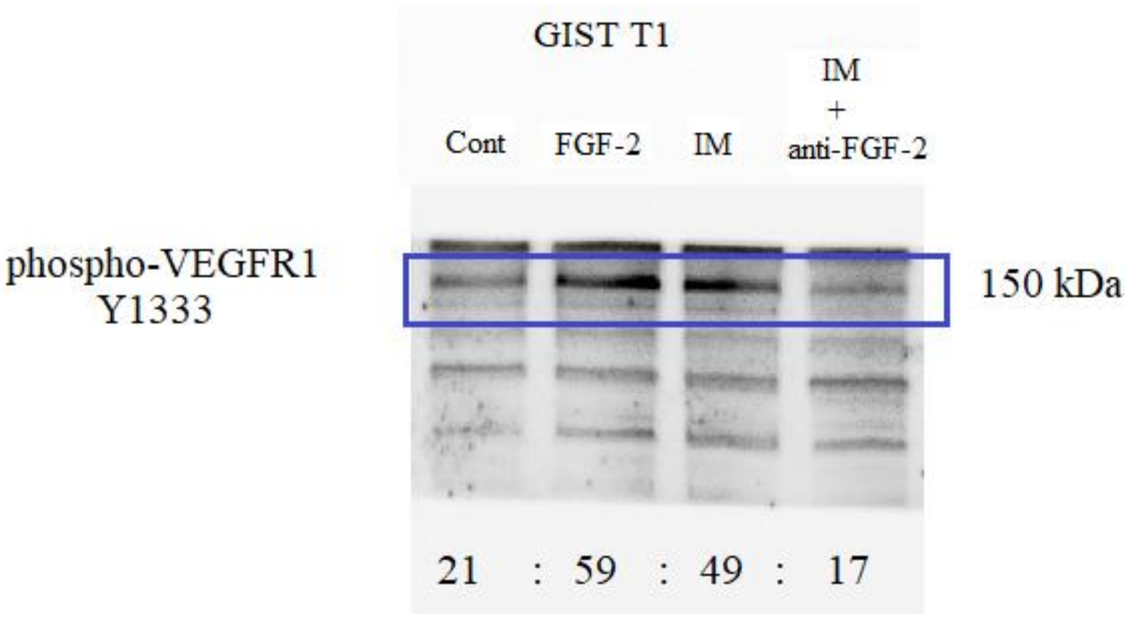
GIST T1
Cont
FGF-2
IM
IM + anti-FGF-2
phospho-VEGFR1 Y1333
150 kDa
21 : 59 : 49 : 17

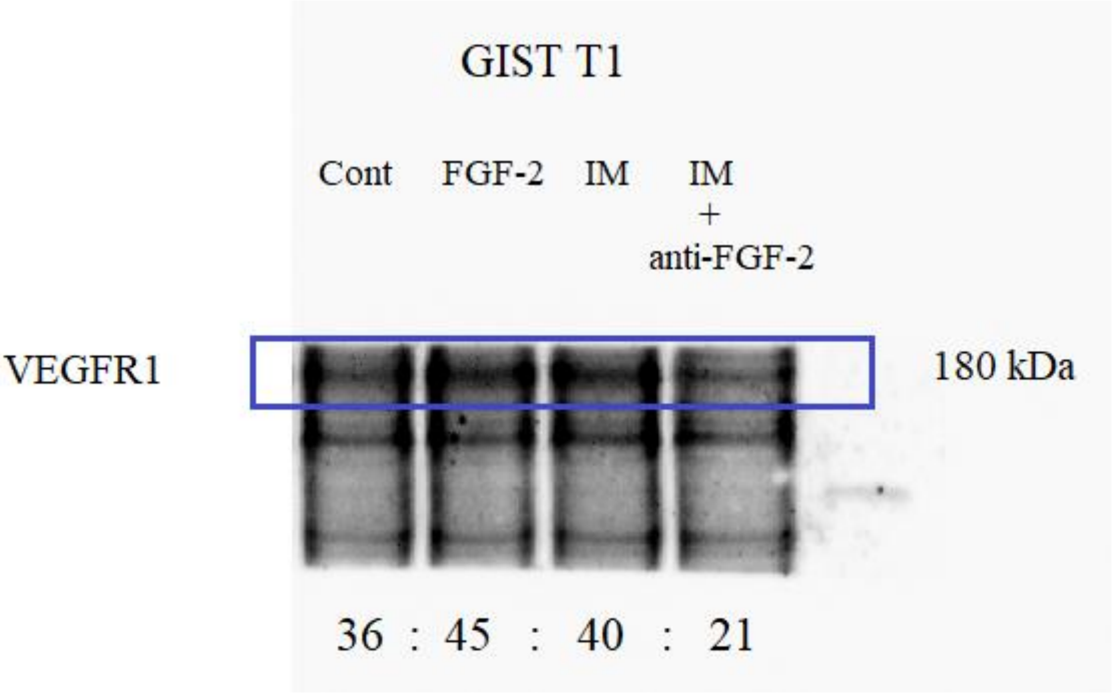
GIST T1
Cont
FGF-2
IM
IM + anti-FGF-2
VEGFR1
180 kDa
36 : 45 : 40 : 21

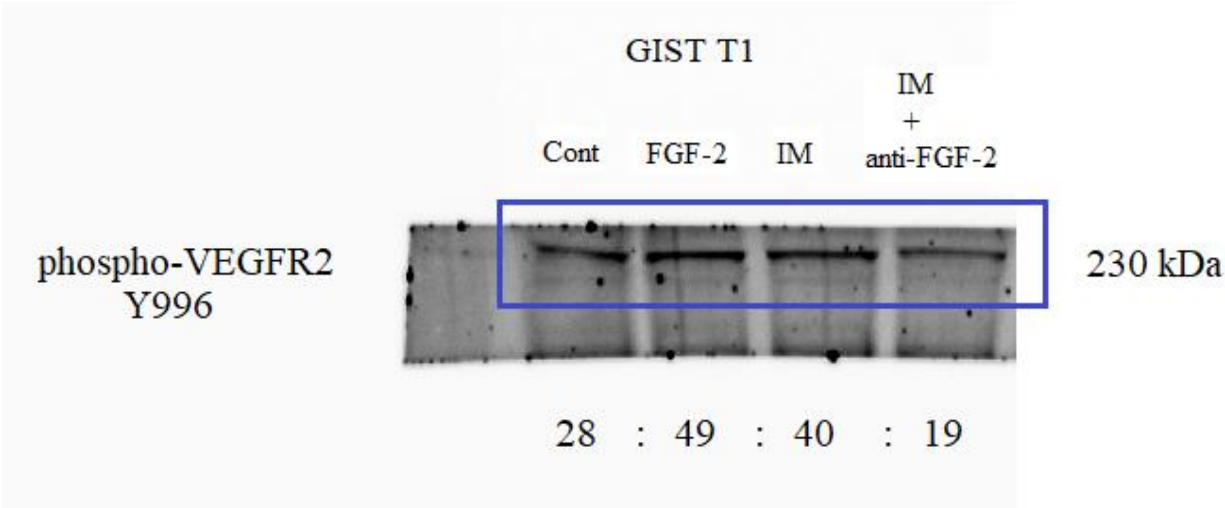
GIST T1
Cont
FGF-2
IM
IM + anti-FGF-2
phospho-VEGFR2 Y996
230 kDa
28 : 49 : 40 : 19

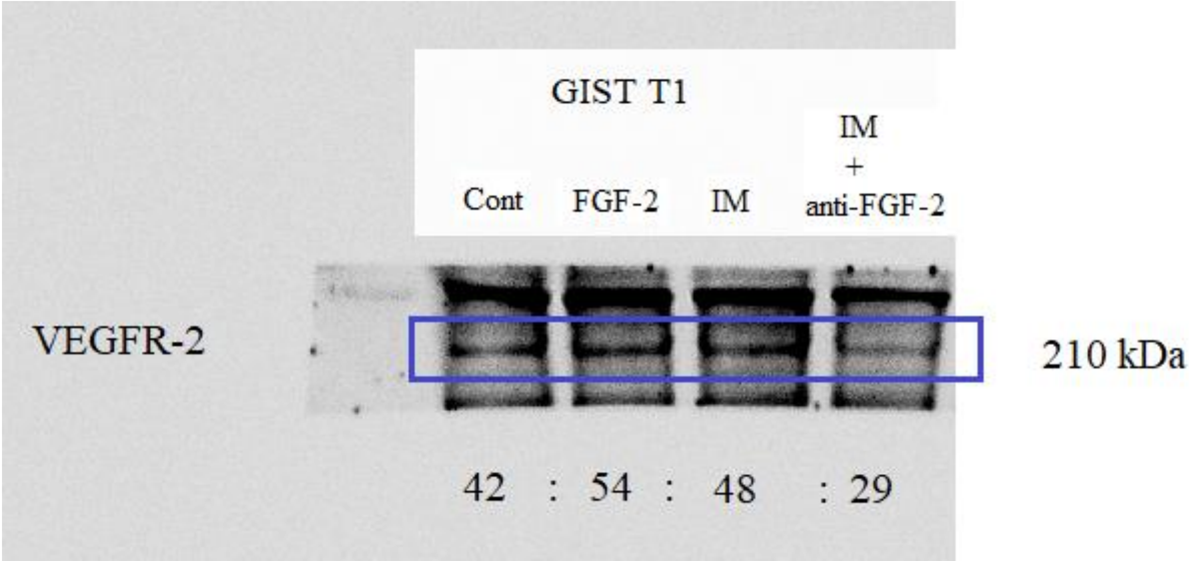
GIST T1
Cont
FGF-2
IM
IM + anti-FGF-2
VEGFR-2
210 kDa
42 : 54 : 48 : 29

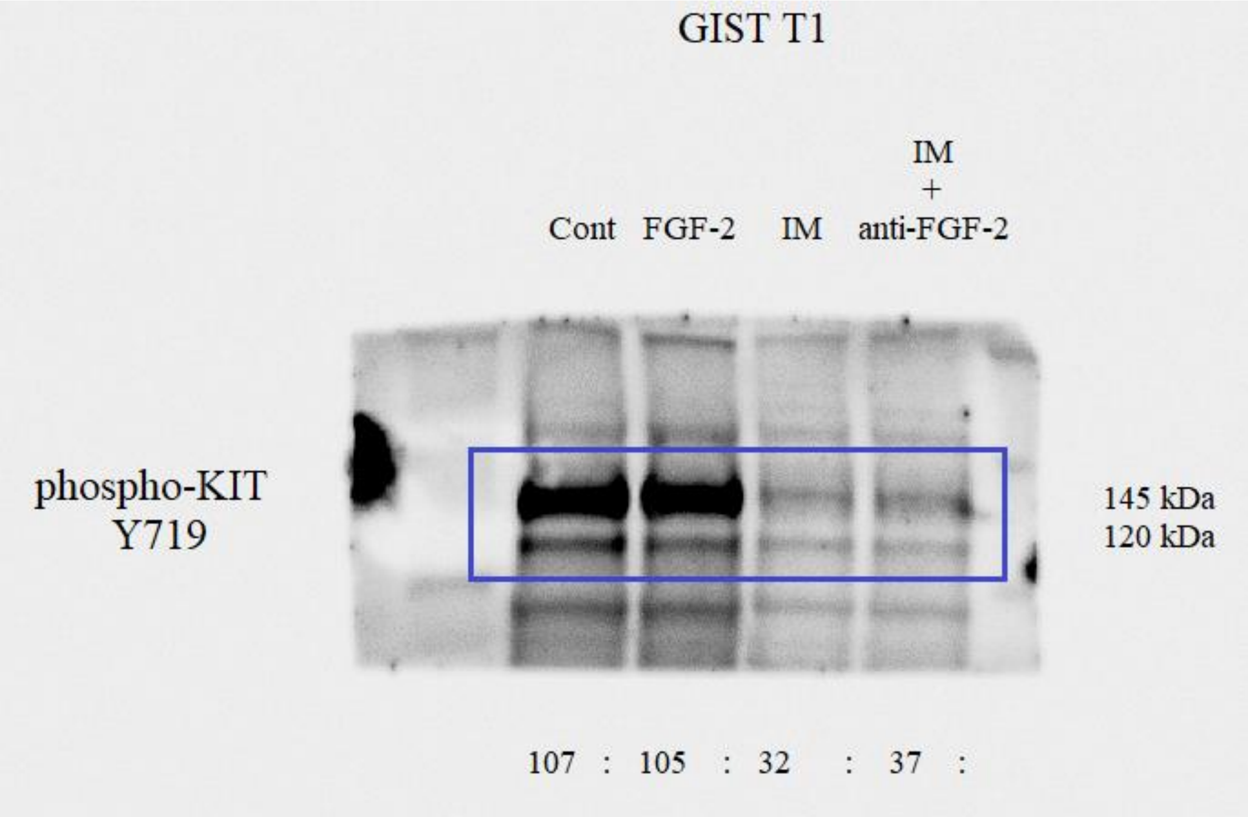
GIST T1
IM
+
Cont FGF-2 IM anti-FGF-2
phospho-KIT
Y719
145 kDa
120 kDa
107 : 105 : 32 : 37 :

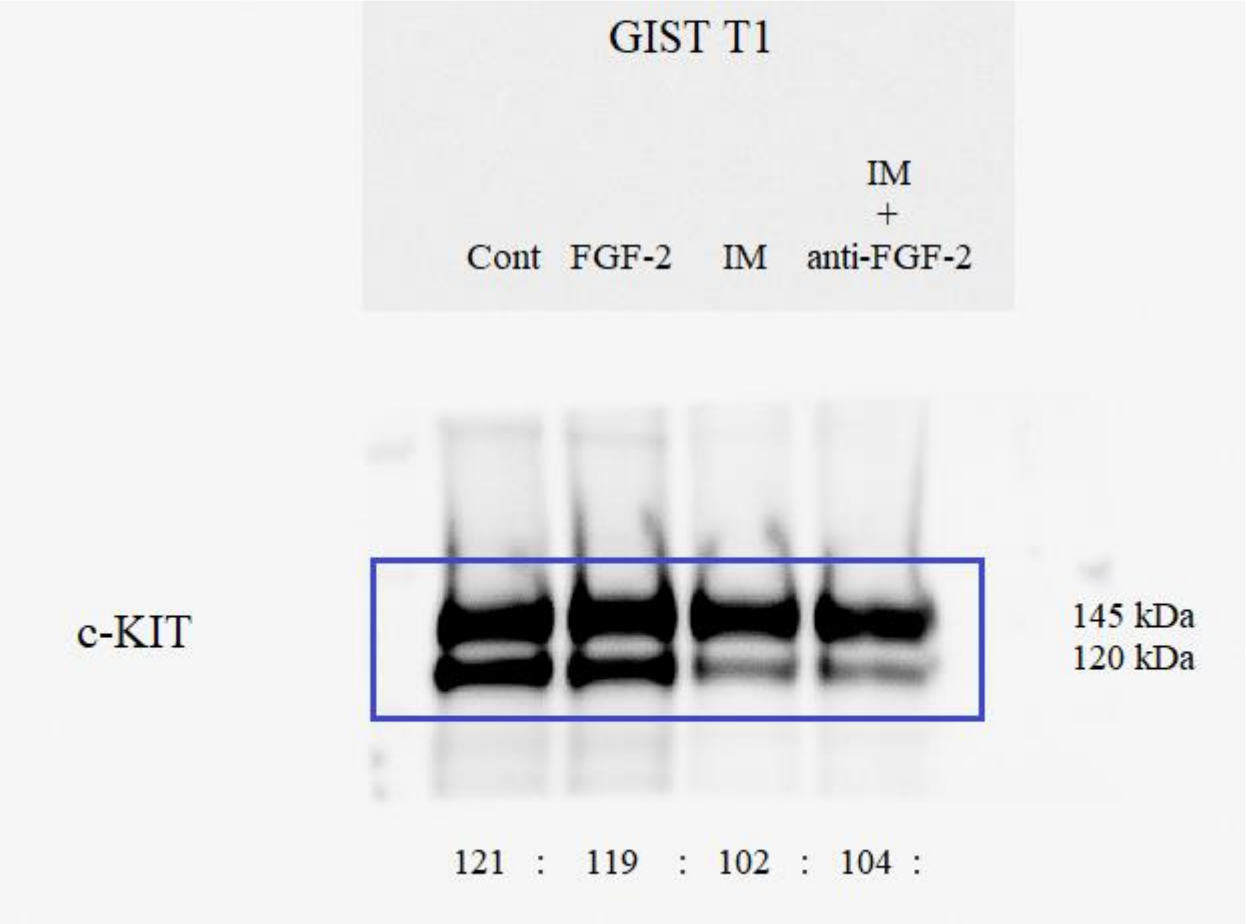
GIST T1
IM
+
Cont FGF-2 IM anti-FGF-2
c-KIT
145 kDa
120 kDa
121 : 119 : 102 : 104 :

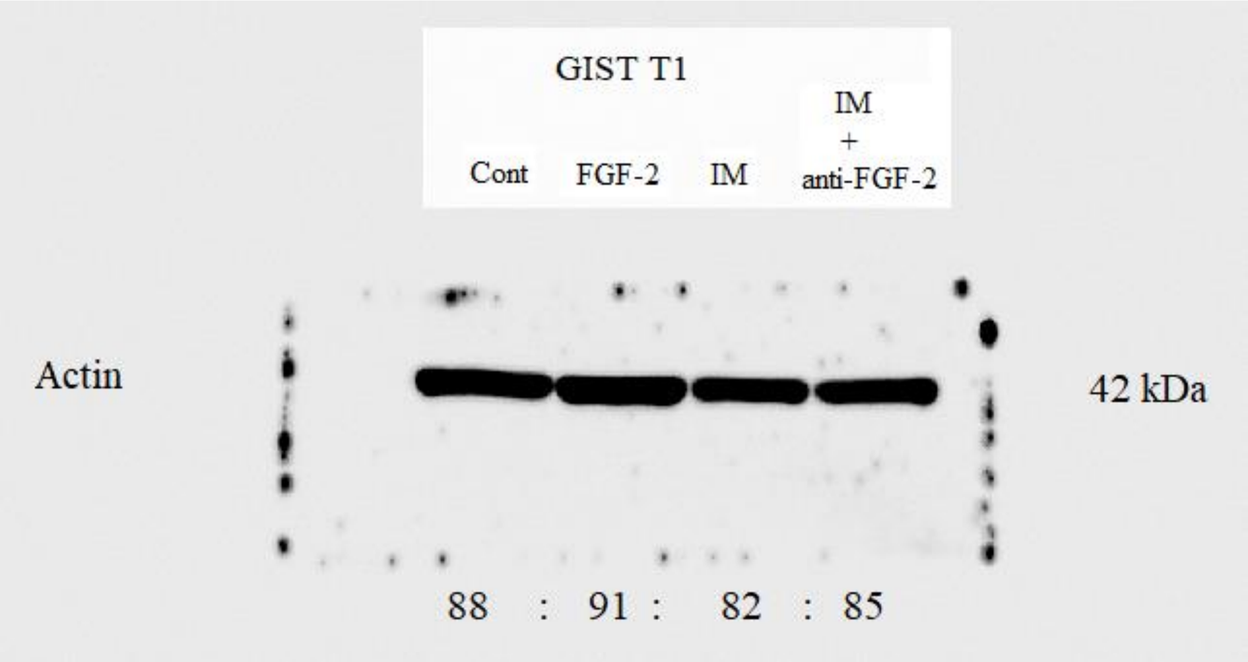

Figure 2 A. – Right

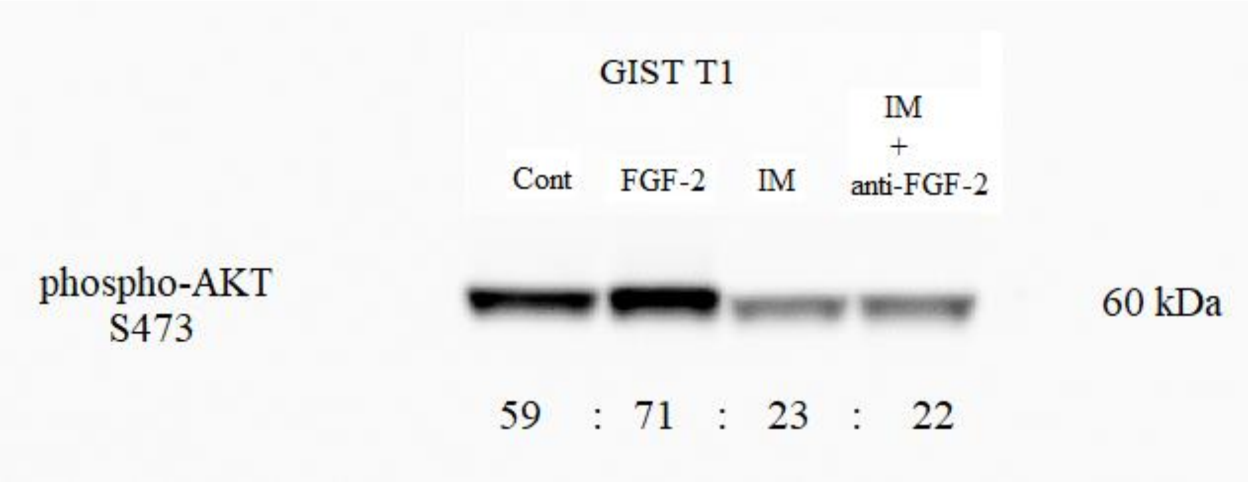

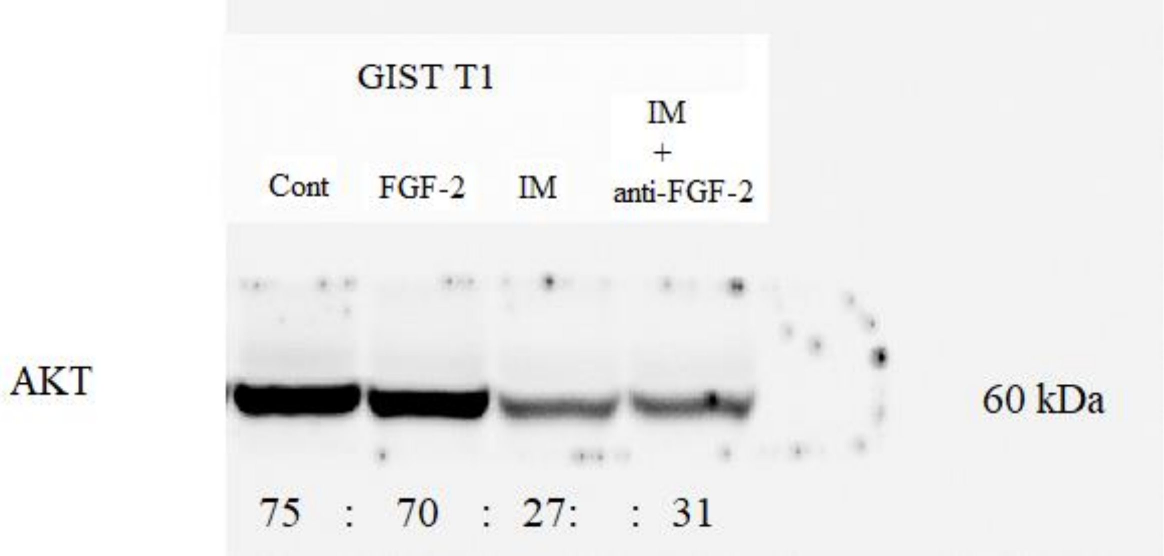
GIST T1
Cont
FGF-2
IM
IM + anti-FGF-2
AKT
60 kDa
75 : 70 : 27: : 31

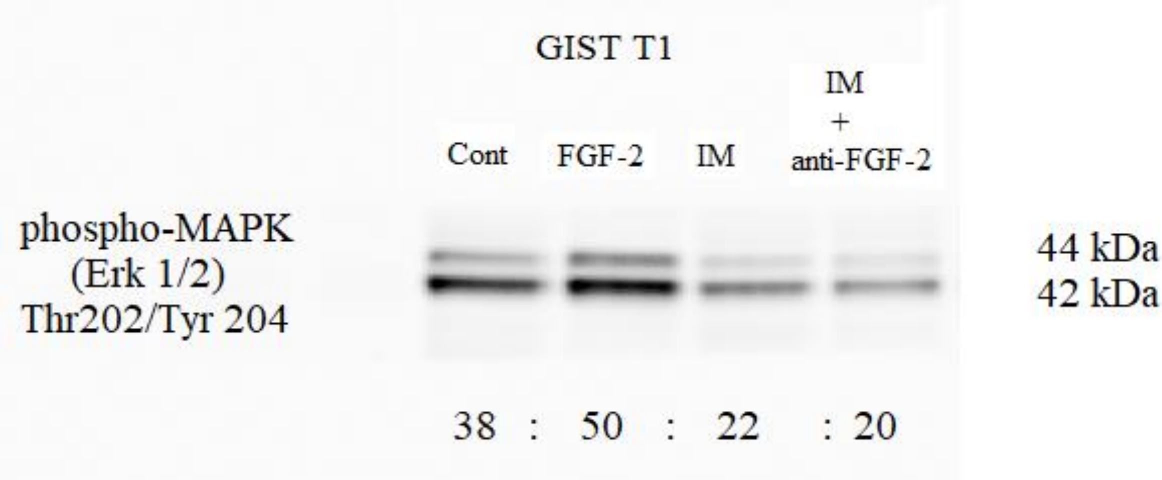
GIST T1
Cont
FGF-2
IM
IM + anti-FGF-2
phospho-MAPK (Erk 1/2) Thr202/Tyr 204
44 kDa
42 kDa
38 : 50 : 22 : 20

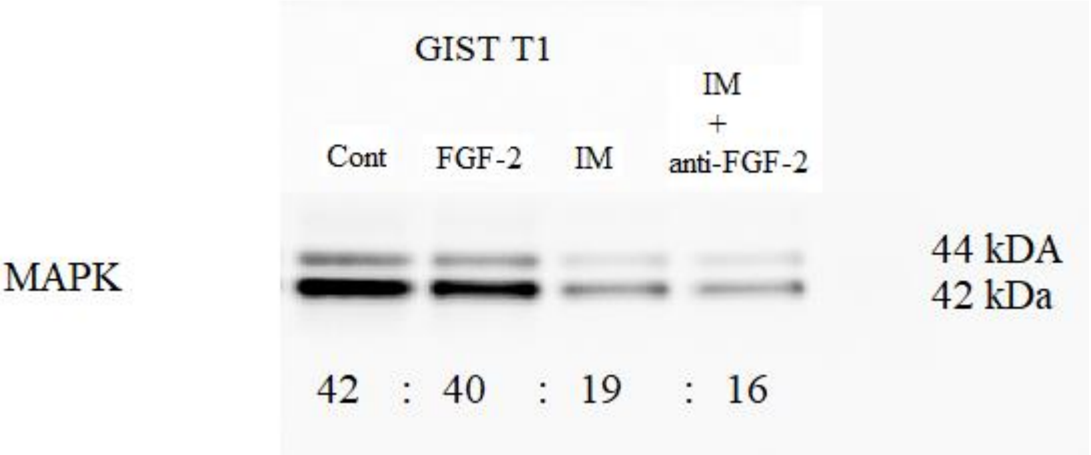
GIST T1
Cont
FGF-2
IM
IM + anti-FGF-2
MAPK
44 kDA
42 kDa
42 : 40 : 19 : 16

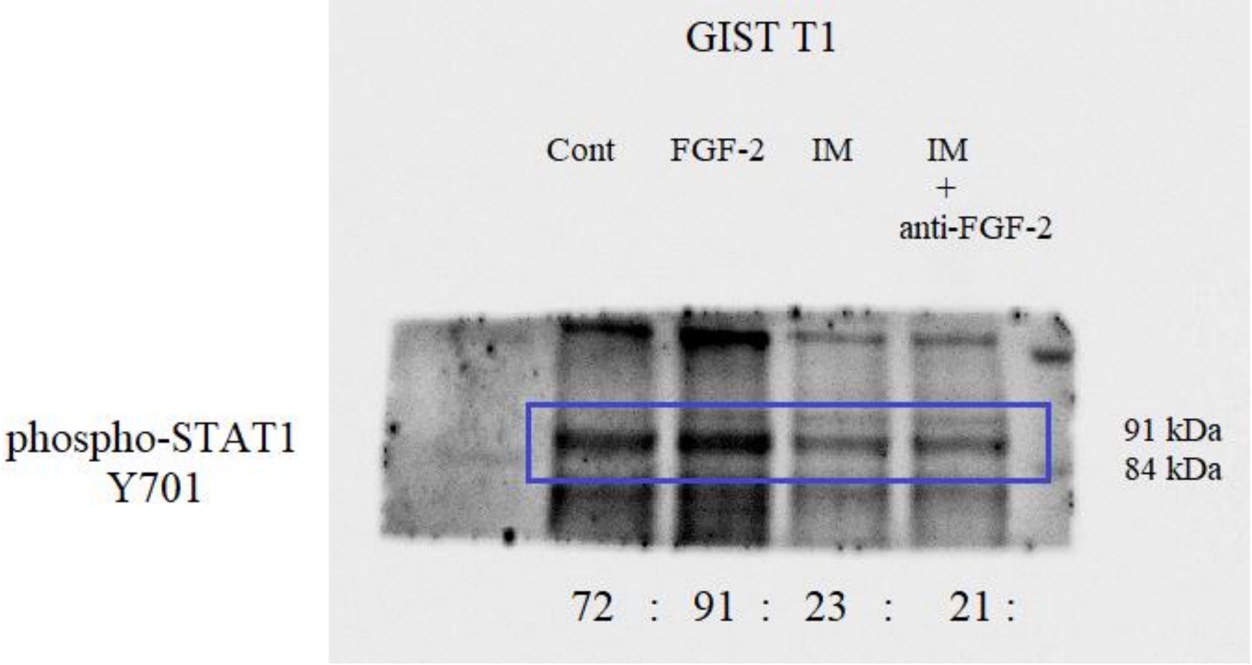
GIST T1
Cont
FGF-2
IM
IM + anti-FGF-2
phospho-STAT1 Y701
91 kDa
84 kDa
72 : 91 : 23 : 21 :

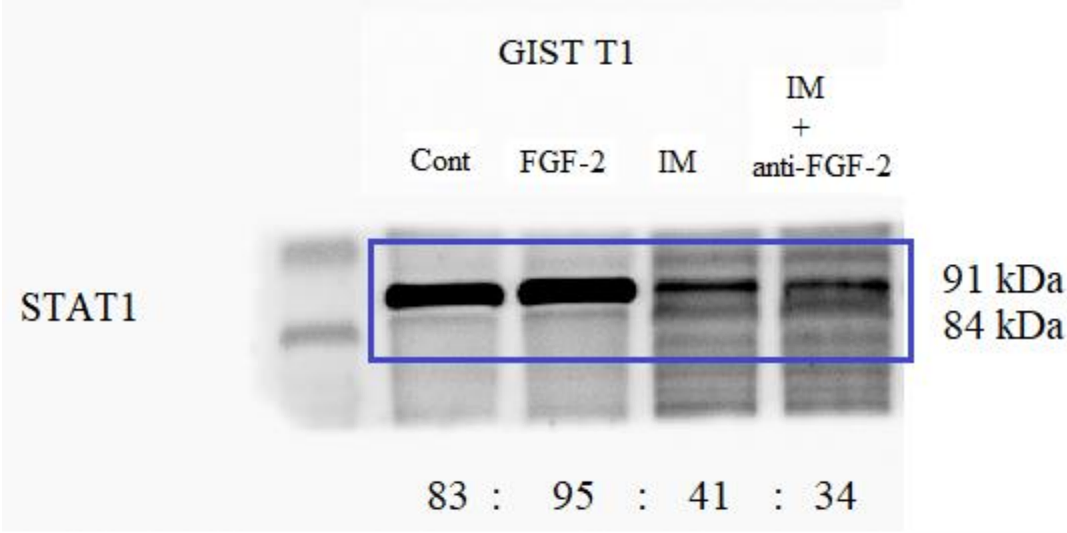
GIST T1
Cont
FGF-2
IM
IM + anti-FGF-2
STAT1
91 kDa
84 kDa
83 : 95 : 41 : 34

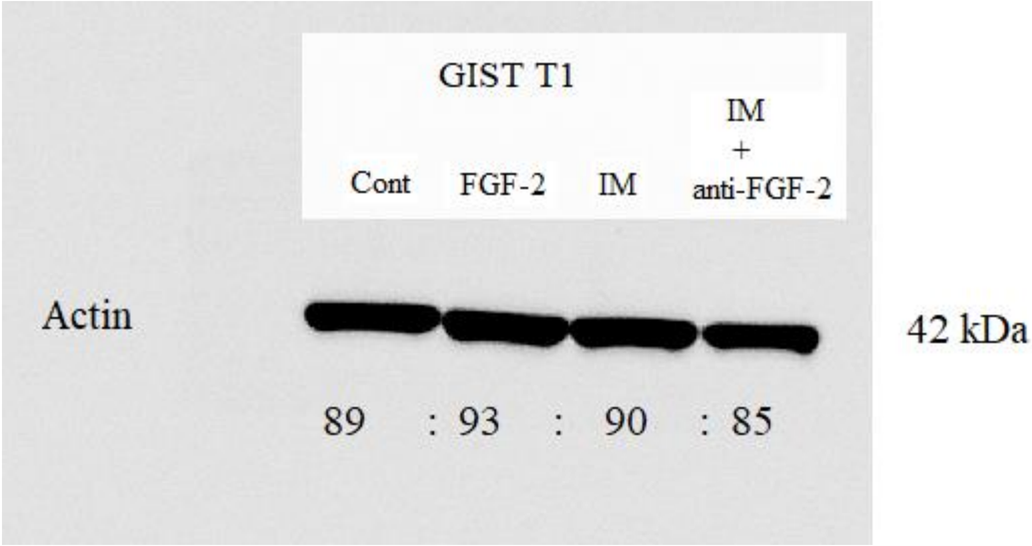
GIST T1
Cont
FGF-2
IM
IM + anti-FGF-2
Actin
42 kDa
89 : 93 : 90 : 85

Figure 3

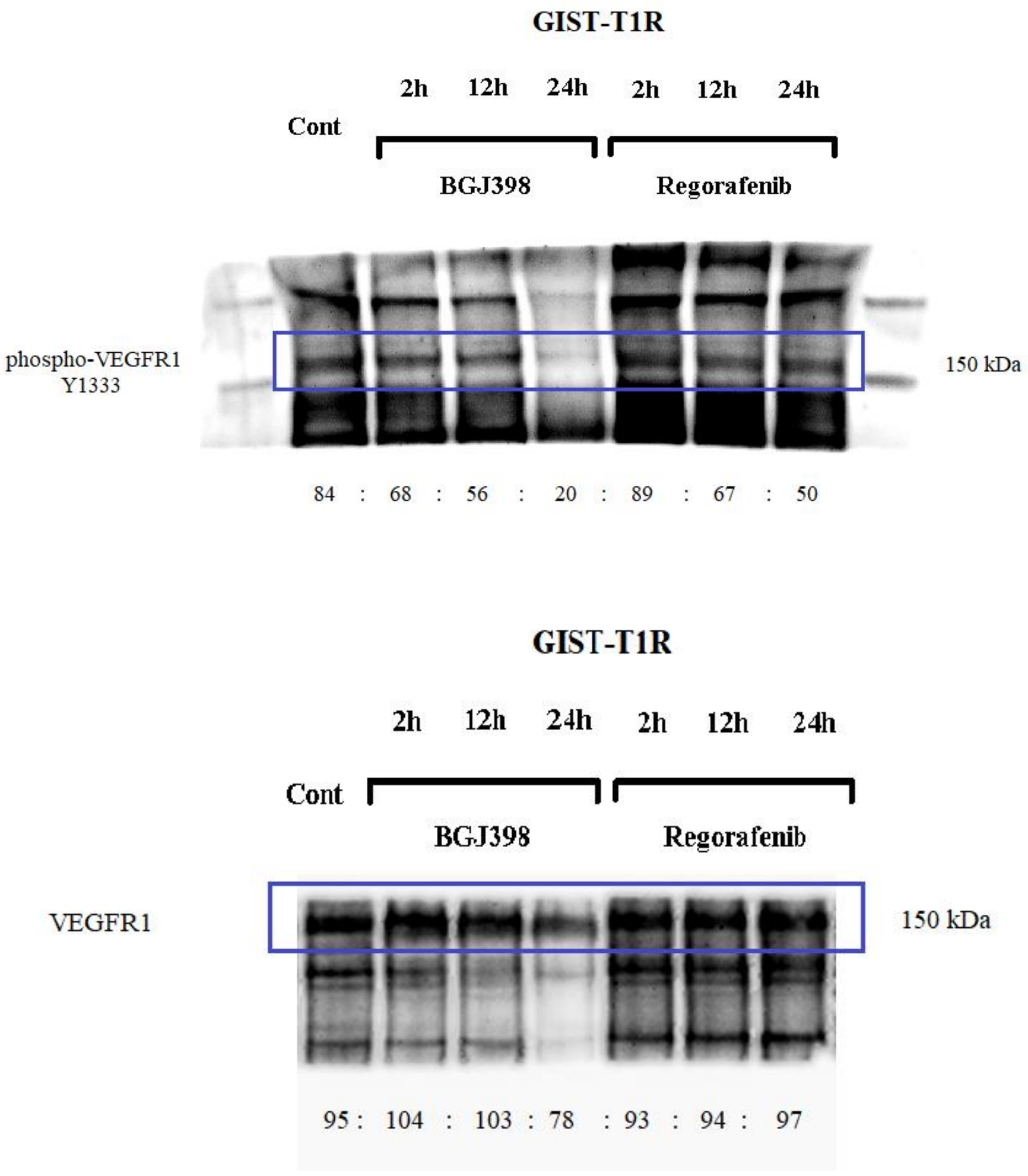

GIST-T1R
2h 12h 24h 2h 12h 24h
Cont
BGJ398
Regorafenib
phospho-VEGFR1
Y1333
150 kDa
84 : 68 : 56 : 20 : 89 : 67 : 50
GIST-T1R
2h 12h 24h 2h 12h 24h
Cont
BGJ398
Regorafenib
VEGFR1
150 kDa
95 : 104 : 103 : 78 : 93 : 94 : 97

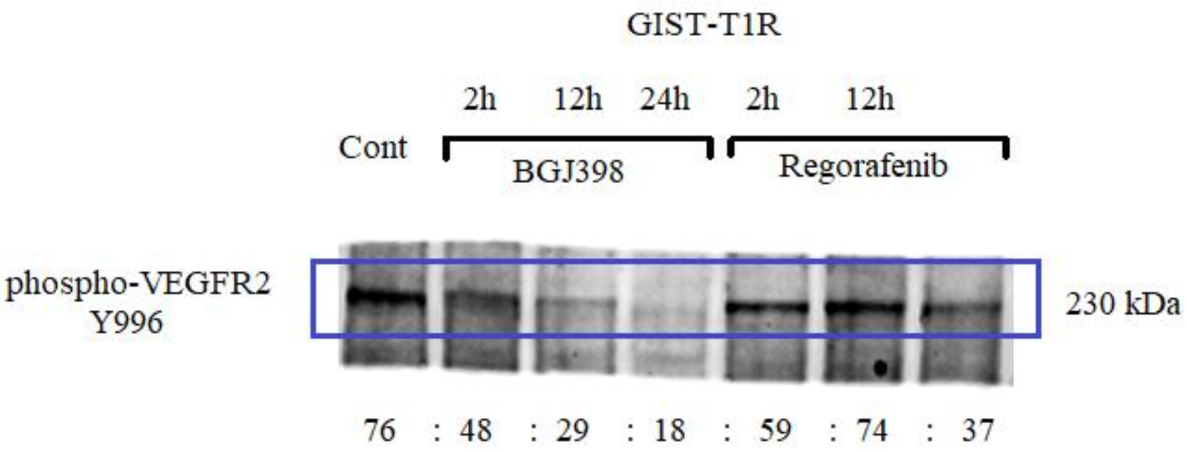
GIST-T1R
2h
12h
24h
2h
12h
Cont
BGJ398
Regorafenib
phospho-VEGFR2 Y996
230 kDa
76 : 48 : 29 : 18 : 59 : 74 : 37

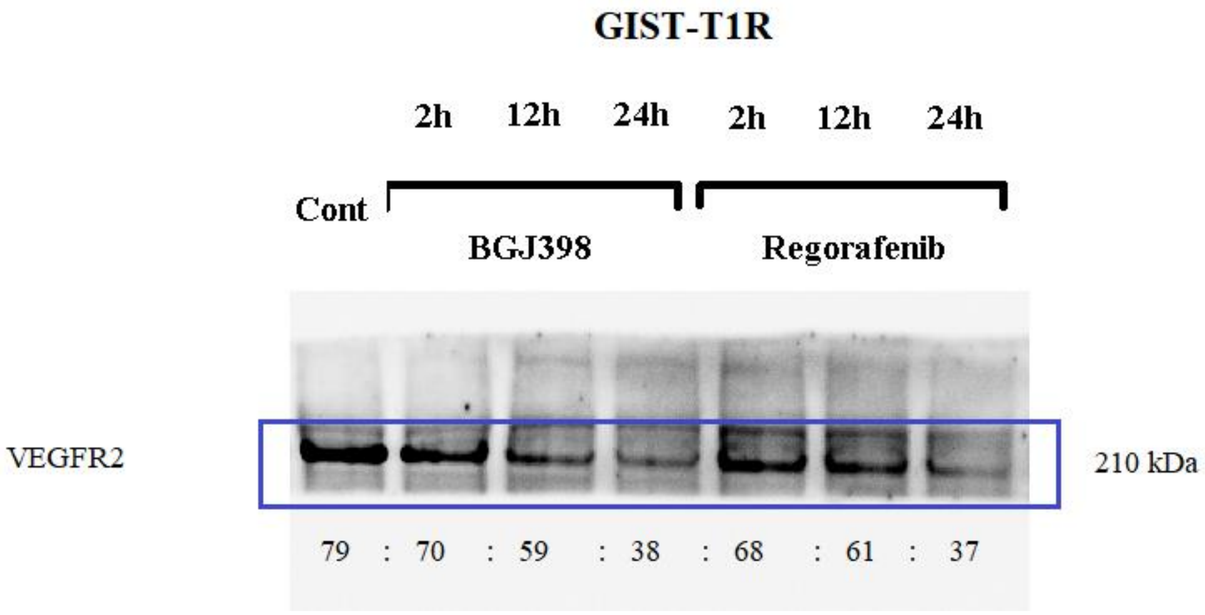
GIST-T1R
2h
12h
24h
2h
12h
24h
Cont
BGJ398
Regorafenib
VEGFR2
210 kDa
79 : 70 : 59 : 38 : 68 : 61 : 37

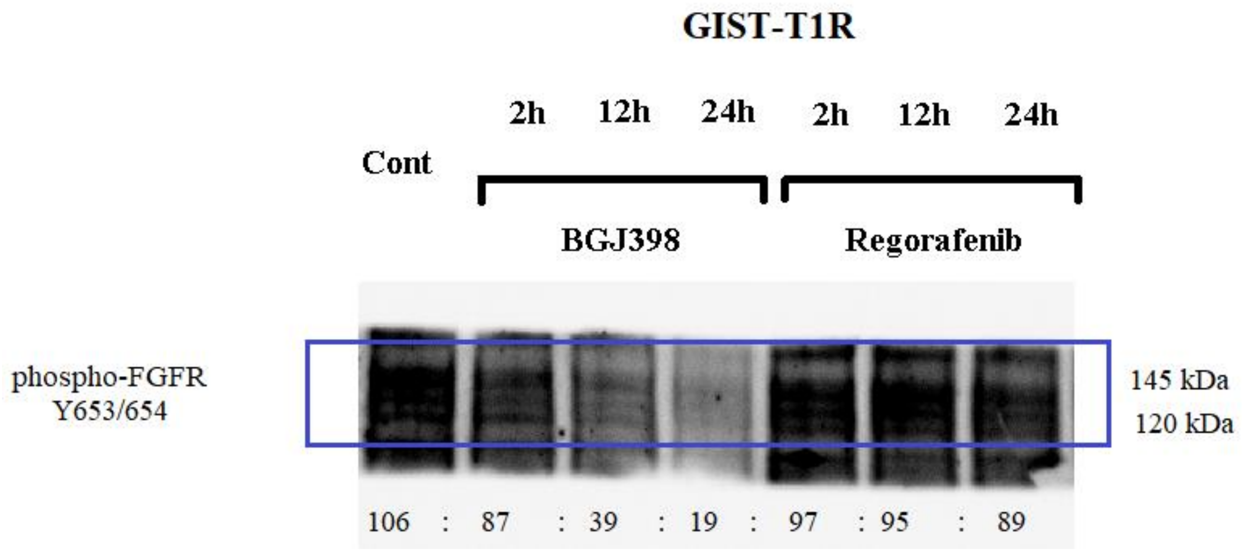
GIST-T1R
2h
12h
24h
2h
12h
24h
Cont
BGJ398
Regorafenib
phospho-FGFR
Y653/654
145 kDa
120 kDa
106 : 87 : 39 : 19 : 97 : 95 : 89

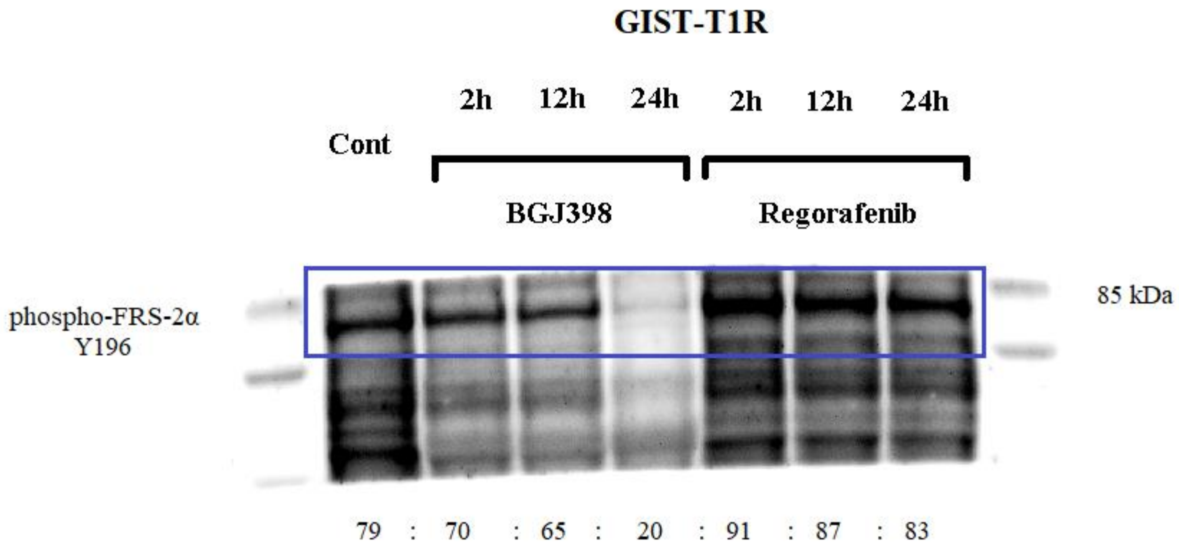
GIST-T1R
2h
12h
24h
2h
12h
24h
Cont
BGJ398
Regorafenib
phospho-FRS-2α
Y196
85 kDa
79 : 70 : 65 : 20 : 91 : 87 : 83

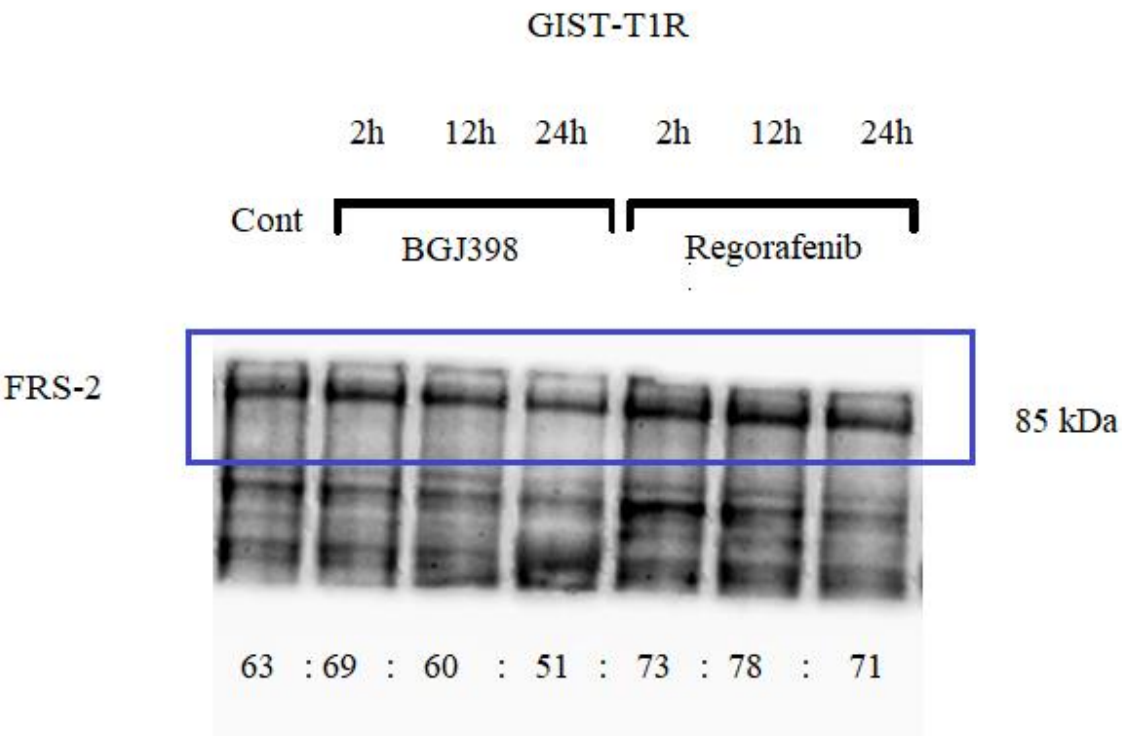
GIST-T1R
2h
12h
24h
2h
12h
24h
Cont
BGJ398
Regorafenib
FRS-2
85 kDa
63 : 69 : 60 : 51 : 73 : 78 : 71

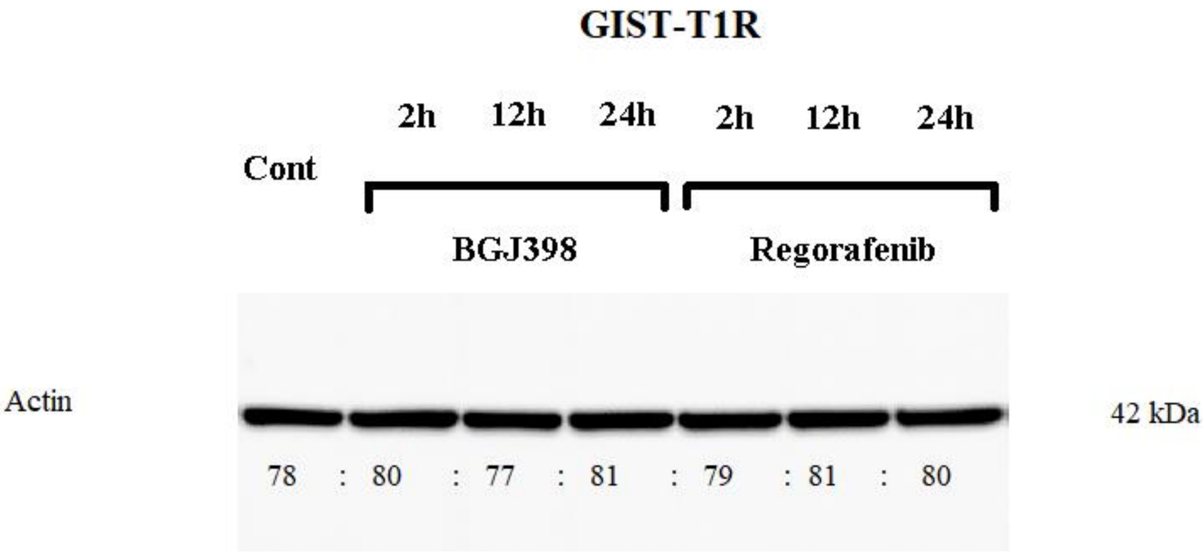
GIST-T1R
2h
12h
24h
2h
12h
24h
Cont
BGJ398
Regorafenib
Actin
42 kDa
78 : 80 : 77 : 81 : 79 : 81 : 80

Figure 4 D.

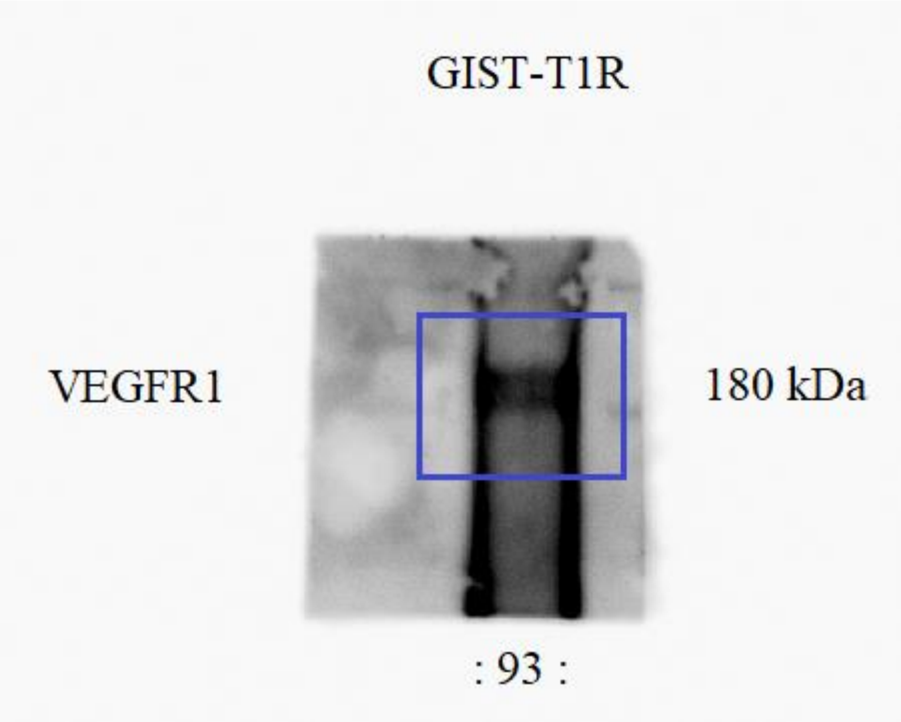
GIST-T1R
VEGFR1
180 kDa
: 93 :

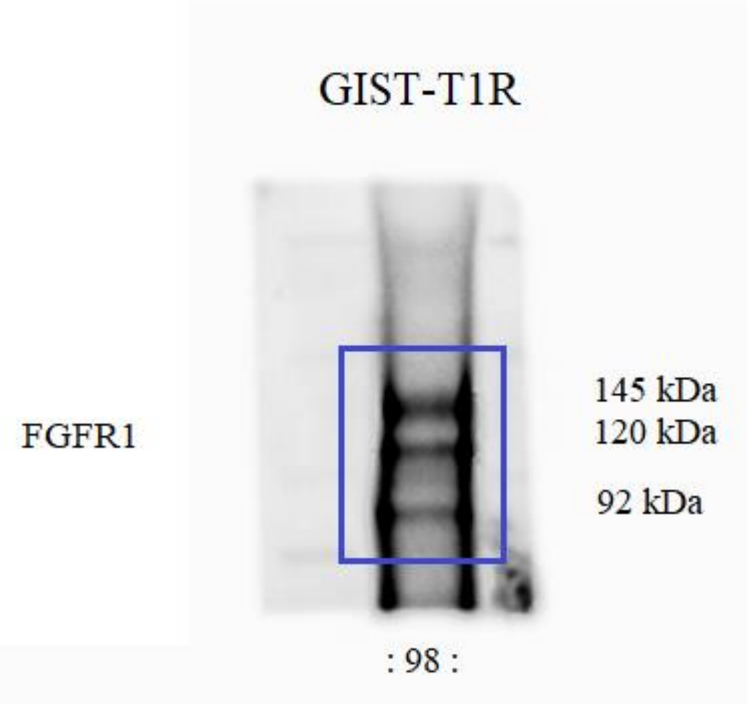
GIST-T1R
FGFR1
145 kDa
120 kDa
92 kDa
: 98 :

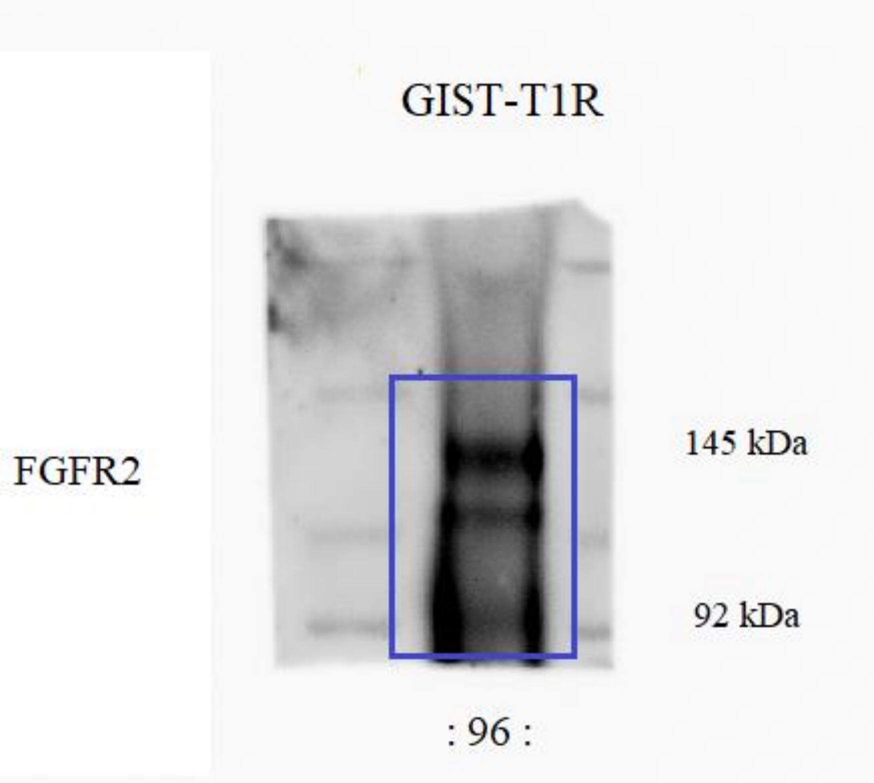
GIST-T1R
FGFR2
145 kDa
92 kDa
: 96 :

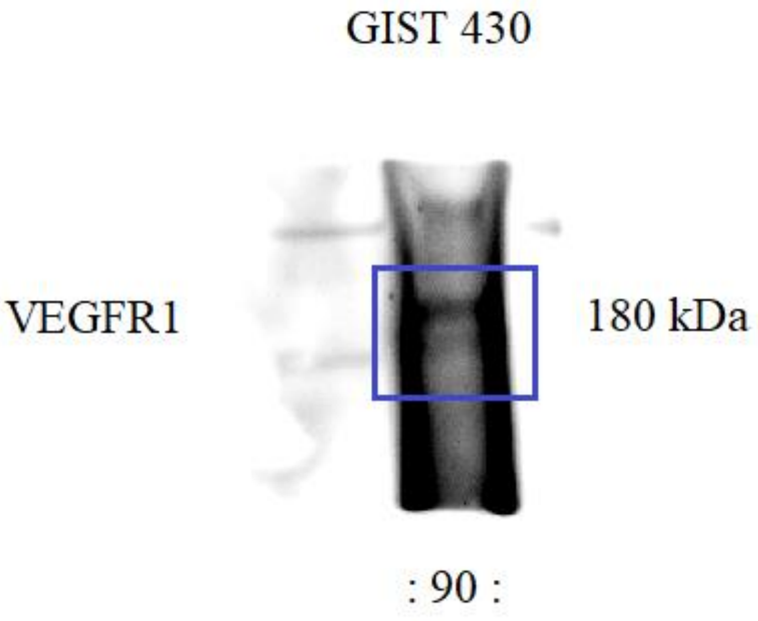
GIST 430
VEGFR1
180 kDa
: 90 :

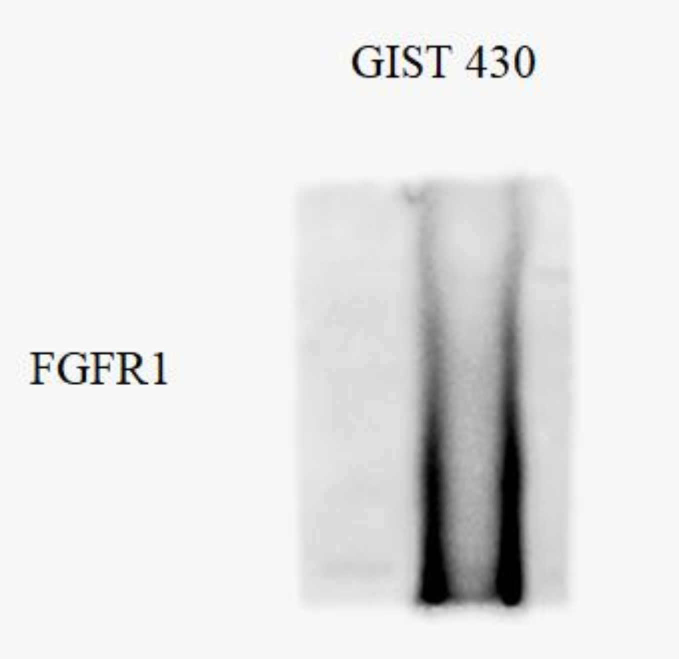
GIST 430
FGFR1

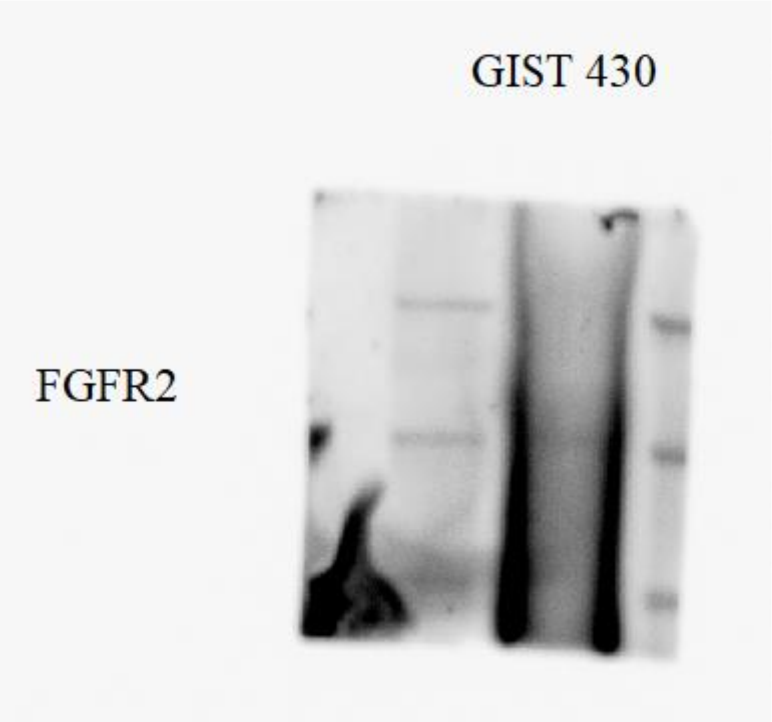
GIST 430
FGFR2

Figure 5 A.

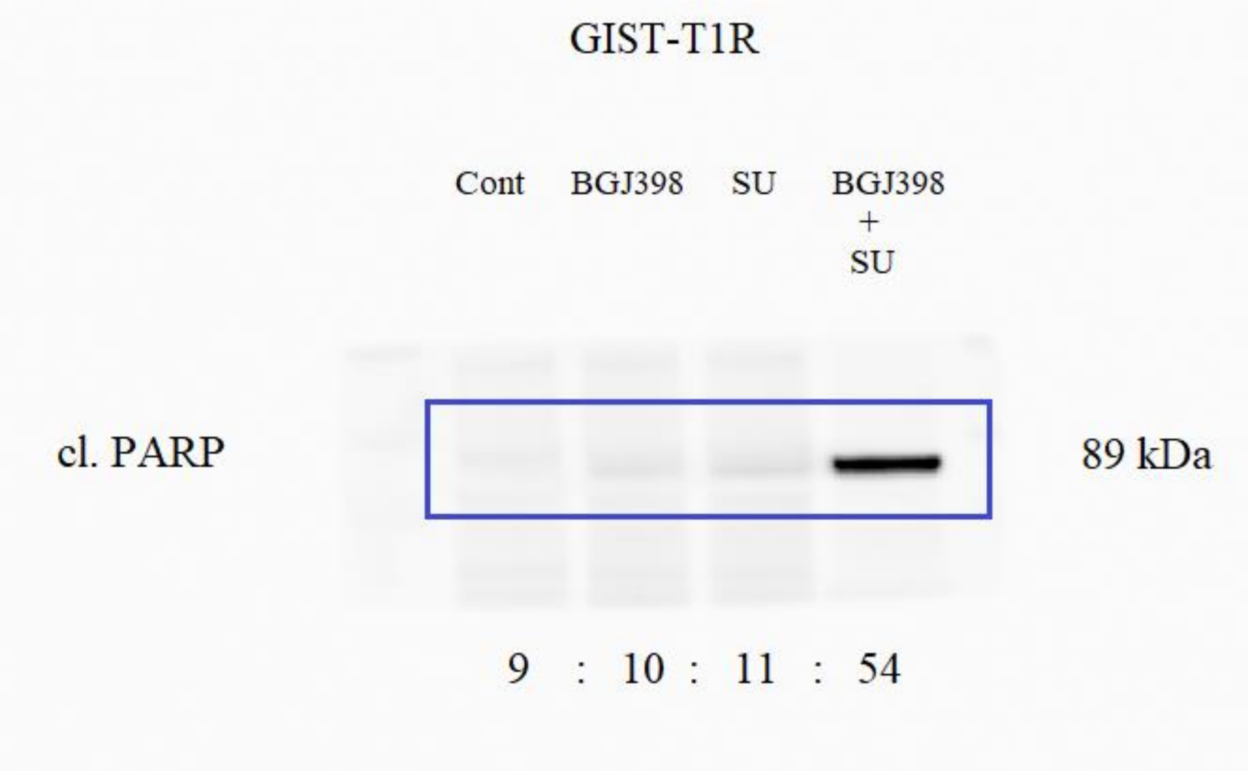
GIST-T1R
Cont
BGJ398
SU
BGJ398
+
SU
cl. PARP
89 kDa
9 : 10 : 11 : 54

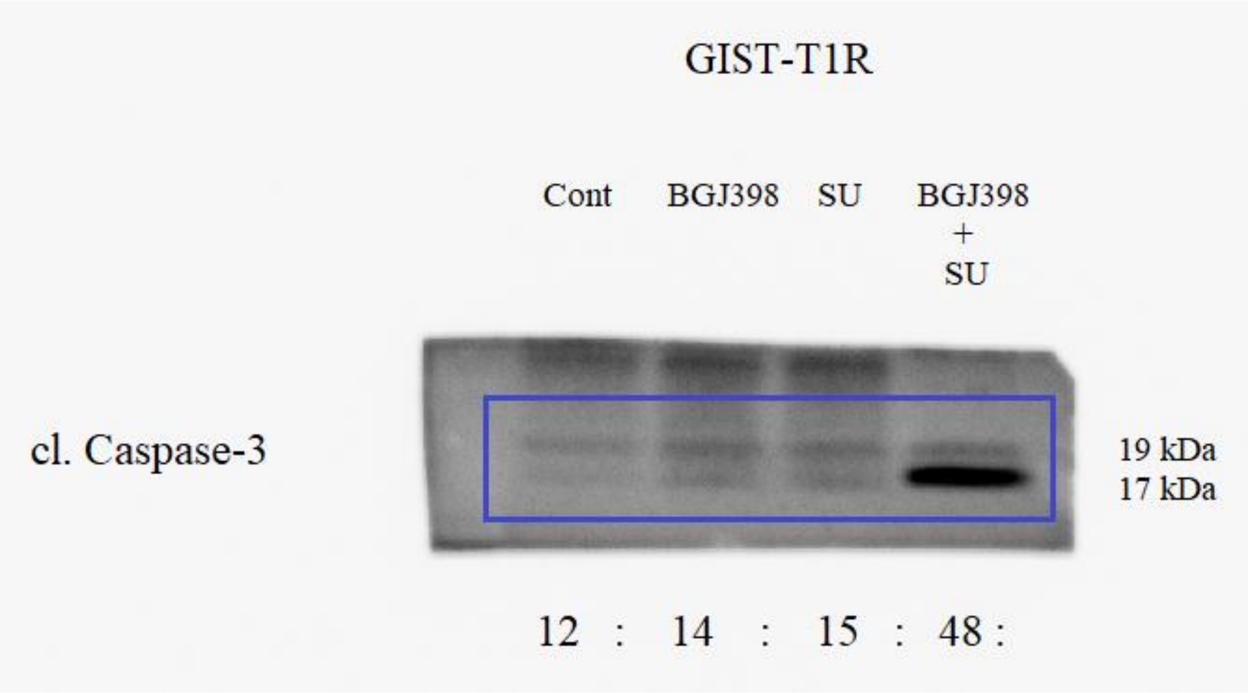
GIST-T1R
Cont
BGJ398
SU
BGJ398
+
SU
cl. Caspase-3
19 kDa
17 kDa
12 : 14 : 15 : 48 :

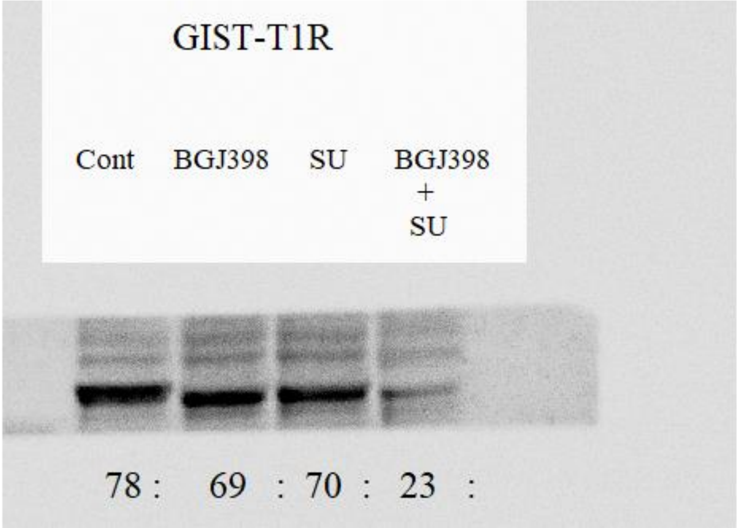
GIST-T1R
Cont
BGJ398
SU
BGJ398 + SU
phospho-AKT S473
60 kDa
78 : 69 : 70 : 23 :

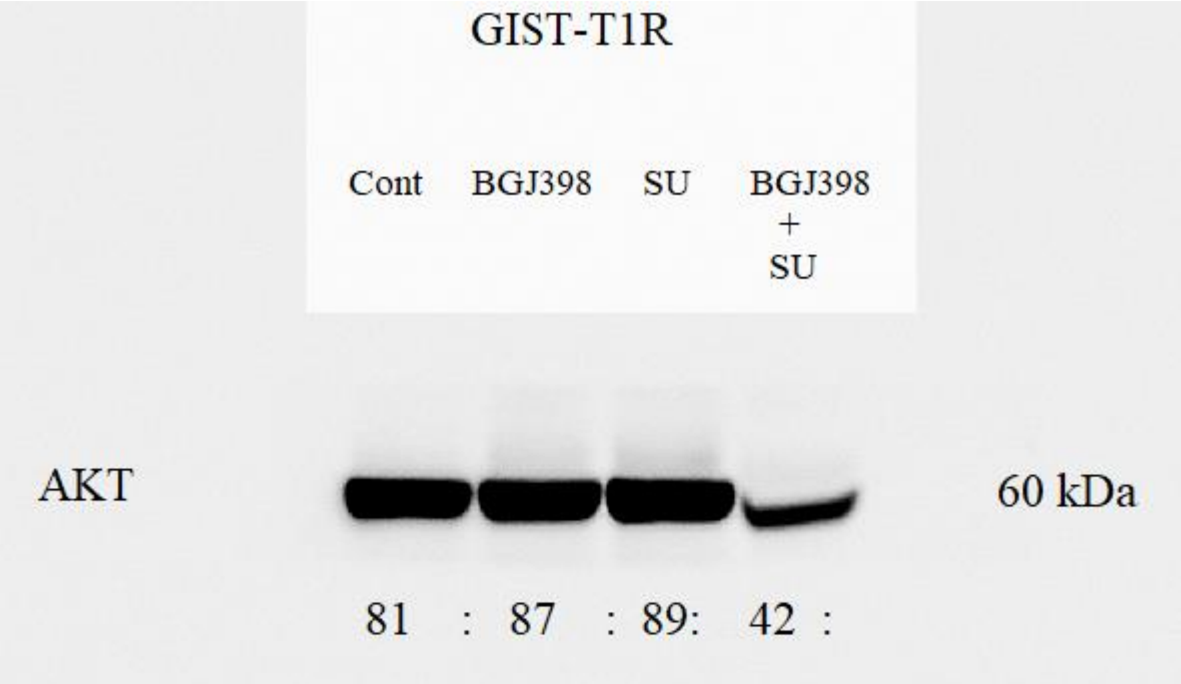
GIST-T1R
Cont
BGJ398
SU
BGJ398 + SU
AKT
60 kDa
81 : 87 : 89: 42 :

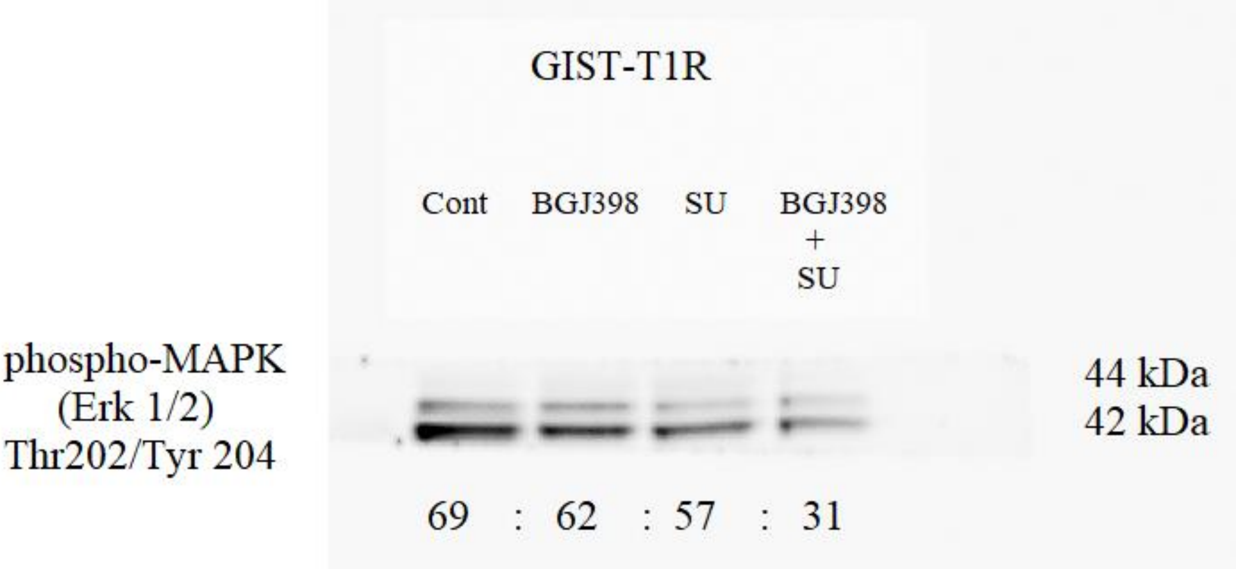
GIST-T1R
Cont
BGJ398
SU
BGJ398 + SU
phospho-MAPK (Erk 1/2) Thr202/Tyr 204
44 kDa
42 kDa
69 : 62 : 57 : 31

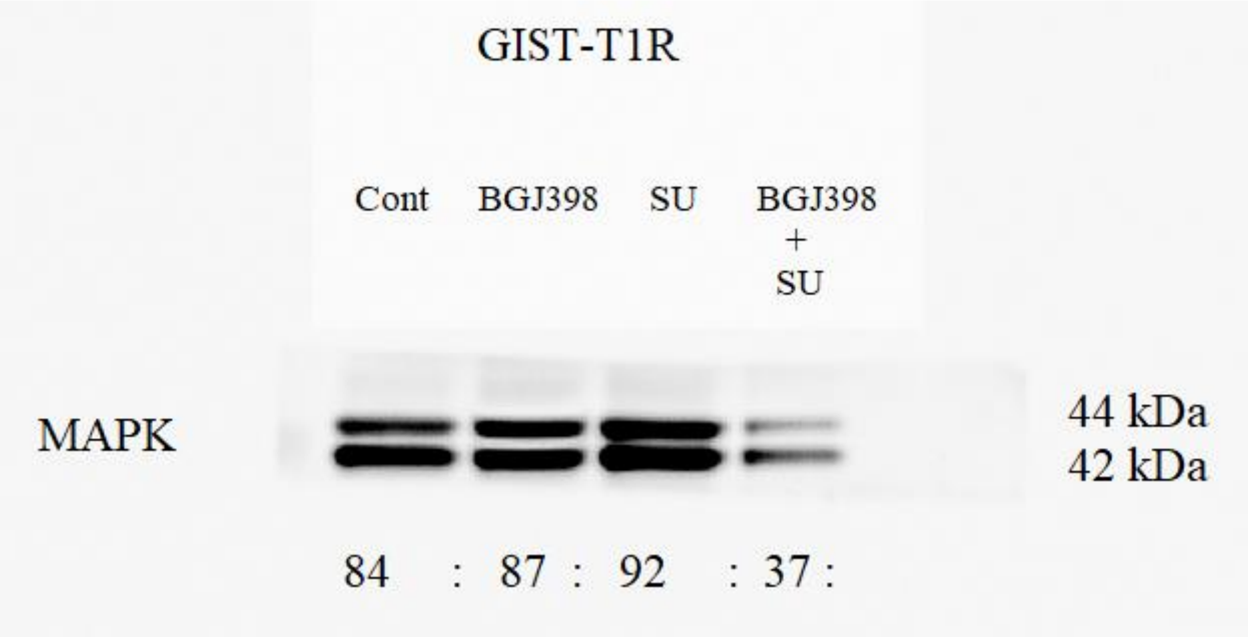
GIST-T1R
Cont
BGJ398
SU
BGJ398 + SU
MAPK
44 kDa
42 kDa
84 : 87 : 92 : 37 :

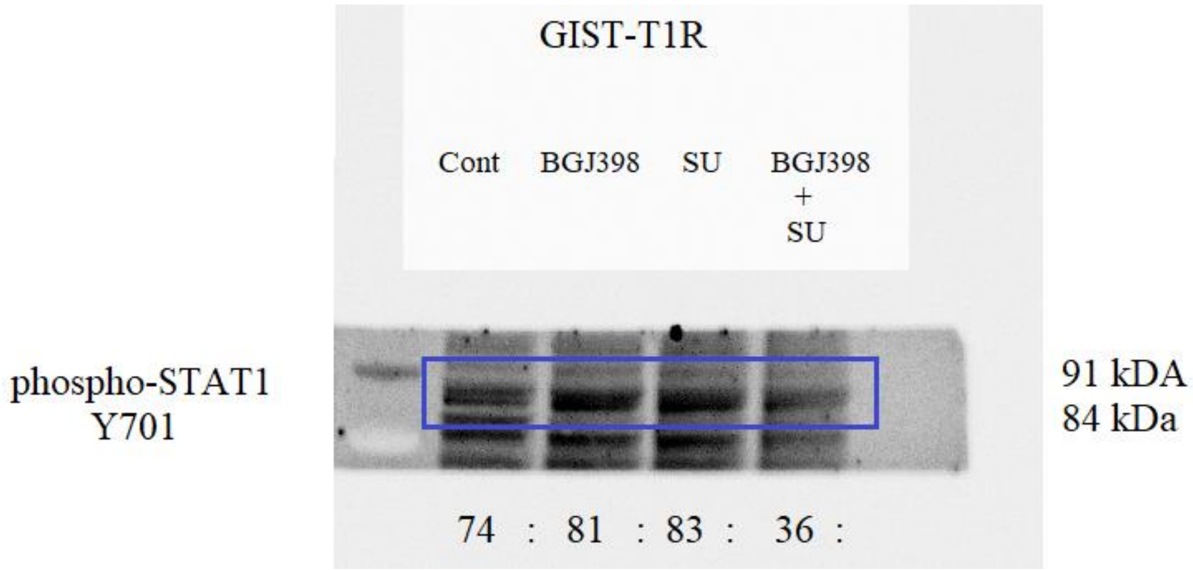
GIST-T1R
Cont
BGJ398
SU
BGJ398
+
SU
phospho-STAT1
Y701
91 kDA
84 kDa
74 : 81 : 83 : 36 :

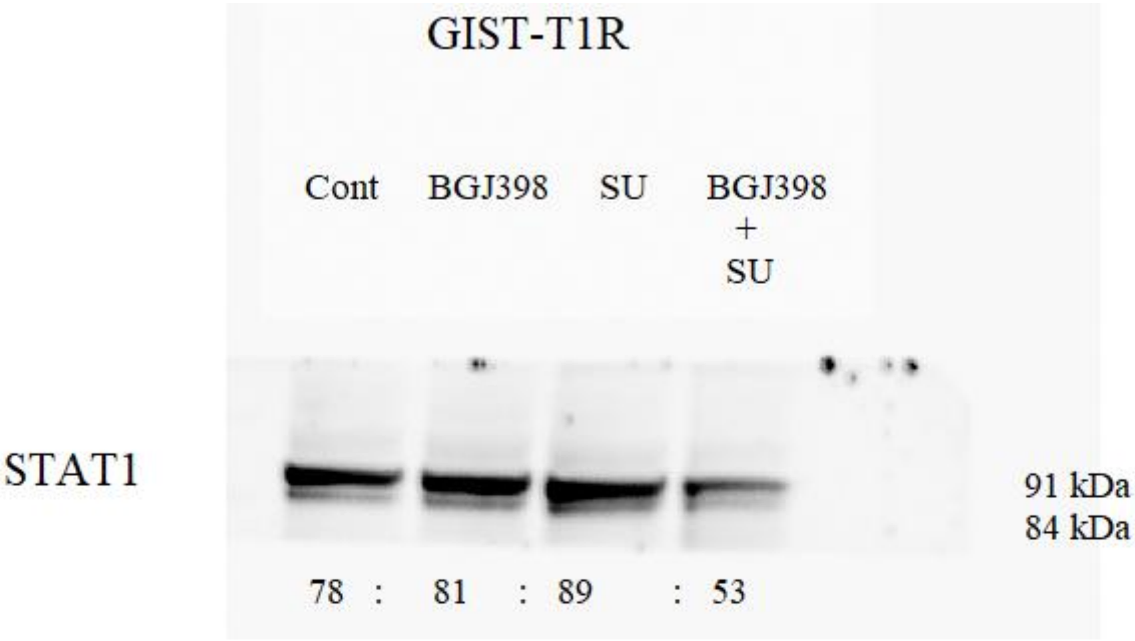
GIST-T1R
Cont
BGJ398
SU
BGJ398
+
SU
STAT1
91 kDa
84 kDa
78 : 81 : 89 : 53

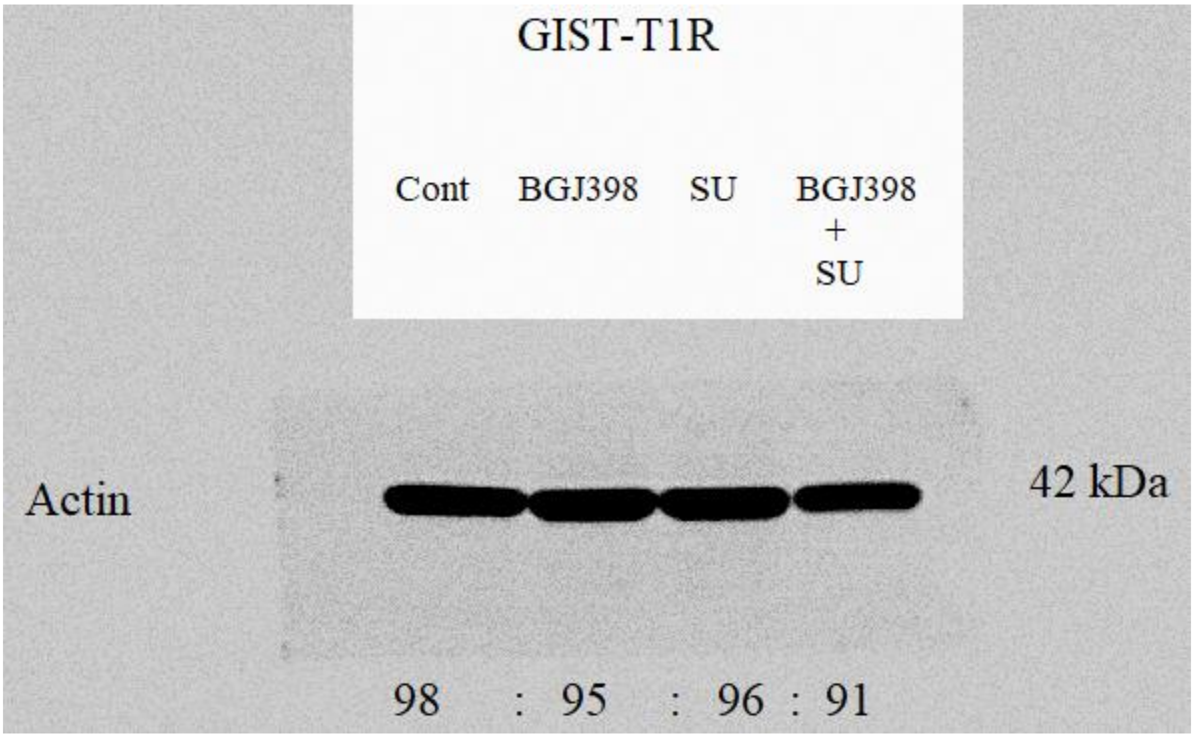
GIST-T1R
Cont
BGJ398
SU
BGJ398
+
SU
Actin
42 kDa
98 : 95 : 96 : 91

Figure 5 B.

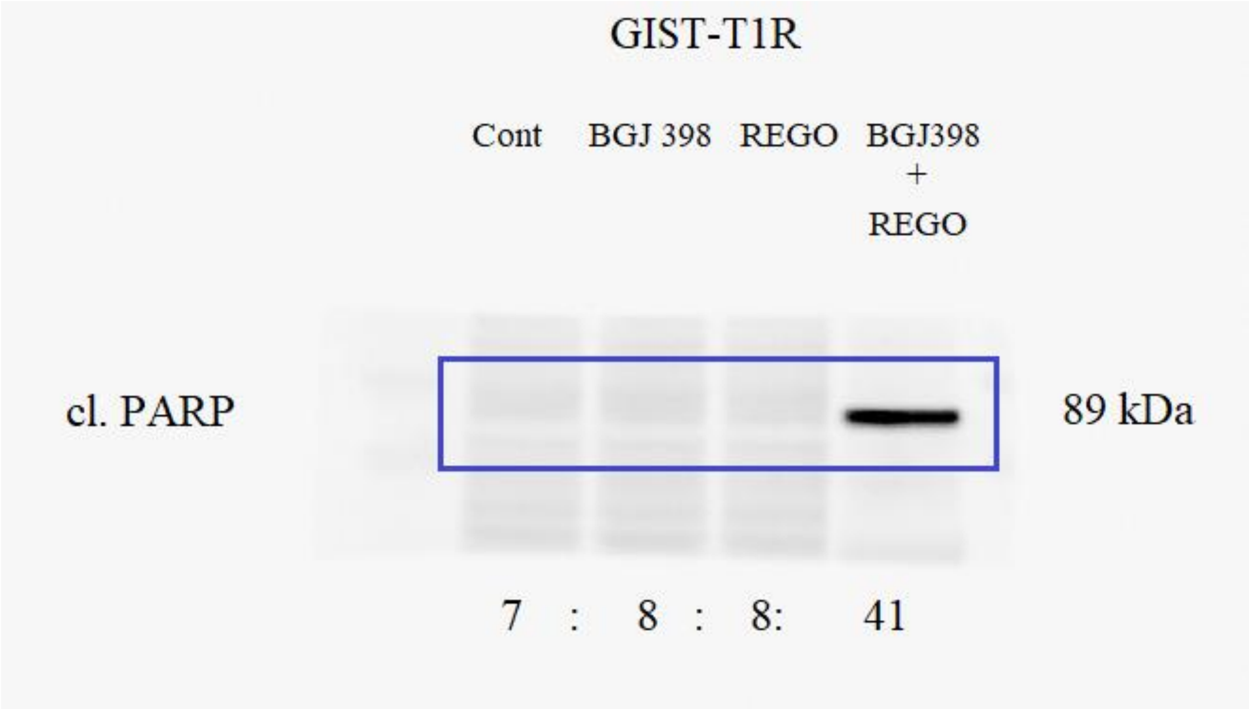
GIST-T1R
Cont
BGJ 398
REGO
BGJ398
+
REGO
cl. PARP
89 kDa
7 : 8 : 8: 41

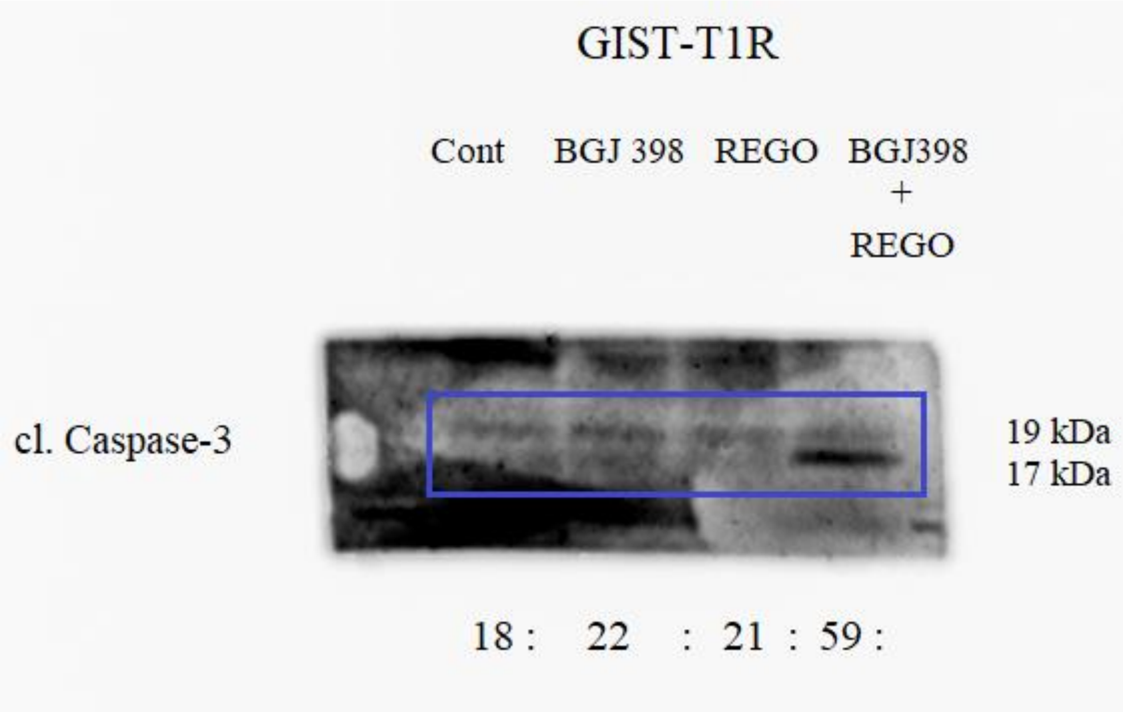
GIST-T1R
Cont
BGJ 398
REGO
BGJ398
+
REGO
cl. Caspase-3
19 kDa
17 kDa
18 : 22 : 21 : 59 :

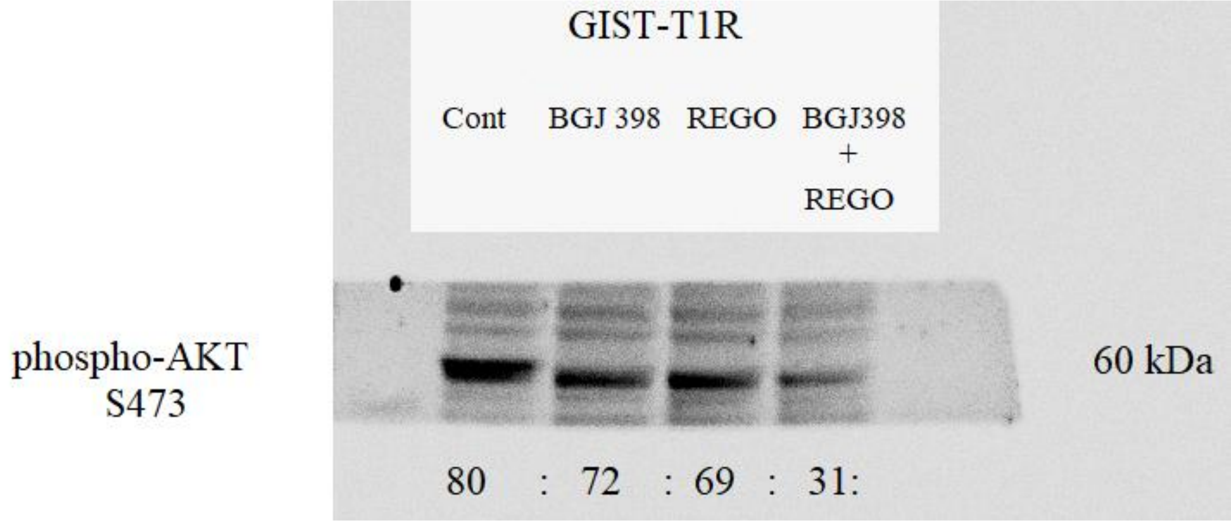
GIST-T1R
Cont
BGJ 398
REGO
BGJ398
+
REGO
phospho-AKT
S473
60 kDa
80 : 72 : 69 : 31:

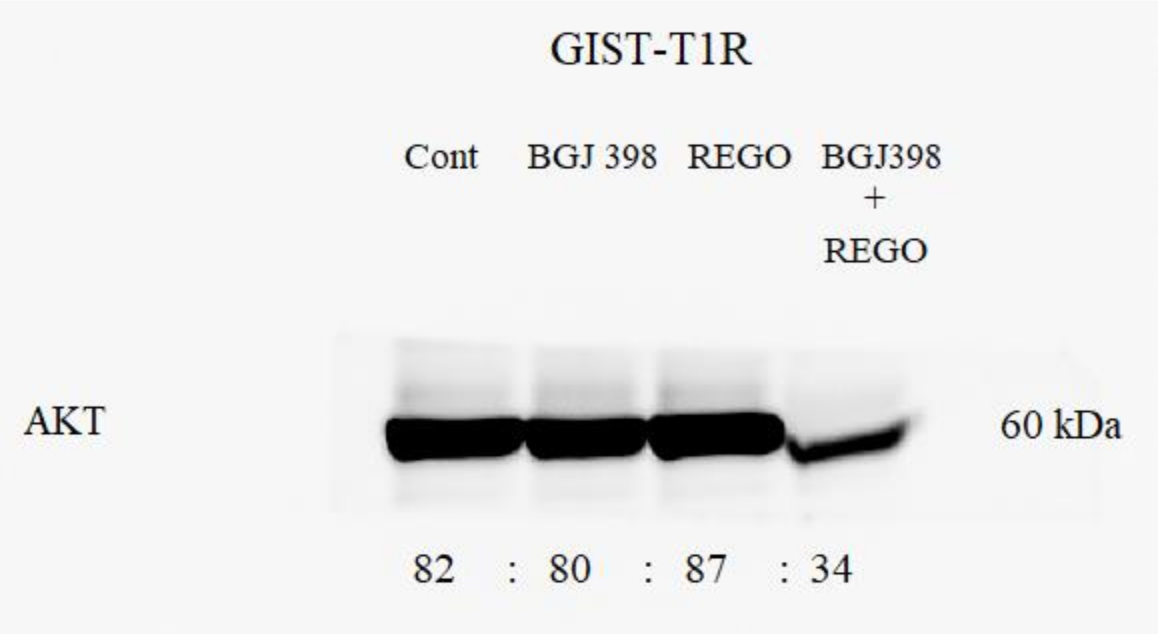
GIST-T1R
Cont
BGJ 398
REGO
BGJ398
+
REGO
AKT
60 kDa
82 : 80 : 87 : 34

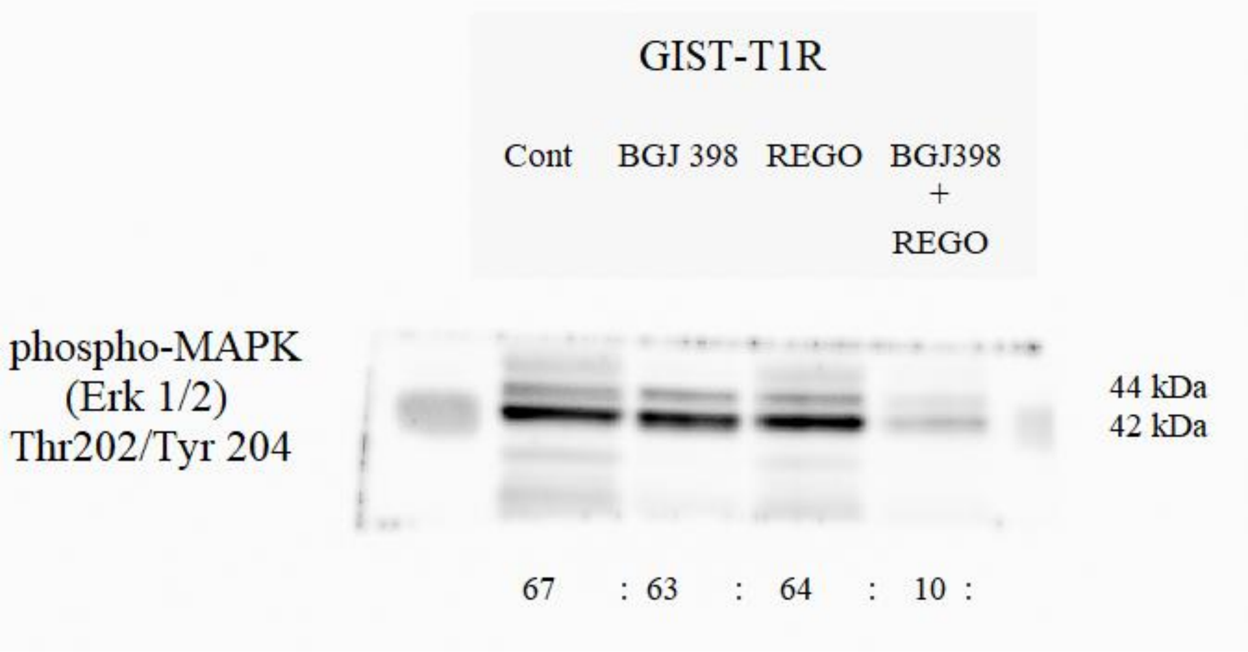
GIST-T1R
Cont
BGJ 398
REGO
BGJ398
+
REGO
phospho-MAPK
(Erk 1/2)
Thr202/Tyr 204
44 kDa
42 kDa
67 : 63 : 64 : 10 :

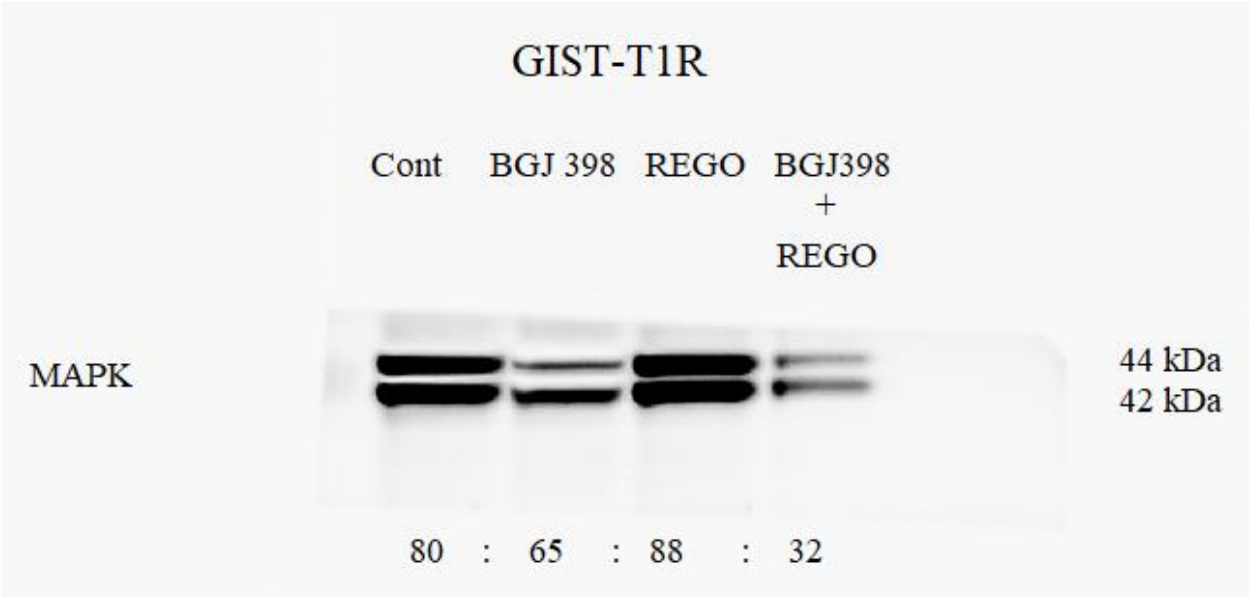
GIST-T1R
Cont
BGJ 398
REGO
BGJ398
+
REGO
MAPK
44 kDa
42 kDa
80 : 65 : 88 : 32

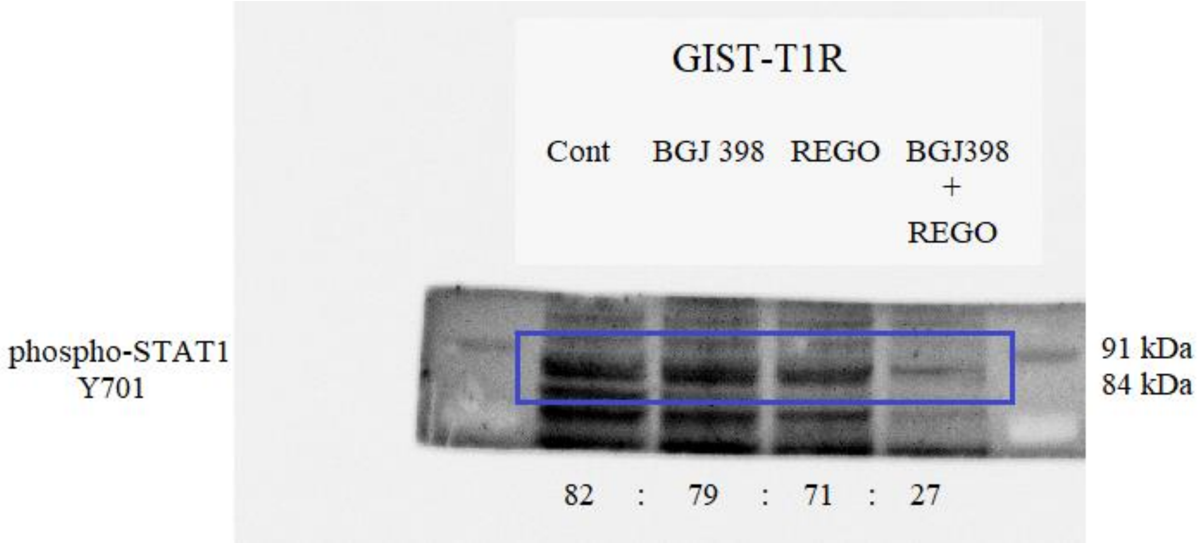
GIST-T1R
Cont
BGJ 398
REGO
BGJ398
+
REGO
phospho-STAT1
Y701
91 kDa
84 kDa
82 : 79 : 71 : 27

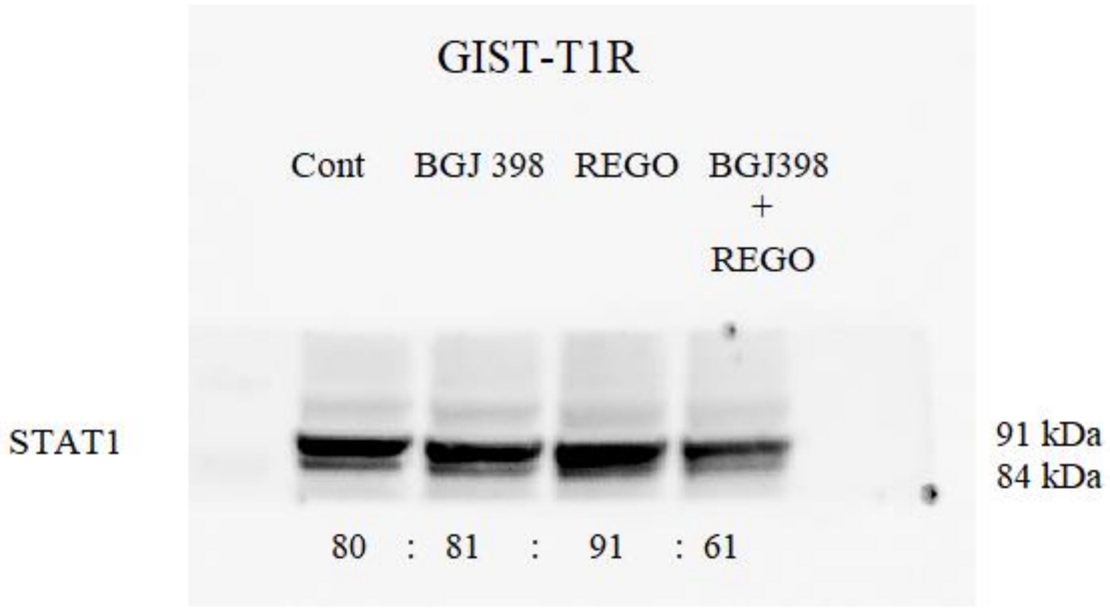
GIST-T1R
Cont
BGJ 398
REGO
BGJ398
+
REGO
STAT1
91 kDa
84 kDa
80 : 81 : 91 : 61

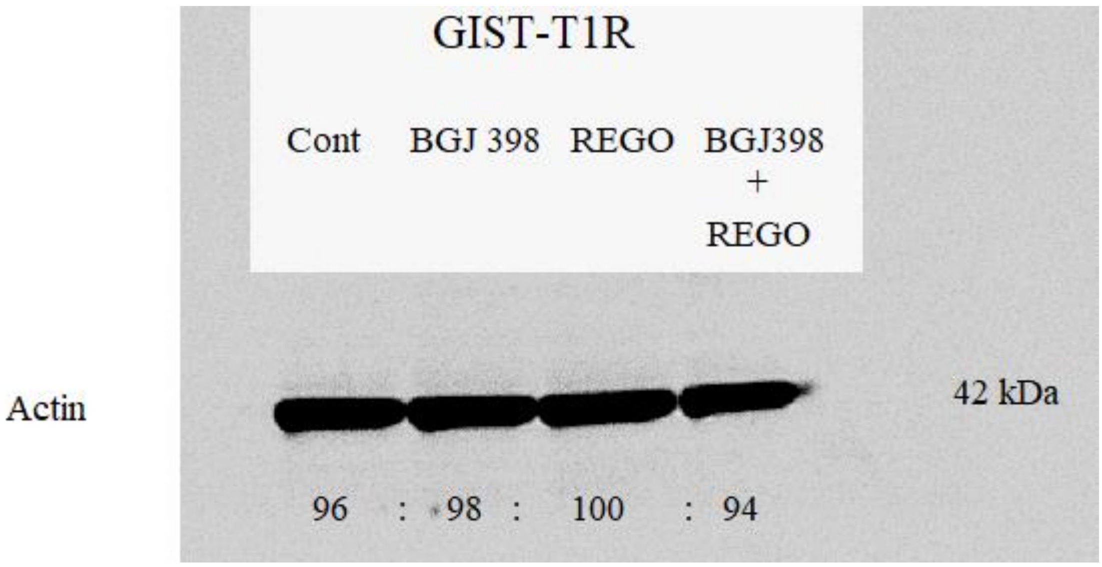
GIST-T1R
Cont
BGJ 398
REGO
BGJ398
+
REGO
Actin
42 kDa
96 : 98 : 100 : 94

Supplementary Figure 1

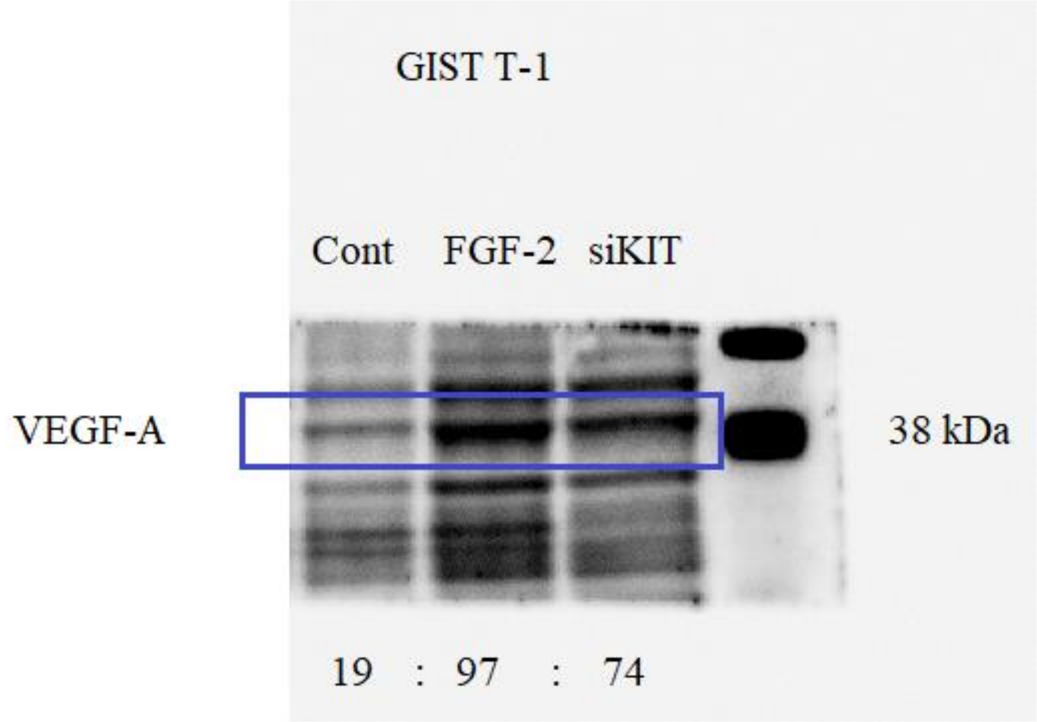

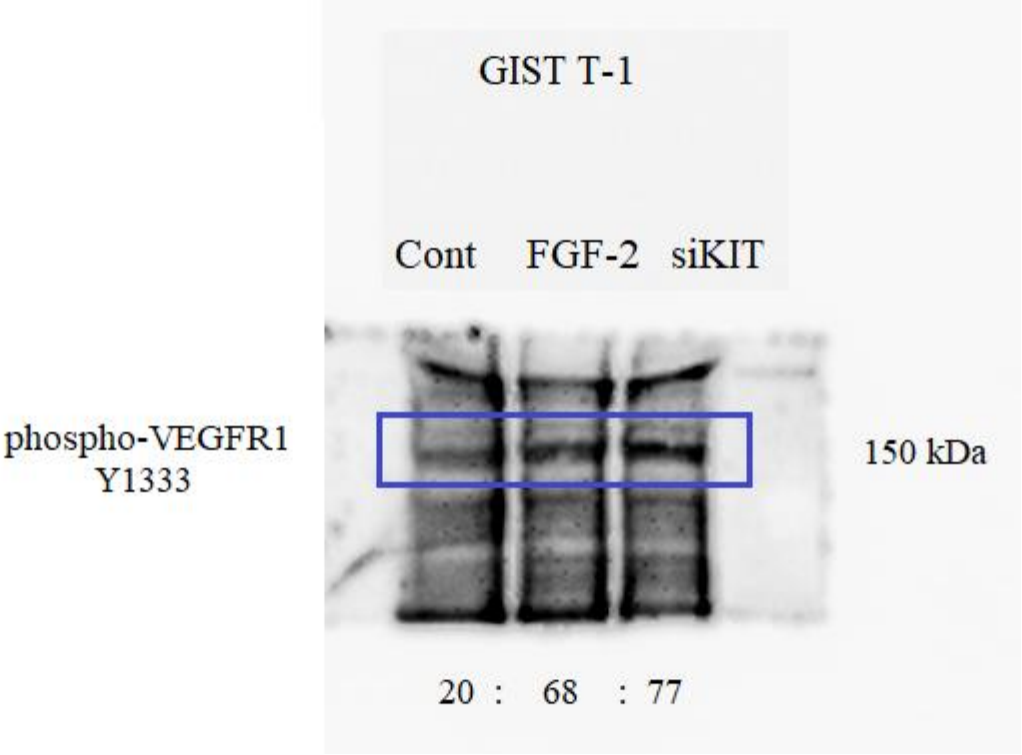

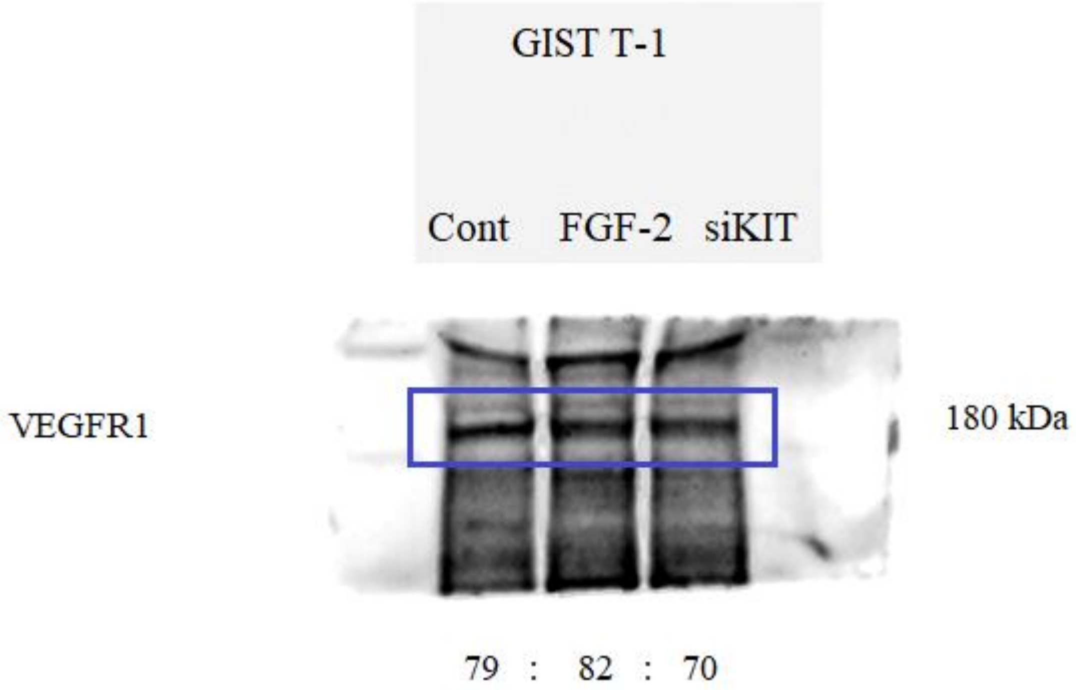
GIST T-1
Cont
FGF-2
siKIT
VEGFR1
180 kDa
79 : 82 : 70

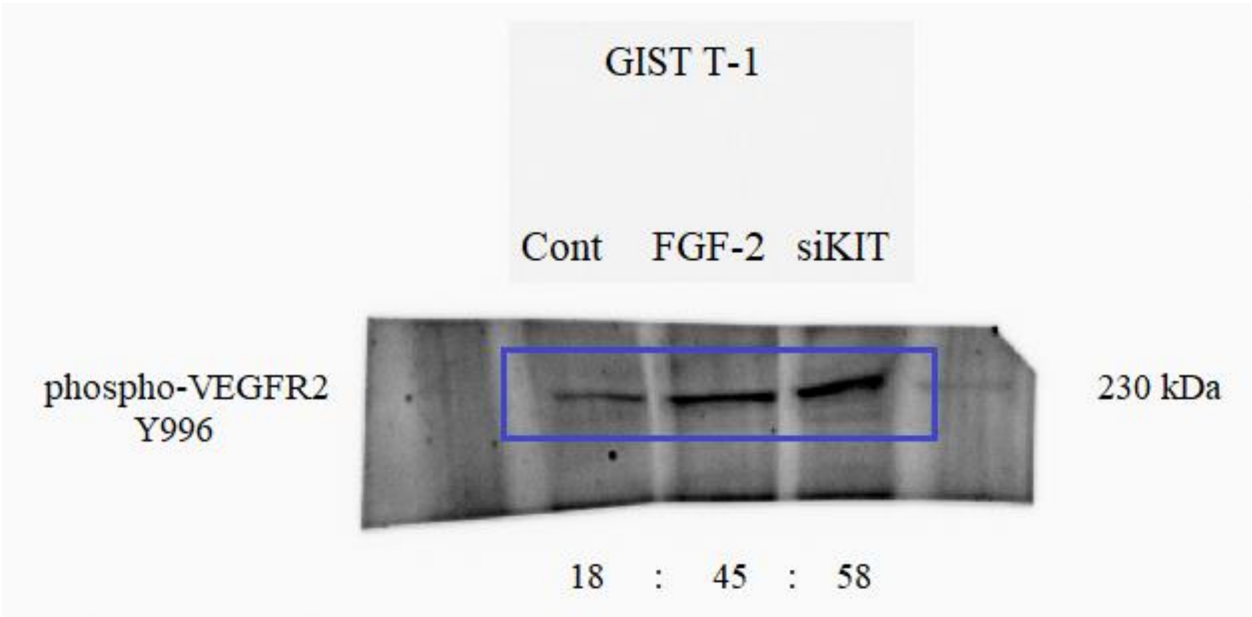
GIST T-1
Cont
FGF-2
siKIT
phospho-VEGFR2
Y996
230 kDa
18 : 45 : 58

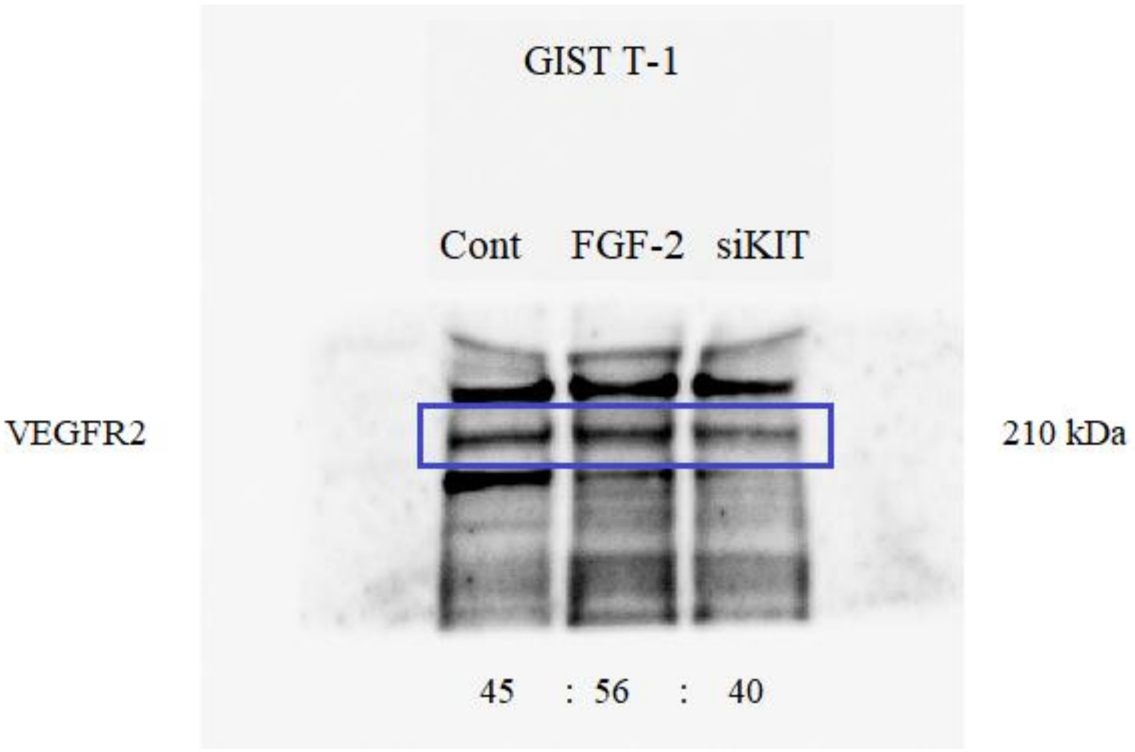
GIST T-1
Cont FGF-2 siKIT
VEGFR2
210 kDa
45 : 56 : 40

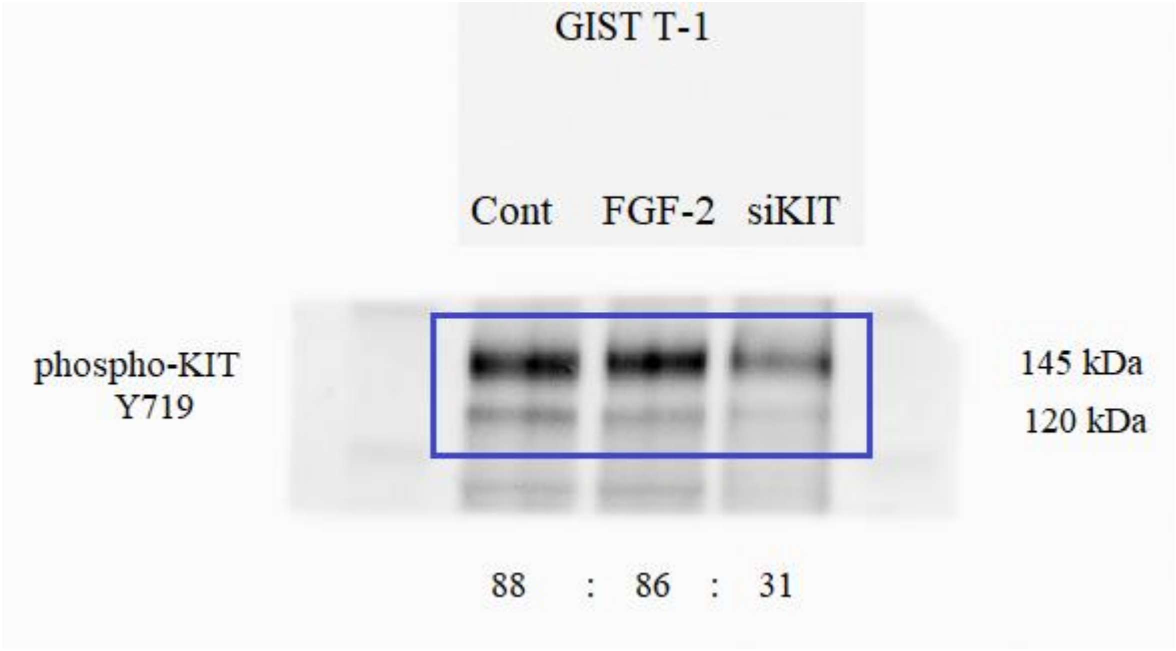
GIST T-1
Cont FGF-2 siKIT
phospho-KIT Y719
145 kDa
120 kDa
88 : 86 : 31

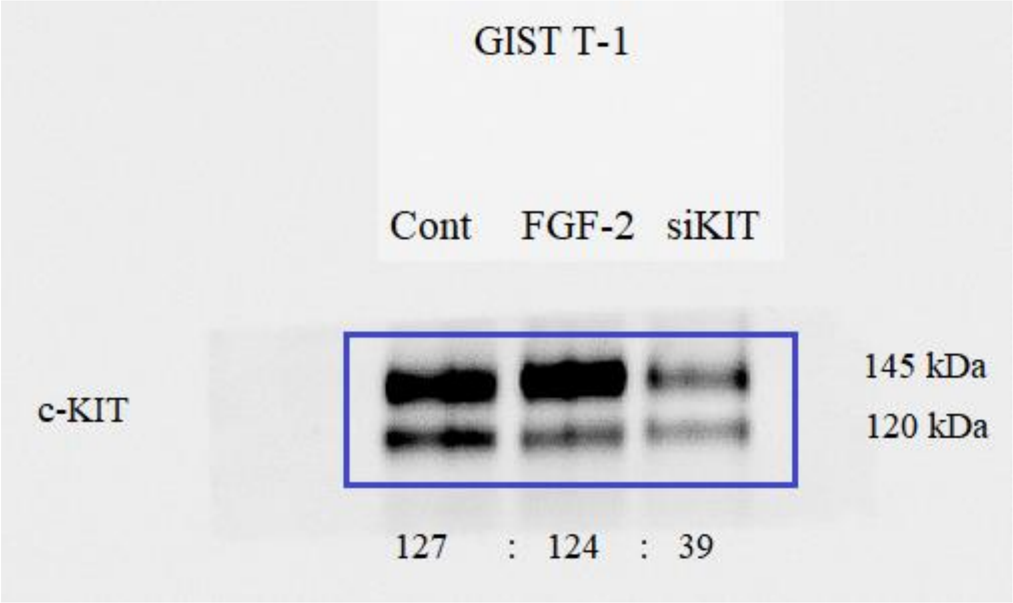
GIST T-1
Cont FGF-2 siKIT
c-KIT
145 kDa
120 kDa
127 : 124 : 39

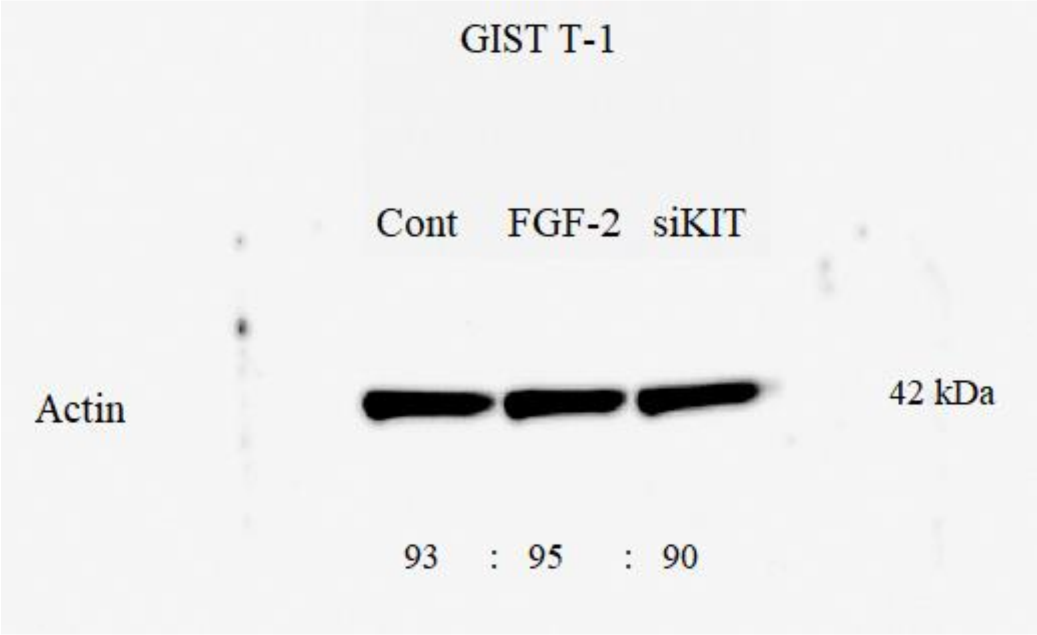
GIST T-1
Cont FGF-2 siKIT
Actin
42 kDa
93 : 95 : 90

Supplementary Figure 3 E.

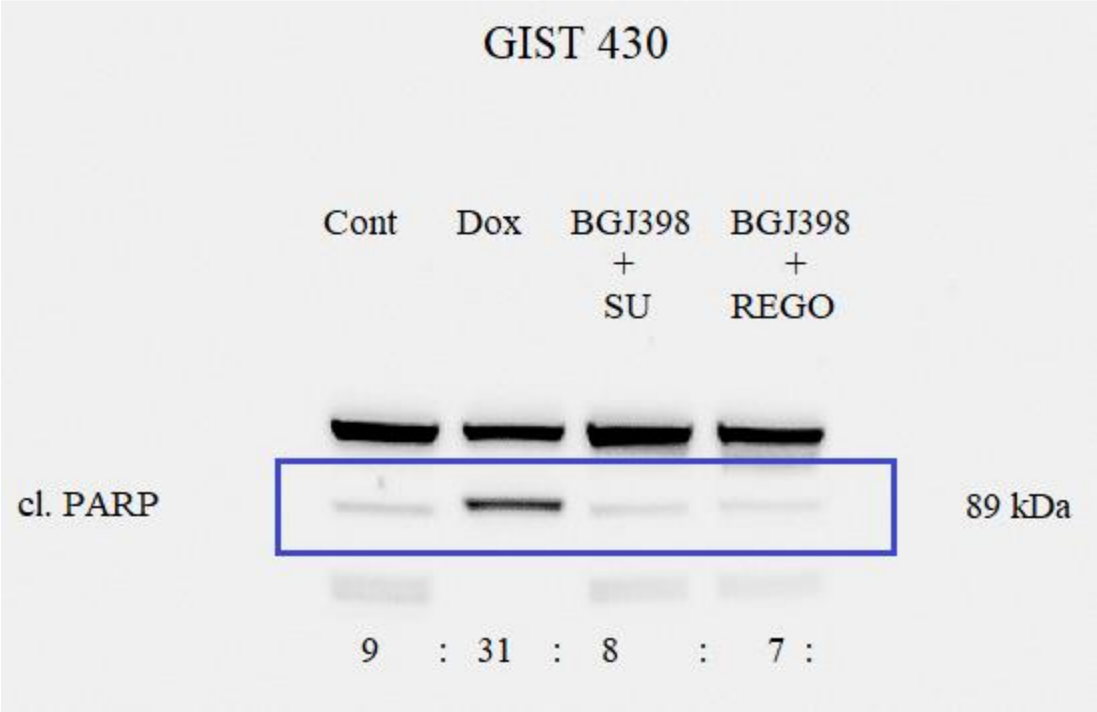

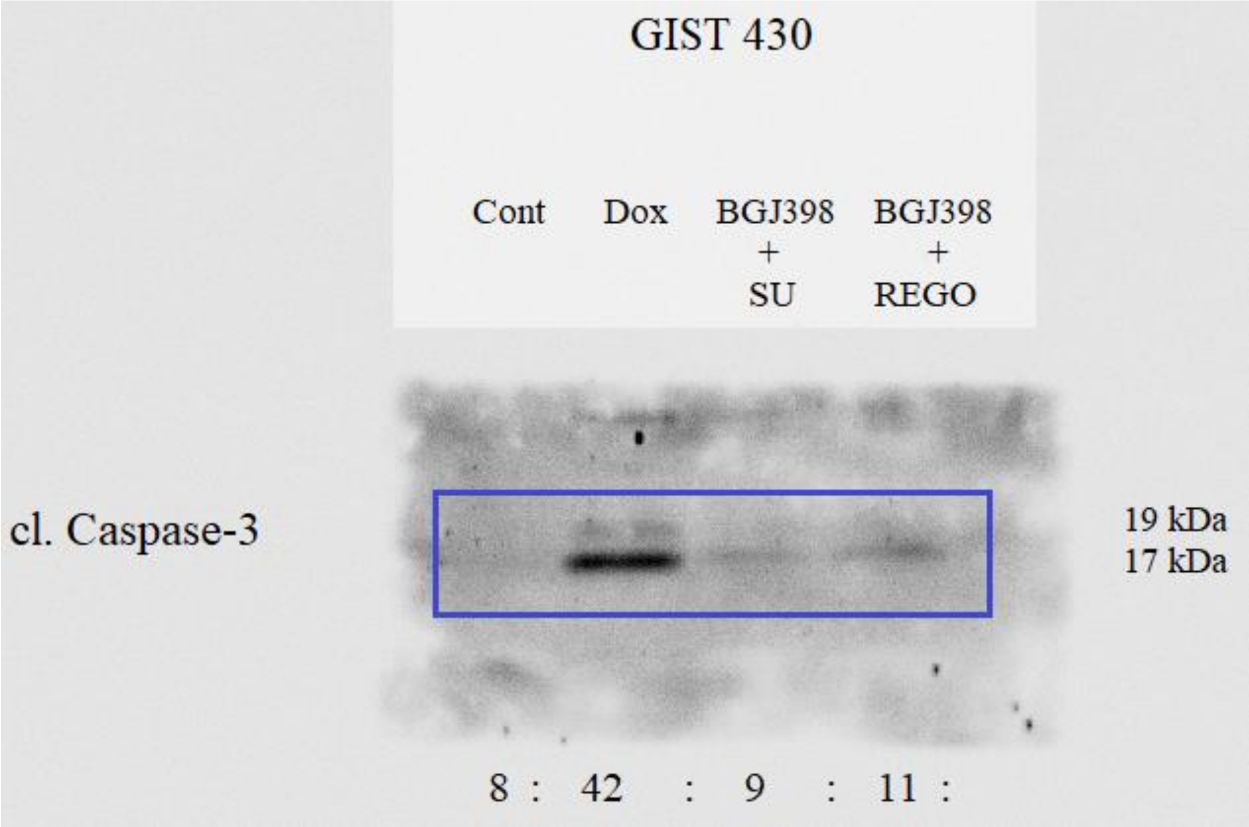
GIST 430
Cont
Dox
BGJ398 + SU
BGJ398 + REGO
cl. Caspase-3
19 kDa
17 kDa
8 : 42 : 9 : 11 :

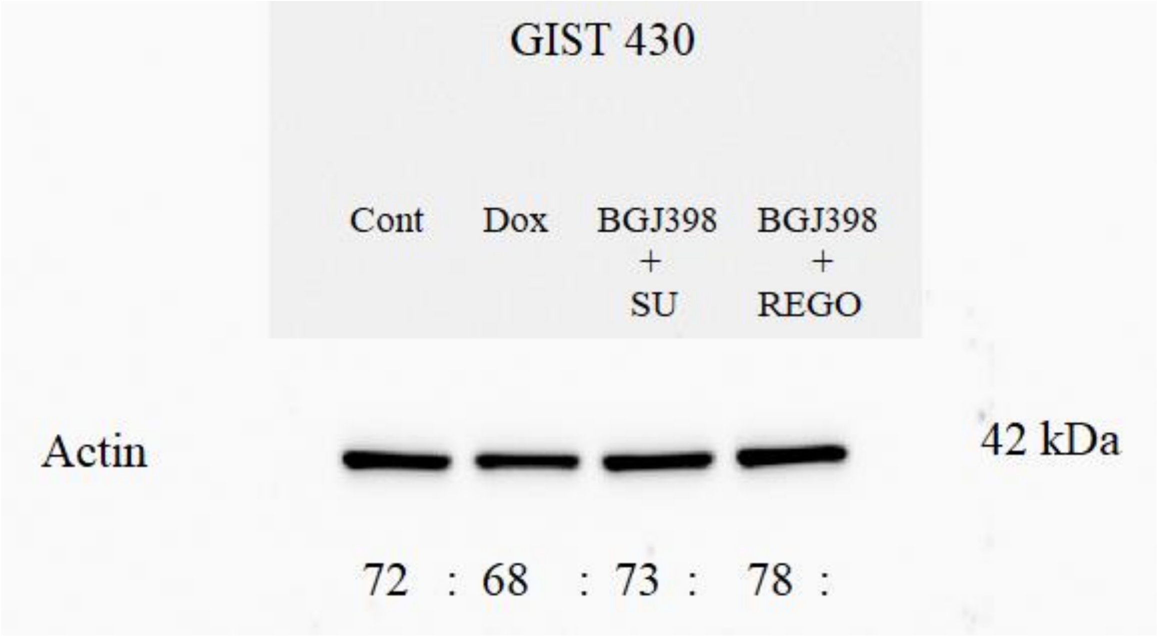
GIST 430
Cont
Dox
BGJ398 + SU
BGJ398 + REGO
Actin
42 kDa
72 : 68 : 73 : 78 :
